# The optimal exercise modality and dose for glycemic control in older adults with type 2 diabetes mellitus: a systematic review and network meta-analysis

**DOI:** 10.3389/fendo.2026.1832624

**Published:** 2026-07-15

**Authors:** Li Zhan, Lin Wang, Shijie Liu, Sijun Wu, Chicheng Zhou, Zhiji Wang, Fengrui Shi, Youling Qian, Jiewen Xiao, Shuliang Xu

**Affiliations:** 1 School of Physical Education, Wuhan University of Technology, Wuhan, China; 2 Center for Hubei Ethnic Traditional Sports Culture Preservation and Innovation, Wuhan, China; 3 School of Physical Education, Shanghai University of Sport, Shanghai, China; 4 School of Wushu, Wuhan Sports University, Wuhan, China; 5 School of Physical Education, Hubei Minzu University, Enshi, China; 6 School of Music and Physical Education, Hubei Enshi College, Enshi, China

**Keywords:** dose-response relationships, exercise, glycemic control, older adults, physical activity, type 2 diabetes mellitus

## Abstract

**Objective:**

While exercise remains an essential part of managing type 2 diabetes mellitus (T2DM), evidence remains limited regarding which exercise modalities and doses provide the greatest benefit for glycemic control in older adults. Accordingly, we assessed the effects of exercise modality and dose on glycated hemoglobin (HbA1c), fasting blood glucose (FBG), and 2-hour postprandial glucose (2hPG) in older adults with T2DM.

**Methods:**

We searched seven databases from inception to 18 August 2025. Eligible studies were RCTs involving adults with T2DM in which the mean participant age was at least 60 years. Under a Bayesian framework, we conducted a random-effects network meta-analysis combined with dose-response modeling, using MET-min/week as the standardized metric for exercise dose, to assess the effects of exercise on glycemic control in older adults.

**Results:**

Thirty-four RCTs involving 2461 participants were ultimately included. The intervention network comprised continuous aerobic exercise (CAE), combined aerobic and resistance exercise (CE), traditional Chinese sports (TCS), resistance exercise (RE), high-intensity interval training (HIIT), and control group (CG). For HbA1c, CE (MD = −1.05%; 95% CrI: −1.43, −0.64), TCS (MD = −0.80%; 95% CrI: −1.09, −0.52), HIIT (MD = −0.63%; 95% CrI: −1.07, −0.19), CAE (MD = −0.48%; 95% CrI: −0.74, −0.24) and RE (MD = −0.44%; 95% CrI: −0.80, −0.08) showed greater reductions than CG. The SUCRA ranking probabilities favored CE for HbA1c (SUCRA = 94.86%). For FBG, significant reductions versus CG were observed for CE (MD = −1.44 mmol/L; 95% CrI: −2.16, −0.66), CAE (MD = −0.98 mmol/L; 95% CrI: −1.52, −0.41), and TCS (MD = −0.77 mmol/L; 95% CrI: −1.30, −0.23). Ranking probabilities favored CE (SUCRA = 88.03%). In the overall dose-response analyses, significant improvements began at approximately 520 MET-min/week for HbA1c and 500 MET-min/week for FBG. For 2hPG, evidence was limited. Only CE showed a significant reduction versus CG (MD = −3.15 mmol/L; 95% CrI: −5.42, −0.83), and no dose-response analysis was performed because of sparse evidence.

**Conclusions:**

Exercise interventions improved glycemic control in older adults with T2DM, and CE appeared to show a favorable overall profile across glycemic outcomes. Total exercise volume showed nonlinear dose-response relationships with HbA1c and FBG. Further well-designed studies are needed to confirm these dose-response patterns, refine dose ranges for specific exercise modalities, and support more individualized exercise strategies.

**Systematic Review Registration:**

https://www.crd.york.ac.uk/PROSPERO/view/CRD420251127345, identifier CRD420251127345.

## 1 Introduction

The global diabetes burden continues to rise, with a marked shift toward an older age distribution. According to the Global Burden of Disease Study 2019 (GBD 2019), approximately 460 million people were living with diabetes worldwide in 2019, of whom approximately 221 million were aged 60 years or older, accounting for nearly half of all patients with diabetes (1). China has seen a similar trend, with the prevalence of diabetes increasing rapidly among older adults, and over 95% of these cases being type 2 diabetes mellitus (T2DM) (2). Together, these patterns suggest that T2DM in older adults now represents an important public health issue, with population aging likely to further increase its long-term clinical and economic burden (3, 4). Among older adults, T2DM is commonly accompanied by progressive beta-cell dysfunction and increasing insulin resistance (5, 6). If glycemic control remains poor over time, the risks of adverse outcomes, including cardiovascular disease, chronic kidney disease, neuropathy, and cognitive decline, increase substantially (7–9). Therefore, identifying individualized glycemic management strategies for older adults that are safe, effective, and widely applicable has become an urgent priority in both global public health and clinical research.

Exercise interventions are relatively accessible, can be incorporated into routine diabetes management across clinical and community settings, and have been consistently recommended in diabetes guidelines as an important part of T2DM management (10, 11). Existing systematic reviews indicate that various exercise modalities may improve important measures of glycemic control, including glycated hemoglobin (HbA1c), fasting blood glucose (FBG), and 2-hour postprandial glucose (2hPG). Even so, the relative advantages of specific exercise types are still not well defined. Wu (2023) network meta-analysis (NMA) suggested that different mind-body exercises may offer different benefits for HbA1c and FBG (12), whereas earlier comparisons by Schwingshackl (2014) and Pan (2018) of aerobic, resistance, and combined exercise reported inconsistent findings (13, 14). Such differences may be partly explained by variation among the included studies in exercise intensity, frequency, and duration, as well as in accompanying treatments such as medication and dietary management and in baseline population characteristics.

The World Health Organization (WHO) currently recommends 150 to 300 minutes of moderate-intensity aerobic activity per week for older adults, or an equivalent amount of vigorous-intensity activity, as a general target for health promotion (15). For older adults with T2DM, however, it is still unclear which exercise types and dose levels are most closely linked to improvements in glycemic control. Liu (2025) found that combined aerobic and resistance exercise (CE) was associated with relatively stable improvements in FBG, 2hPG, and HbA1c across interventions lasting 14 to 48 weeks (16). However, that study mainly compared exercise modalities according to intervention duration and did not bring intensity, frequency, and duration together into a unified dose measure. While dose-response analyses of exercise and HbA1c have appeared in recent years, they have largely focused on general adult populations (17, 18). Evidence for FBG and 2hPG in older adults with T2DM is still limited, making it harder to formulate exercise recommendations for this group that are both individualized and scalable.

Recent methodological advances have made it possible to extend conventional NMA by combining it with dose-response modeling in a model-based NMA framework (19). This method not only supports simultaneous comparisons across different exercise modalities, but also helps show how treatment effects vary with dose, offering a more practical basis for exercise strategies. In this study, we applied NMA to examine how different exercise modalities affected HbA1c, FBG, and 2hPG in older adults with T2DM. We then used a common MET-min/week metric to model dose-response relationships, with the aim of exploring potential nonlinear patterns, identifying the minimum effective dose, and estimating the dose range most likely to confer benefit. Overall, the study provides evidence that may be useful for improving exercise planning strategies for older adults with T2DM.

## 2 Methods

### 2.1 Registration

This review was reported in accordance with the PRISMA extension for NMA (20). The study protocol was prospectively registered in PROSPERO (CRD420251127345). As the analysis was based entirely on data from previously published studies, neither ethics approval nor informed consent was required.

### 2.2 Search strategy

We searched seven major databases in both English and Chinese, including PubMed, Web of Science, Embase, the Cochrane Library, China National Knowledge Infrastructure (CNKI), Wanfang Database, and VIP Chinese Science, from database inception to 18 August 2025. The search strategy combined subject headings with free-text terms. Key concepts included type 2 diabetes, non-insulin-dependent diabetes, exercise, glycemic control, and randomized controlled trials. The full search strategies for each database are provided in **Supplementary Appendix 2**.

### 2.3 Eligibility criteria

Inclusion criteria: adults with T2DM whose mean age was 60 years or older, regardless of sex, race, or nationality; interventions involving one of five exercise modalities: continuous aerobic exercise (CAE), resistance exercise (RE), high-intensity interval training (HIIT), combined aerobic and resistance exercise (CE), or traditional Chinese sports (TCS); and an intervention duration of at least 4 weeks, given that both initial adaptation and sustained physiological responses to exercise typically require time to emerge (18). Control groups could receive any non-exercise comparator, such as usual care. Studies also had to report at least one primary outcome, including FBG, HbA1c, or 2hPG, and use a randomized controlled trial (RCT) design.

Exclusion criteria: studies that were non-randomized, duplicate publications, did not involve patients with T2DM, were animal experiments or reviews, lacked a clearly defined exercise intervention, or did not provide accessible full-text articles or sufficient data for analysis.

### 2.4 Study selection and data extraction

Two reviewers (ZW and CZ) independently carried out study selection and data extraction on the basis of the predefined eligibility criteria, and their decisions were checked against each other. After importing all records into reference management software and removing duplicates, titles and abstracts were screened for preliminary eligibility. We next examined the full texts of studies considered potentially eligible and finalized the included studies on that basis. Disagreements were resolved by discussion and, when required, by a third reviewer (LZ).

Data from eligible studies were recorded using a predefined extraction form. The extracted data included the first author, country, year of publication, participant characteristics (sample size, age, sex distribution, BMI, and duration of diabetes), intervention details (exercise modality, total intervention period, session frequency, session length, and medication or dietary management when available), methodological information related to trial analysis and implementation (analysis population, intervention adherence or compliance), and outcome data. When relevant information was missing or insufficiently reported, we contacted the original authors for clarification or additional data.

### 2.5 Data coding and management

Exercise dose was measured in MET-min/week in this study. It was derived from the product of the MET value for each activity, session duration, and weekly frequency, and was used to estimate total weekly exercise volume. MET values were assigned according to the 2024 Older Adult Compendium of Physical Activities, which provides activity-specific MET60+ values for adults aged 60 years and older and covers a broad range of exercise modalities relevant to older adults (21). Weekly frequency was defined as the total number of exercise sessions, including multiple sessions completed on the same day. When session duration increased over the course of the intervention, the average duration across the full intervention period was used. For network connectivity and dose network modeling, exercise dose was further grouped into seven categories: 0 (control), 250, 500, 750, 1000, 1250, and 1500 MET-min/week (18).

### 2.6 Risk of bias assessment

The risk of bias of the included RCTs was independently examined by two reviewers with the Revised Cochrane risk-of-bias tool for randomized trials (RoB 2) (22). Five aspects were considered, including the randomization process, deviations from intended interventions, missing outcome data, outcome measurement, and selective reporting. An overall judgment was generated for each study on the basis of these assessments, and ratings for each domain were recorded as low risk, some concerns, or high risk.

### 2.7 Credibility assessment

Evidence certainty for each intervention comparison was evaluated with CINeMA (Confidence in Network Meta-Analysis) (23), a web-based tool developed to extend GRADE methodology to NMA. In accordance with current guidance, judgments were made across six domains: within-study bias, reporting bias, indirectness, imprecision, heterogeneity, and incoherence. The final certainty rating for each comparison was categorized as high, moderate, low, or very low.

### 2.8 Statistical analysis

#### 2.8.1 Pairwise meta-analysis

Treatment effects were estimated using changes from baseline calculated from the pre-intervention and post-intervention means and standard deviations (24). When at least four studies were available for a specific exercise modality, a pairwise meta-analysis was performed (25). The pooled effects were reported as mean differences (MDs) with 95% credible intervals (CrIs). Between-study heterogeneity was evaluated using the I² statistic, with values above 50% considered indicative of substantial heterogeneity (26). Possible small-study effects and publication bias were evaluated by visual inspection of comparison adjusted funnel plots and by Egger’s regression test (27).

#### 2.8.2 NMA

For the outcomes, treatment effects were expressed as MDs, preferentially using change from baseline data (28). In line with the PRISMA extension for NMA (29), direct and indirect evidence were synthesized in a random-effects NMA conducted in R with the gemtc package. Network plots were used to display the evidence structure, with node size and edge thickness reflecting sample size and study number, respectively. To assess the plausibility of the transitivity assumption, we examined the distribution of available potential effect modifiers across intervention nodes, as reported in **Supplementary Appendix 6.5**. Model estimation was based on Markov chain Monte Carlo methods (30). Four chains with different initial values were run for 25,000 iterations, and the first 5,000 were discarded as burn-in. Global inconsistency was examined by comparing the consistency model with the unrelated mean effects (UME) model, and a small deviance information criterion (DIC) difference together with residual deviance contributions and the heterogeneity parameter was interpreted as indicating no important inconsistency. Local inconsistency was assessed by node splitting, with two-sided p values below 0.05 indicating inconsistency (31, 32). Posterior ranking probabilities (surface under the cumulative ranking, SUCRA) were calculated for each outcome (33), and because lower HbA1c, FBG, and 2hPG values indicate greater benefit, the ranking direction was specified accordingly. Forest plots and league tables were used to present relative effects and uncertainty (34).

#### 2.8.3 Dose–response network meta-analysis

All analyses were performed in R (version 4.5.1) with the MBNMAdose package, and the dose-response plots were generated using ggplot2. A Bayesian random effects MBNMA was applied to examine how exercise dose was associated with glycemic outcomes (35). Before model fitting, we assessed the key assumptions required for the analysis, including network connectivity, consistency, and transitivity, as described in **Supplementary Appendix 7.1** (36). We first explored the observed overall effects of exercise on glycemic control and then examined the patterns across exercise modalities and dose levels. On this basis, several nonlinear functions were compared, including the non-monotonic, restricted cubic spline, Emax, and quadratic models (37). Model fit was judged using the deviance information criterion (DIC), standard deviation (SD), the effective number of parameters (pD), and residual distributions. Among the candidate nonlinear models, the quadratic random-effects model provided the best overall fit for both HbA1c and FBG, mainly as indicated by the lowest DIC values. Additional model fit statistics, including pD and residual deviance, are provided in **Supplementary Table S13**. It was therefore selected for the final dose-response analyses. The predicted effects with their corresponding 95% CrIs were used to identify the lowest dose associated with statistically significant improvement and to estimate the effective dose ranges for each exercise modality (38). In the dose-response analysis, statistical significance was defined as a 95% CrI that did not cross 0.

#### 2.8.4 Additional analyses

We further performed network meta-regression to explore whether study- and participant-level characteristics influenced the intervention effects at different exercise dose levels. The continuous covariates included age, BMI, proportion of male participants, intervention duration (weeks), exercise frequency, publication year and sample size. Sensitivity analyses were also carried out by excluding studies with a high risk of bias and trials lasting less than 12 weeks to assess the robustness of the estimated effects.

## 3 Results

### 3.1 Literature search results

**Figure 1:**
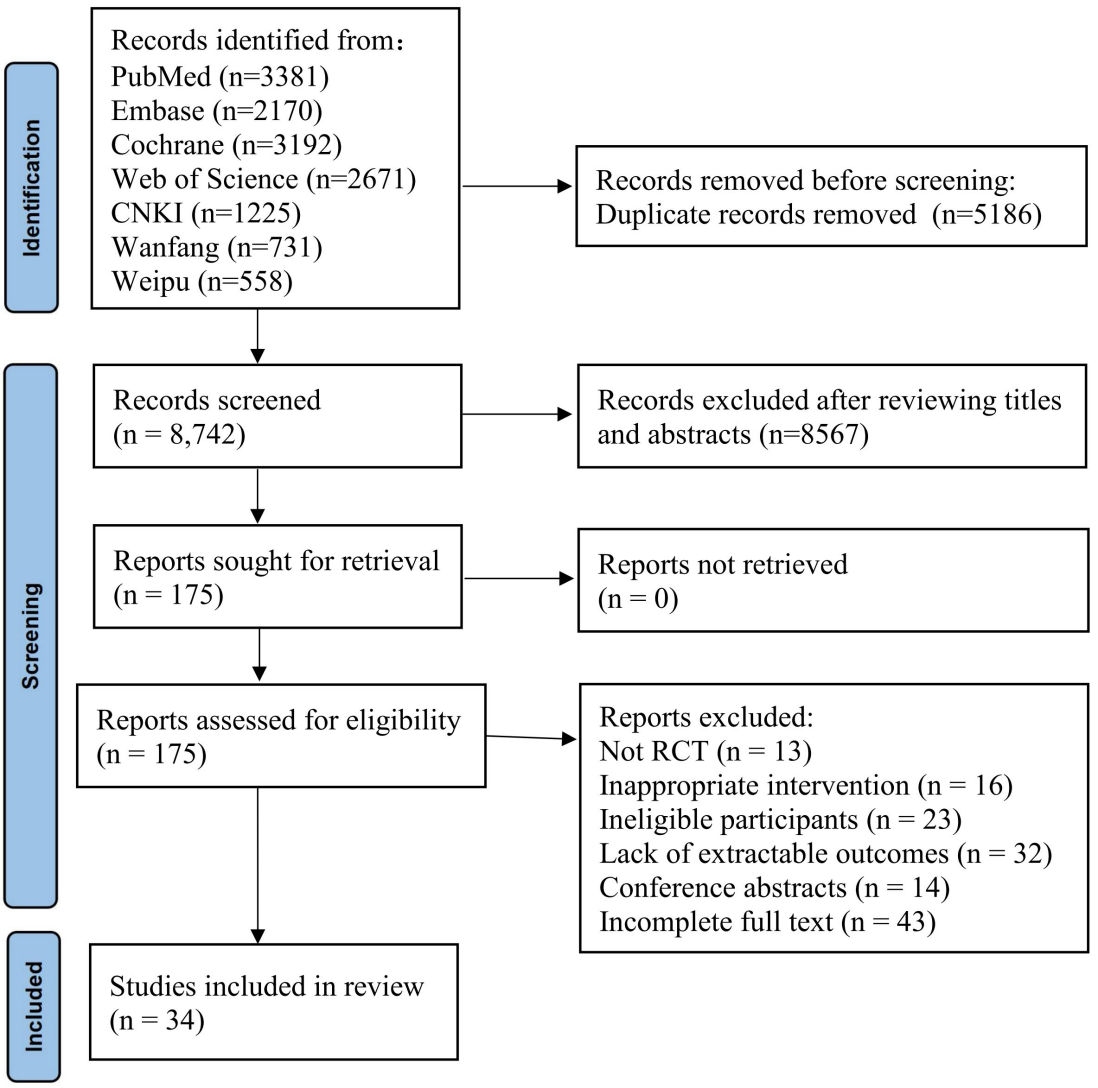
Flow diagram.

The database search identified 13,928 records. After duplicate records were removed, 8,742 remained for title and abstract screening. A total of 175 articles were then assessed in full text for eligibility. Ultimately, 34 randomized controlled trials were included (**Figure 1**). Of these, 32 studies contributed to the NMA for HbA1c, 25 for FBG, and 8 for 2hPG.

### 3.2 Basic characteristics of included studies

The 34 included RCTs comprised 2461 participants, and the mean age in all studies was at least 60 years. Five exercise categories were represented: CAE (17 studies), CE (7 studies), TCS (9 studies), RE (6 studies), and HIIT (5 studies). Across studies, exercise was performed 2 to 7 days per week, with 1 to 2 sessions per day, and the average session duration was about 46 minutes (**Supplementary Appendix 3**).

### 3.3 Risk of bias and evidence assessment

Of the 34 included studies, 4 were assessed as having high risk, 24 were judged as having some concerns, and 6 were considered to have low risk. Detailed results for both individual domains and overall judgments are provided in **Supplementary Appendix 4**. According to CINeMA, the network comparisons were supported by evidence of very low to moderate certainty. A summary of these certainty ratings is available in **Supplementary Appendix 6.8**.

### 3.4 Pairwise meta-analyses

Pairwise analyses showed that, relative to the control group, CAE, CE, and TCS significantly improved HbA1c, FBG, and 2hPG, whereas RE was not associated with significant changes in these outcomes. Evaluation of small study effects suggested no evidence of publication bias for HbA1c based on funnel plot inspection and Egger’s test (p = 0.2701). By contrast, possible publication bias was observed for FBG (p = 0.0337), which should be considered when interpreting this outcome. Publication bias was not evaluated for 2hPG because no comparison included more than three studies (**Supplementary Appendix 5.2**).

### 3.5 NMA

#### 3.5.1 HbA1c

**Figure 2:**
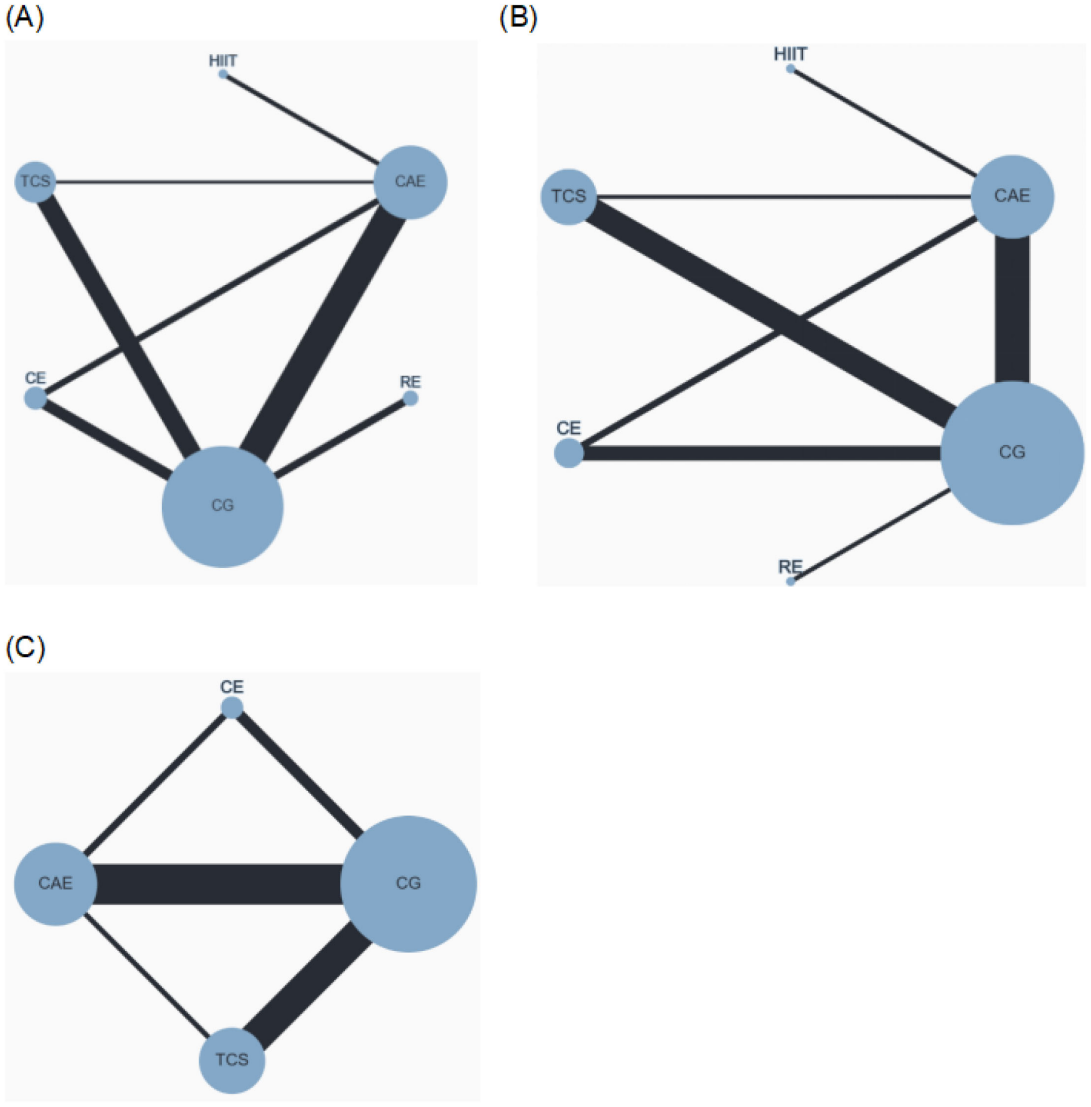
Network plots of evidence for glycemic outcomes across different exercise interventions. The network plots illustrate the direct and indirect comparisons included in the network meta-analysis. The size of each node is proportional to the sample size for that intervention, and the thickness of each connecting line is proportional to the number of studies contributing to the corresponding direct comparison. **(A)** HbA1c; **(B)** FBG; **(C)** 2hPG.

**Figure 3:**
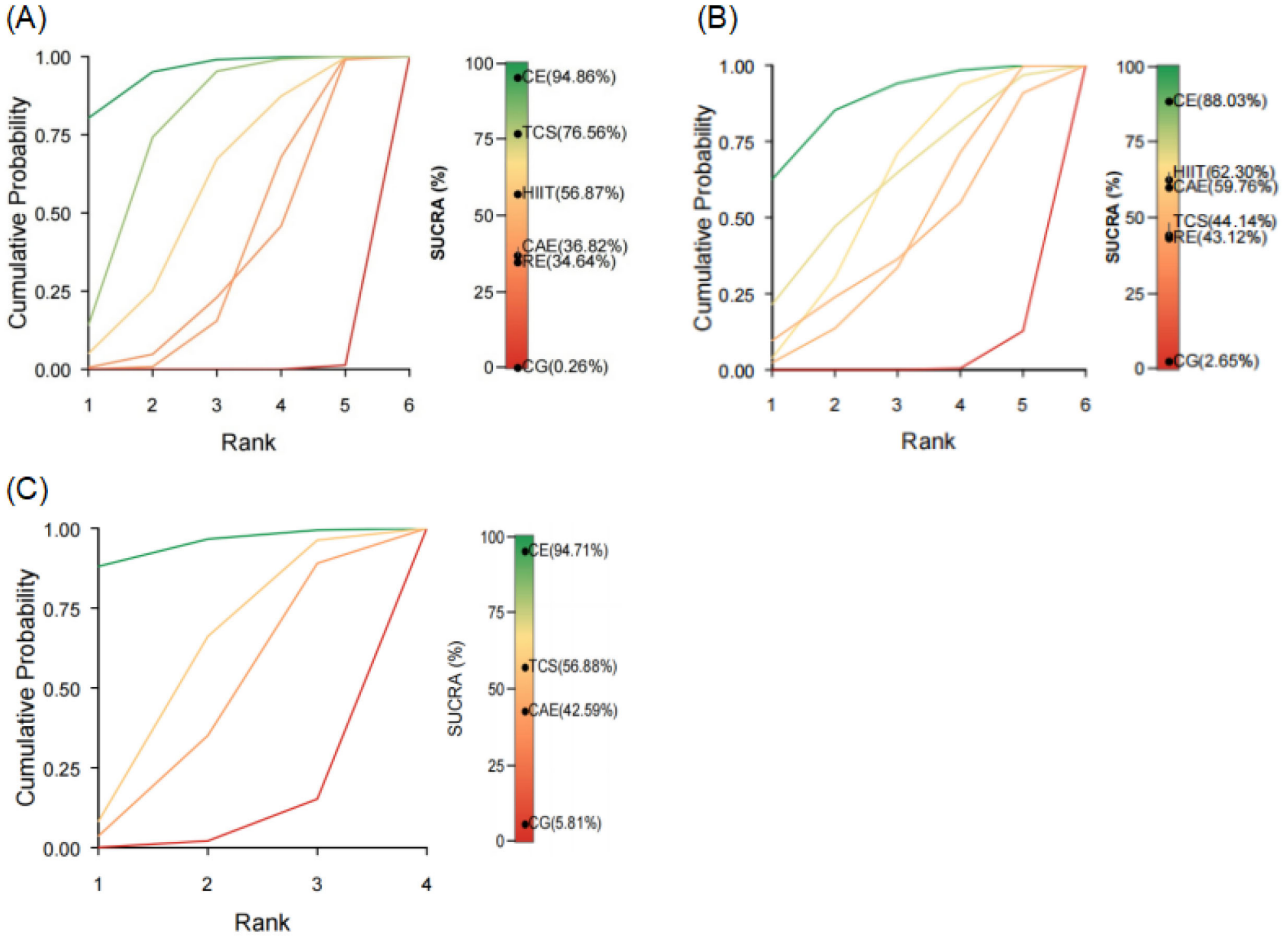
Cumulative ranking probability plots for glycemic outcomes across different exercise interventions. Cumulative ranking probabilities and SUCRA values were calculated from the network meta-analysis. Higher SUCRA values indicate a greater probability of ranking among the more effective options, with curves closer to the upper left corner indicating more favorable rankings. **(A)** HbA1c; **(B)** FBG; **(C)** 2hPG.

For the HbA1c analysis, 32 RCTs comprising 2280 participants were included. The corresponding network structure is presented in **Figure 2A**. Compared with the control group, CE, TCS, HIIT, CAE and RE were associated with reductions in HbA1c. The estimated MDs were −1.05% (95% CrI: −1.43, −0.64) for CE, −0.80% (95% CrI: −1.09, −0.52) for TCS, −0.63% (95% CrI: −1.07, −0.19) for HIIT, −0.48% (95% CrI: −0.74, −0.24) for CAE and −0.44% (95% CrI: −0.80, −0.08) for RE (**Table 1**). The ranking probabilities were generally consistent with these effect estimates, with CE showing the highest SUCRA value (94.86%), followed by TCS (76.56%), HIIT (56.87%), CAE (36.82%), and RE (34.64%) (**Figure 3A**). The consistency and UME models showed comparable fit for HbA1c (DIC 121.2 vs. 120.0; ΔDIC = 1.2), with no indication of global inconsistency. Node-splitting analyses also suggested no local inconsistency (p > 0.05).

**Table 1:** Comparison of the effects of different exercise interventions on glycemic control outcomes.

Outcome	Comparison					
HbA1c	CAE					
	0.56(0.13, 0.98)	CE				
	0.14(-0.24, 0.51)	-0.41(-0.97, 0.15)	HIIT			
	-0.05(-0.50, 0.40)	-0.61(-1.13, -0.05)	-0.19(-0.76, 0.40)	RE		
	0.32(-0.02, 0.65)	-0.24(-0.71, 0.25)	0.17(-0.33, 0.68)	0.36(-0.11, 0.84)	TCS	
	**-0.48(-0.74, -0.24)**	**-1.05(-1.43, -0.64)**	**-0.63(-1.07, -0.19)**	**-0.44(-0.80, -0.08)**	**-0.80(-1.09, -0.52)**	CG
FBG	CAE					
	0.47(-0.39, 1.26)	CE				
	0.06(-0.90, 1.00)	-0.40(-1.66, 0.87)	HIIT			
	-0.27(-1.45, 0.98)	-0.74(-2.00, 0.65)	-0.33(-1.83, 1.25)	RE		
	-0.21(-0.88, 0.48)	-0.67(-1.54, 0.25)	-0.27(-1.43, 0.92)	0.07(-1.17, 1.25)	TCS	
	**-0.98(-1.52, -0.41)**	**-1.44(-2.16, -0.66)**	-1.03(-2.12, 0.09)	-0.70(-1.81, 0.35)	**-0.77(-1.30, -0.23)**	CG
2hPG	CAE					
	2.08(-0.67, 4.75)	CE				
	0.37(-1.96, 2.71)	-1.71(-4.53, 1.15)	TCS			
	-1.07(-3.12, 0.99)	**-3.15(-5.42, -0.83)**	-1.44(-3.28, 0.39)	CG		

The estimates in this table are derived from the full evidence network and should not be interpreted as direct pairwise estimates alone.

Bold values indicate statistically significant effects, defined as effects for which the 95% CrI did not include 0.

#### 3.5.2 FBG

For the FBG outcome, 25 trials involving 1955 participants were included. The network structure is shown in **Figure 2B**. **Table 1** presents the relative effects across interventions. Compared with the control group, CE (MD = −1.44 mmol/L; 95% CrI: −2.16, −0.66), CAE (MD = −0.98 mmol/L; 95% CrI: −1.52, −0.41), and TCS (MD = −0.77 mmol/L; 95% CrI: −1.30, −0.23) significantly reduced FBG. In contrast, HIIT (MD = −1.03 mmol/L; 95% CrI: −2.12, 0.09) and RE (MD = −0.70 mmol/L; 95% CrI: −1.81, 0.35) did not differ significantly from the control group. In the SUCRA analysis, CE had the highest ranking probability (88.03%), followed by HIIT (62.30%), CAE (59.76%), TCS (44.14%), and RE (43.12%), whereas the control group had the lowest ranking probability (2.65%) (**Figure 3B**).

Model fit showed only a small difference between the consistency model (DIC = 96.1) and the UME model (DIC = 93.8; ΔDIC = 2.3), suggesting no evident global inconsistency. At the local level, node-splitting analysis identified inconsistency in the CAE–CE comparison (p = 0.0335). We therefore performed a sensitivity analysis to examine its influence on the findings. After excluding studies judged to be at high risk of bias, the inconsistency was no longer observed (**Supplementary Appendix 6**).

#### 3.5.3 2hPG

For the 2hPG outcome, 8 trials involving 1001 participants were included. **Table 1** summarizes the relative effects of the interventions. Among the evaluated exercise modalities, only CE was associated with a significant reduction in 2hPG relative to the control group (MD = −3.15 mmol/L; 95% CrI: −5.42, −0.83). Neither CAE nor TCS differed significantly from the control group, suggesting considerable uncertainty in the effect estimates. In the SUCRA analysis, CE had the highest ranking probability (94.71%), followed by TCS (56.88%) and CAE (42.59%), whereas the control group had the lowest ranking probability (5.81%) (**Figure 3C**). Given the limited number of studies contributing to this outcome and the uncertainty around several comparisons, the 2hPG findings should be interpreted cautiously. Model fit showed only a minimal difference between the consistency model (DIC = 32.6) and the UME model (DIC = 32.5; ΔDIC = 0.1), indicating no evidence of important global inconsistency. Node-splitting analysis likewise did not detect significant local inconsistency.

### 3.6 Dose–response meta-analysis

**Figure 4:**
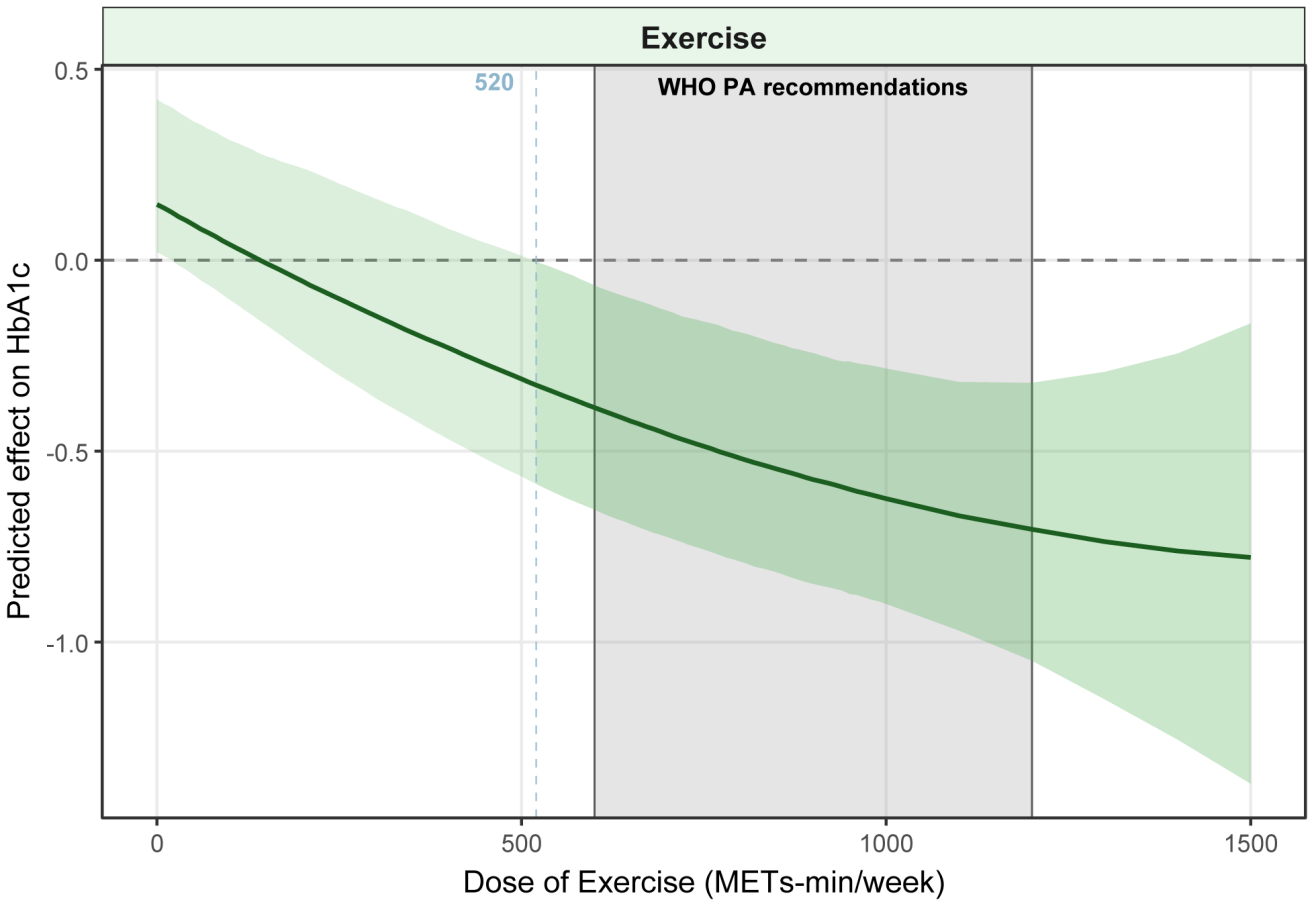
Dose-response relationship between total exercise volume and HbA1c in older adults with T2DM. Negative MD values indicate greater reductions in HbA1c and therefore favor exercise. The green shaded area represents the dose range in which the 95% credible interval did not cross zero. Blue numbers indicate the lower and upper boundaries of the statistically significant dose range.

**Figure 5:**
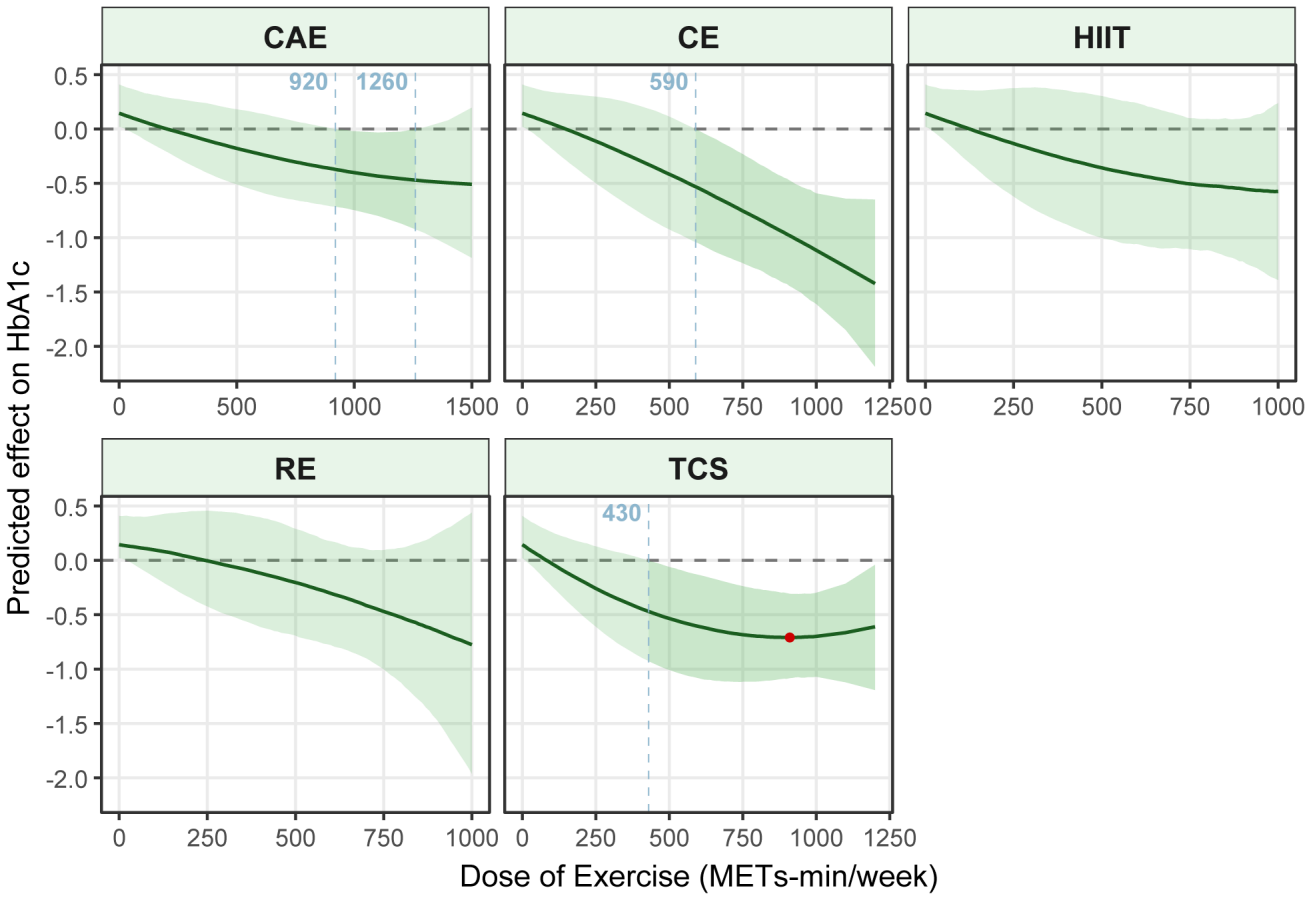
Dose-response relationships between exercise modalities and HbA1c in older adults with T2DM. Negative MD values indicate greater reductions in HbA1c and therefore favor exercise. The green shaded areas indicate dose ranges in which the 95% credible intervals did not cross zero, and the blue numbers represent the lower and upper boundaries of these significant dose ranges. Red dots indicate the model-estimated dose points with the largest predicted reductions within the evaluated dose range. CAE, continuous aerobic exercise; CE, combined aerobic and resistance exercise; RE, resistance exercise; HIIT, high-intensity interval training; TCS, traditional Chinese sports.

A nonlinear relationship was found between total exercise dose and HbA1c (**Figure 4**). According to the model, HbA1c showed a significant decline when the exercise dose reached approximately 520 MET-min/week (MD = -0.33%; SD = 0.15). Beyond about 1400 MET-min/week, additional reductions became minimal (slope = 0.012/100 MET-min/week). At 600 MET-min/week, the predicted reduction in HbA1c was -0.39% (95% CrI: -0.66, -0.06; SD = 0.15). At 1200 MET-min/week, the predicted reduction was -0.69% (95% CrI: -1.05, -0.32; SD = 0.19). The dose-response relationship and the corresponding model-estimated MET-min/week ranges for each specific exercise modality are shown in **Figure 5** and **Supplementary Appendix 7.3.1**, respectively. In the ranking analysis across dose levels, the probabilities for HbA1c favored CE at approximately 1200 MET-min/week among the evaluated exercise modalities and dose levels (**Supplementary Appendix 7.3.2**). In the *post hoc* sensitivity analysis excluding HIIT arms, the overall HbA1c dose-response curve remained broadly similar to the main analysis, showing a progressive reduction with increasing exercise dose followed by a tendency to plateau at higher doses (**Supplementary Figure S19**).

**Figure 6:**
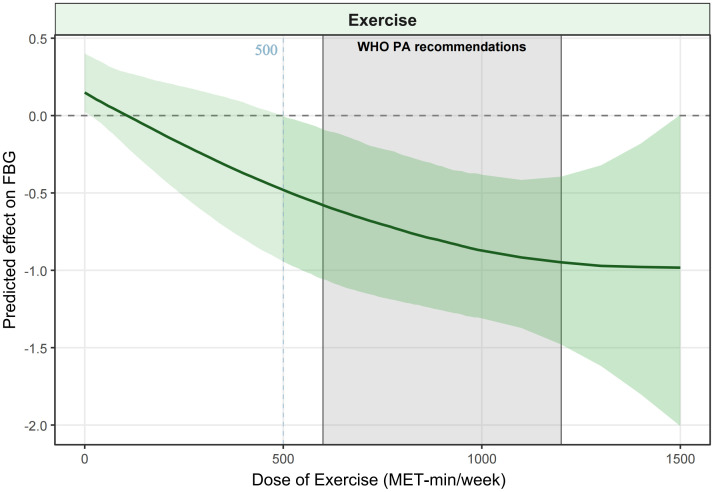
Dose-response relationship between total exercise volume and FBG in older adults with T2DM. Negative MD values indicate greater reductions in FBG and therefore favor exercise. The green shaded area represents the dose range in which the 95% credible interval did not cross zero. Blue numbers indicate the lower and upper boundaries of the statistically significant dose range.

**Figure 7:**
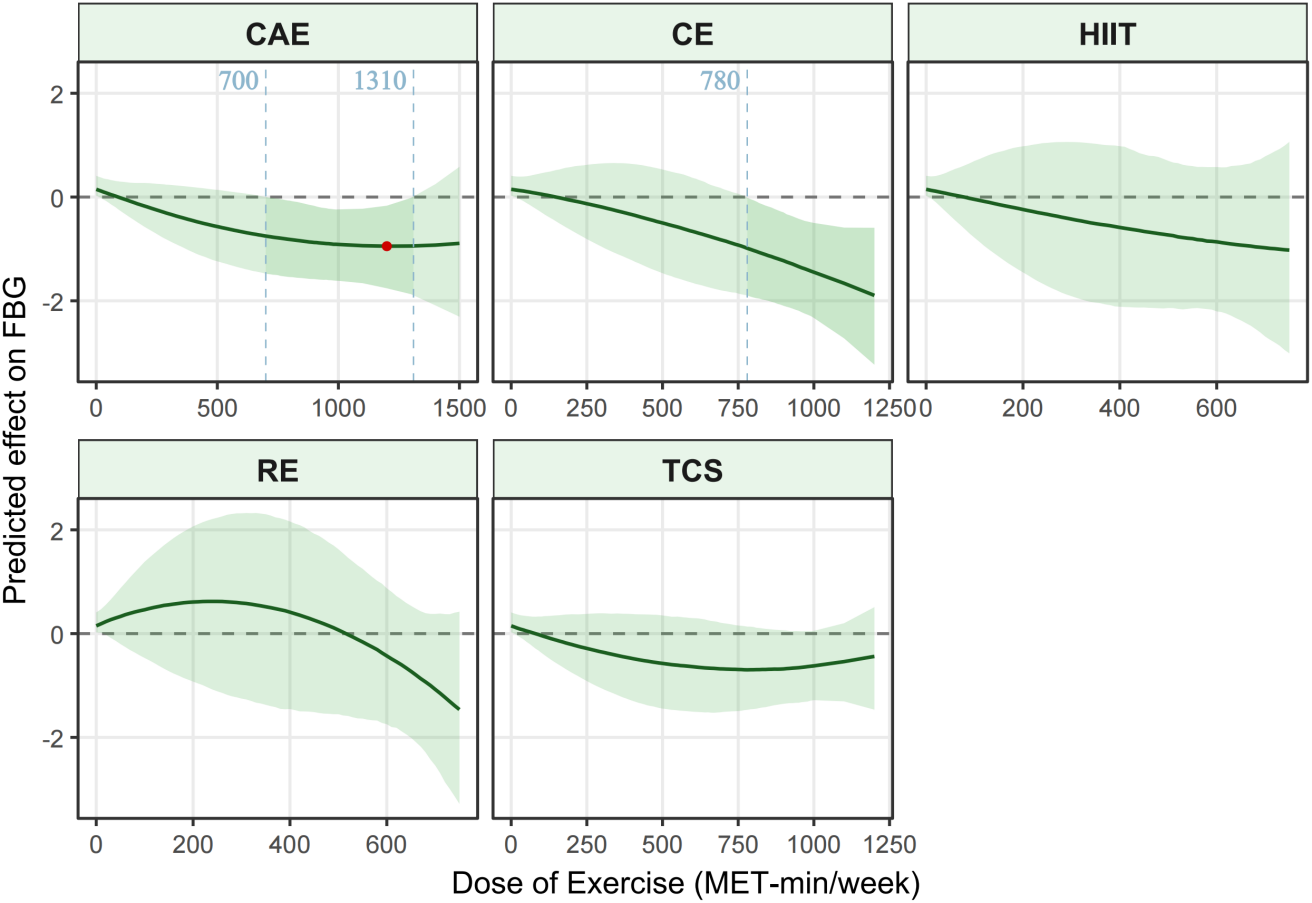
Dose-response relationships between exercise modalities and FBG in older adults with T2DM. Negative MD values indicate greater reductions in FBG and therefore favor exercise. The green shaded areas indicate dose ranges in which the 95% credible intervals did not cross zero, and the blue numbers represent the lower and upper boundaries of these significant dose ranges. Red dots indicate the model-estimated dose points with the largest predicted reductions within the evaluated dose range. CAE, continuous aerobic exercise; CE, combined aerobic and resistance exercise; RE, resistance exercise; HIIT, high-intensity interval training; TCS, traditional Chinese sports.

Total exercise dose was inversely associated with FBG in a nonlinear manner (**Figure 6**). According to the model, a statistically significant improvement in FBG emerged at an exercise dose of about 500 MET-min/week (MD = −0.48 mmol/L; SD = 0.24). Once the dose exceeded roughly 1300 MET-min/week, the additional benefit became limited (slope = 0.0103/100 MET-min/week). At 600 MET-min/week, the estimated effect was MD = −0.58 mmol/L (95% CrI: −1.06, −0.09; SD = 0.25). At 1200 MET-min/week, the estimated effect was MD = −0.95 mmol/L (95% CrI: −1.48, −0.39; SD = 0.28). The dose-response relationship and the corresponding estimated MET-min/week ranges for each specific exercise modality are shown in **Figure 7** and **Supplementary Appendix 7.3.1**, respectively. A similar pattern was observed for FBG, with CE at approximately 1200 MET-min/week receiving the most favorable ranking probability among the evaluated exercise modalities and dose levels (**Supplementary Appendix 7.3.2**).

Because only eight RCTs reported 2hPG and stratified data were sparse, the parameters of the dose-response models could not be estimated reliably. Therefore, no dose-response analysis was performed for 2hPG.

### 3.7 Additional analyses

Network meta-regression and sensitivity analyses did not identify any factors that materially affected the main findings, supporting the robustness of the results (**Supplementary Appendix 6**).

## 4 Discussion

### 4.1 Principal findings

This review included RCTs involving older adults with T2DM. Using MET-min/week as a unified dose metric, we evaluated glycemic changes according to exercise modality and dose-response patterns. Overall, most exercise interventions improved HbA1c and FBG compared with usual care. Within the current network of randomized controlled evidence, CE showed a relatively consistent beneficial profile across the evaluated glycemic outcomes. Dose-response analyses showed nonlinear associations between total exercise dose and both HbA1c and FBG. HbA1c began to improve at approximately 520 MET-min/week, with the effect gradually plateauing beyond about 1400 MET-min/week. FBG began to improve at approximately 500 MET-min/week, with diminishing returns emerging at around 1300 MET-min/week. Because the number of studies reporting 2hPG was limited, the current evidence remains insufficient to support a robust dose-response inference for this outcome.

### 4.2 Effects of different exercise modalities and possible underlying mechanisms

The present study suggests that CE may offer a favorable overall glycemic profile across HbA1c, FBG, and 2hPG. This overall pattern is in line with the findings reported in the Bayesian NMA by Liu (2025) (16). The clinical relevance of CE may also extend beyond glycemic control. Zhang (2024) reported that CE improved cognition, metabolic health, physical function, and physical health-related quality of life in older adults with T2DM (39). These broader benefits are relevant to older adults with T2DM, who frequently present with sarcopenia or sarcopenic obesity, impaired physical function, and chronic low-grade inflammation. In this setting, better insulin sensitivity and greater skeletal muscle glucose uptake are likely to result from the interaction of multiple physiological mechanisms rather than from any single pathway (40, 41). Because HbA1c reflects average glycemic exposure over the past 2 to 3 months, changes in this outcome may be more indicative of sustained metabolic adaptation and a lower long-term glycemic burden after exercise intervention (42). CE includes both aerobic and resistance components. The aerobic component may improve insulin sensitivity and reduce adiposity-related metabolic stress. Resistance exercise may help preserve muscle mass and strength in older adults and increase skeletal muscle responsiveness to insulin and contraction-related stimulation (43). Relative to single-modality exercise, this combination is more likely to produce cumulative improvements in metabolism and body composition, which may contribute to the larger reduction observed in HbA1c (43–45).

FBG reflects endogenous hepatic glucose production and hepatic insulin resistance in the fasting state. It may therefore be responsive to training-related changes in the balance between hepatic glucose output and peripheral glucose utilization (46, 47). Aerobic exercise may improve insulin sensitivity in both the liver and skeletal muscle. It may also partly suppress hepatic glucose output during fasting (48). This pathway may be especially relevant in older adults, who often present with fatty liver disease and abnormal fat distribution. Resistance exercise, by enhancing skeletal muscle glucose uptake and utilization efficiency, may help reduce reliance on hepatic glucose output during fasting (3, 43, 45, 49). At the same time, improvements in muscle strength may support daily physical activity and long-term adherence. Taken together, the combination of these two types of stimuli may be more conducive to lowering and stabilizing fasting blood glucose.

2hPG reflects postprandial glycemic responses. In addition to overall insulin sensitivity, it depends strongly on the immediate capacity of skeletal muscle to dispose of glucose (47, 49, 50). In CE, the resistance component provides a stronger contractile stimulus, which may promote GLUT4 translocation through insulin-independent pathways and thereby enhance immediate postprandial glucose uptake (50–52). The aerobic component, in turn, may improve skeletal muscle microvascular function and perfusion responses, thereby increasing the efficiency of postprandial glucose delivery and utilization (43, 53). However, postprandial glucose is relatively sensitive to exercise timing, and differences across trials in exercise scheduling and measurement conditions may introduce additional heterogeneity (54, 55).

### 4.3 Dose-response relationship analysis

We found a nonlinear association between total weekly exercise dose and HbA1c. A significant reduction in HbA1c emerged at around 520 MET-min/week, but the magnitude of further improvement was small beyond approximately 1400 MET-min/week. This overall pattern is consistent with the direction of recent dose-response evidence. For example, Liang (2024) similarly reported in adults with T2DM that HbA1c decreased with increasing exercise dose, with a significant response emerging at approximately 840 MET-min/week and the curve gradually plateauing near 1300 MET-min/week (18). Benham (2020), examining the association between exercise and HbA1c from the perspectives of actual completed dose and adherence, suggested that a dose-response relationship was mainly observed for CAE and CE, but did not identify a clear dose threshold that could be directly applied in clinical exercise strategies (56). Gallardo-Gómez (2024) also reported a nonlinear relationship, proposing that approximately 1100 MET-min/week may be close to the optimal dose and noting that this association was influenced by baseline HbA1c levels (17). One possible explanation for the lower minimum significant dose observed in this study is variation across studies in participant age structure, inclusion criteria, and modeling methods.

Unlike HbA1c, which reflects long-term integrated glycemic exposure, FBG is more akin to a short-term state indicator and is readily influenced by recent diet, sleep, stress, medication use, and the interval between the last exercise session and blood sampling, with some degree of within-person variability (42, 57, 58). Therefore, the dose-response pattern for FBG may capture not only longer-term physiological adaptation, but also short-term changes in insulin sensitivity related to recent exercise exposure. In the present study, FBG decreased nonlinearly with increasing exercise dose, with significant effects emerging at approximately 500 MET-min/week and the incremental benefit gradually diminishing beyond about 1300 MET-min/week. Nevertheless, the available evidence on the dose-response relationship for FBG is still sparse. Most existing studies have focused on dose exploration within a single training modality or across different training types, suggesting that dose components such as frequency, intensity, and duration may influence FBG, but these findings cannot yet be directly translated into weekly total dose ranges for different exercise modalities (59–61).

Further analyses by exercise modality suggested that dose-response patterns may differ across training types. These differences may be related to the characteristics of each modality, such as the combined aerobic and resistance components in CE, the need for regular weekly accumulation in CAE, and the lower-intensity but more sustainable nature of TCS (62–65). However, these modality-specific patterns should be interpreted with caution because several curves were based on a limited number of studies. Study-level factors, including baseline glycemic status, diabetes duration, comorbidity burden, adherence, medication or dietary management, and differences between prescribed and actually achieved training dose, may also have contributed to the observed heterogeneity (66–68). This issue may be particularly relevant for FBG, which is more susceptible to short-term changes in diet, sleep, stress, and medication use (69).

Taken together, the dose-response findings should be interpreted mainly from the overall curves, which are less likely to be influenced by any single study. The modality-specific curves may help identify possible differences across exercise types, but they remain exploratory, especially when based on a limited number of studies. Apparent nonlinear patterns in these analyses should not be viewed as definitive dose-response relationships. Future trials with larger samples and better reporting of adherence, safety, medication and dietary management, and achieved exercise dose are needed to clarify modality-specific dose-response patterns in older adults with T2DM.

### 4.4 Clinical implications for diabetes management

These findings may provide a practical reference for exercise planning in older adults with T2DM. The dose-response results suggested that HbA1c and FBG may respond at somewhat different weekly exercise volumes, indicating that exercise advice should be linked to the main glycemic target rather than applying a single dose strategy to all glucose-related outcomes. **Table 2** presents the estimated dose ranges and illustrative weekly exercise patterns for different glycemic outcomes.

**Table 2:** Estimated exercise dose thresholds and illustrative weekly patterns for glycemic outcomes.

Outcome	Type of exercise	Estimated effective dose threshold (MET-min/week)	Intensity	MET value	Estimated weekly exercise duration (min/week)	Illustrative weekly exercise patterns (sessions/week × min/session)
HbA1c	CAE	920	Moderate	4.3 (code 0101160)	~214	5 × ~ 45 min; 6 × ~ 40 min
			Vigorous	6.3 (code 0101260)	~146	3 × ~ 30 min; 4 × ~ 25 min
	CE	590	Moderate	4.3 (mean of codes 0205460, 0101160)	~138	5 × ~ 30 min; 6 × ~ 25 min
			Vigorous	7.6 (mean of codes 0207360, 0101260)	~78	3 × ~ 30 min; 4 × ~ 20 min
	TCS	430	Moderate	3.8 (code 0221060)	~114	5 × ~ 25 min; 6 × ~ 20 min
FBG	CAE	700	Moderate	4.3 (code 0101160)	~163	5 × ~ 35 min; 6 × ~ 30 min
			Vigorous	6.3 (code 0101260)	~112	3 × ~ 40 min; 4 × ~ 30 min
	CE	780	Moderate	4.3 (mean of codes 0205460, 0101160)	~182	5 × ~ 40 min; 6 × ~ 35 min
			Vigorous	7.6 (mean of codes 0207360, 0101260)	~103	3 × ~ 35 min; 4 × ~ 30 min

The MET-min/week values represent exercise dose thresholds estimated from the dose-response network meta-analysis, rather than fixed clinical prescriptions. Weekly exercise durations were calculated by dividing the estimated dose threshold by the corresponding MET value and are provided to facilitate interpretation. The weekly exercise patterns are illustrative examples only and should be adapted according to baseline fitness, comorbidities, medication use, safety considerations, tolerance, supervision, and adherence. CAE, continuous aerobic exercise; CE, combined aerobic and resistance exercise; TCS, traditional Chinese sports.

Although the certainty of evidence was limited for some modality-specific comparisons, this mainly affects the interpretation of exact effect sizes, rankings, and dose ranges rather than the broader clinical value of structured exercise. The lower ratings were largely related to imprecision, heterogeneity, and methodological limitations of the included trials. Precise modality rankings and dose ranges should be interpreted cautiously and used as references for individualized exercise planning, while regular physical activity should still be recognized as an important part of diabetes management.

In terms of exercise modality, CE showed a relatively consistent profile across HbA1c, FBG, and 2hPG, suggesting that CE may be useful when improvement across several glycemic indicators is desired. TCS also showed favorable effects on HbA1c and may be easier to deliver in community or home-based settings because it requires little equipment and fewer specific facilities. For some older adults, particularly those who have difficulty maintaining higher-intensity or highly structured exercise programs, more sustainable activities such as TCS or walking may be a reasonable option if they support regular participation.

In practice, the HbA1c reductions observed for several exercise modalities reached or exceeded approximately 0.4 percentage points, suggesting that these effects may be clinically relevant. However, in older adults with T2DM, the practical value of exercise should not be judged by a single glycemic outcome alone, but also in relation to the target glycemic outcome, baseline glycemic status, comorbidities, medication use, physical function, safety, feasibility, tolerance, supervision, adherence, and patient preference. Follow-up should include assessment of glycemic response and adverse events, especially hypoglycemia, falls, cardiovascular symptoms, and reasons for exercise interruption.

## 5 Strengths and limitations

A major strength of this study is that pairwise meta-analysis, NMA, and dose-response analysis were integrated within the same analytical framework. This allowed us to compare how different exercise modalities and dose levels were associated with glycemic outcomes in a more systematic way. It also provided quantitative evidence to support comparisons across intervention types and to identify potentially relevant dose ranges. To test the robustness of the findings, we carried out sensitivity analyses after removing studies with a high risk of bias and those with shorter intervention periods. We also used meta-regression to examine whether several key variables influenced the intervention effects, which helped inform the interpretation of the results across different study settings.

At the same time, several limitations need to be considered. First, the certainty of evidence ranged from very low to moderate, with lower certainty mainly observed in some modality-specific comparisons. Possible publication bias was also detected for FBG based on Egger’s test. These issues should be taken into account when interpreting the strength and robustness of the evidence. Second, medication use, dietary management, analysis population, and intervention adherence were incompletely or inconsistently reported across trials. These factors may influence glycemic response and the dose-response relationship, but their effects could not be fully accounted for. Changes within the control groups may also have attenuated or amplified the estimated relative effects and should be considered when interpreting weak or null findings. Third, although MET-min/week provides a practical common scale for exercise dose, it may not fully capture modality-specific features of RE, such as load, sets, repetitions, and progression, or HIIT features, such as peak intensity and work-to-rest structure. This limitation may be particularly relevant in older adults, whose physiological responses to the same MET-based dose can vary by fitness level, muscle mass, and comorbidities. Finally, some dose response associations for individual exercise modalities did not reach statistical significance, which may reflect the small number of studies and limited dose coverage rather than a true absence of effect. Accordingly, the estimated dose thresholds should be interpreted as approximate reference ranges rather than precise individualized prescriptions, and future studies should report modality-specific dose components more completely.

## 6 Conclusions

By combining NMA with dose-response analysis, this study examined the associations of exercise modality and exercise dose with glycemic control in older adults with T2DM. The results suggest that CE may provide the most consistent overall benefit across HbA1c, FBG, and 2hPG among the exercise types included in this review. We also found nonlinear inverse associations between total exercise dose and both HbA1c and FBG, with significant improvement appearing at about 520 MET-min/week for HbA1c and about 500 MET-min/week for FBG. These findings may be useful for shaping more targeted exercise strategies for older adults with T2DM and may also provide a basis for future studies examining optimal exercise patterns, appropriate dose ranges, and the mechanisms through which exercise improves glycemic control.

## Data Availability

The original contributions presented in the study are included in the article/**Supplementary Material**. Further inquiries can be directed to the corresponding author.
