# Supplementary material for: The optimal exercise modality and dose for glycemic control in older adults with type 2 diabetes mellitus: a systematic review and network meta-analysis
Source: Front Endocrinol (Lausanne). 2026 Jul 15;17:1832624. doi: 10.3389/fendo.2026.1832624 (PMC13414825; doi:10.3389/fendo.2026.1832624)
Supplement: Supplementary file 1 [file DataSheet1.pdf]

Supplementary Material

The optimal exercise modality and dose for glycemic control in older adults with type 2 diabetes mellitus: a systematic review and network meta-analysis

Supplementary Appendix

Contents

1. PRISMA-NMA Checklist 3

2. Search strategy 10

3. Demographic characteristics of included studies 17

4. Risk of bias 26

5. Pairwise meta-analyses and publication bias 26

5.1 Details of pairwise meta-analyses 26

5.2 Publication bias 27

6. Bayesian network meta-analysis 28

6.1 Model fit summaries for included studies 28

6.2 Node-split for all studies 28

6.3 Forest plot of all studies 29

6.4 SUCRA table for all studies 30

6.5 Assessment of transitivity assumption 30

6.6 Sensitivity analysis 34

6.6.1 Model fit summaries for all studies and sensitivity analyses 34

6.6.2 Node-split for sensitivity analyses 36

6.6.3 SUCRA of sensitivity analyses 37

6.6.4 Forest plot and league table for sensitivity analyses 38

6.7 Network meta-regression 40

6.8 Summary grading of evidence 41

7. Dose-response network meta-analyses 44

7.1 Key assumptions for network meta-analysis 44

7.1.1 Network connectivity 44

7.1.2 Data consistency 46

7.1.3 Network transitivity 47

7.2 Models selection 52

7.2.1 Nonlinear functions and model-fit comparison 52

7.2.2 Models selection 55

7.3 Dose-response relationships 61

7.3.1 Dose-response relationship between exercise dose and glycemic control 61

7.3.2 Effectiveness ranking of different exercise modalities 68

1. PRISMA-NMA Checklist

Section/Topic	Item #	Checklist Item	Reported on Page #
**TITLE**			
Title	1	Identify the report as a systematic review *incorporating a network meta-analysis (or related form of meta-analysis).*	1
**ABSTRACT**			
Structured summary	2	Provide a structured summary including, as applicable:<br>**Background:** main objectives<br>**Methods:** data sources; study eligibility criteria, participants, and interventions; study appraisal; and *synthesis methods, such as network meta-analysis.*<br>**Results:** number of studies and participants identified; summary estimates with corresponding confidence/credible intervals; *treatment rankings may also be discussed. Authors may choose to summarize pairwise comparisons against a chosen treatment included in their analyses for brevity.*<br>**Discussion/Conclusions:** limitations; conclusions and implications of findings.<br>**Other:** primary source of funding; systematic review registration number with registry name.	1-2
**INTRODUCTION**			
Rationale	3	Describe the rationale for the review in the context of what is already known,	2

		including mention of why a network meta-analysis has been conducted.	
Objectives	4	Provide an explicit statement of questions being addressed, with reference to participants, interventions, comparisons, outcomes, and study design (PICOS).	2
**METHODS**			
Protocol and registration	5	Indicate whether a review protocol exists and if and where it can be accessed (e.g., Web address); and, if available, provide registration information, including registration number.	3
Eligibility criteria	6	Specify study characteristics (e.g., PICOS, length of follow-up) and report characteristics (e.g., years considered, language, publication status) used as criteria for eligibility, giving rationale. *Clearly describe eligible treatments included in the treatment network, and note whether any have been clustered or merged into the same node (with justification).*	3
Information sources	7	Describe all information sources (e.g., databases with dates of coverage, contact with study authors to identify additional studies) in the search and date last searched.	3
Search	8	Present full electronic search strategy for at least one database, including any limits used, such that it could be repeated.	Supplementary Appendix 2: 10–16
Study selection	9	State the process for selecting studies (i.e., screening, eligibility, included in systematic review, and, if applicable, included in the meta-analysis).	3
Data collection process	10	Describe method of data extraction from reports (e.g., piloted forms, independently, in duplicate) and any processes for	3

		obtaining and confirming data from investigators.	
Data items	11	List and define all variables for which data were sought (e.g., PICOS, funding sources) and any assumptions and simplifications made.	3
**Geometry of the network**	**S1**	Describe methods used to explore the geometry of the treatment network under study and potential biases related to it. This should include how the evidence base has been graphically summarized for presentation, and what characteristics were compiled and used to describe the evidence base to readers.	4
Risk of bias within individual studies	12	Describe methods used for assessing risk of bias of individual studies (including specification of whether this was done at the study or outcome level), and how this information is to be used in any data synthesis.	3
Summary measures	13	State the principal summary measures (e.g., risk ratio, difference in means). *Also describe the use of additional summary measures assessed, such as treatment rankings and surface under the cumulative ranking curve (SUCRA) values, as well as modified approaches used to present summary findings from meta-analyses.*	4
Planned methods of analysis	14	Describe the methods of handling data and combining results of studies for each network meta-analysis. This should include, but not be limited to:<br>• *Handling of multi-arm trials;*<br>• *Selection of variance structure;*<br>• *Selection of prior distributions in Bayesian analyses; and*<br>• *Assessment of model fit.*	3-4

**Assessment of Inconsistency**	**S2**	Describe the statistical methods used to evaluate the agreement of direct and indirect evidence in the treatment network(s) studied. Describe efforts taken to address its presence when found.	4
Risk of bias across studies	15	Specify any assessment of risk of bias that may affect the cumulative evidence (e.g., publication bias, selective reporting within studies).	4
Additional analyses	16	Describe methods of additional analyses if done, indicating which were pre-specified. This may include, but not be limited to, the following:<br>• Sensitivity or subgroup analyses;<br>• Meta-regression analyses;<br>• *Alternative formulations of the treatment network; and*<br>• *Use of alternative prior distributions for Bayesian analyses (if applicable).*	4
**RESULTS†**			
Study selection	17	Give numbers of studies screened, assessed for eligibility, and included in the review, with reasons for exclusions at each stage, ideally with a flow diagram.	4-5
**Presentation of network structure**	**S3**	Provide a network graph of the included studies to enable visualization of the geometry of the treatment network.	6

**Summary of network geometry**	**S4**	Provide a brief overview of characteristics of the treatment network. This may include commentary on the abundance of trials and randomized patients for the different interventions and pairwise comparisons in the network, gaps of evidence in the treatment network, and potential biases reflected by the network structure.	4-6
Study characteristics	18	For each study, present characteristics for which data were extracted (e.g., study size, PICOS, follow-up period) and provide the citations.	4; Supplementary Appendix 3: 17–25
Risk of bias within studies	19	Present data on risk of bias of each study and, if available, any outcome level assessment.	4; Supplementary Appendix 4: 26
Results of individual studies	20	For all outcomes considered (benefits or harms), present, for each study: 1) simple summary data for each intervention group, and 2) effect estimates and confidence intervals. *Modified approaches may be needed to deal with information from larger networks.*	4–7; Supplementary Appendix 3: 17–25; Supplementary Appendix 5.1: 26–27
Synthesis of results	21	Present results of each meta-analysis done, including confidence/credible intervals. *In larger networks, authors may focus on comparisons versus a particular comparator (e.g. placebo or standard care), with full findings presented in an appendix. League tables and forest plots may be considered to summarize pairwise comparisons.* If additional summary measures were explored (such as treatment rankings), these should also be presented.	4-9
**Exploration for inconsistency**	**S5**	Describe results from investigations of inconsistency. This may include such information as measures of model fit to compare consistency and inconsistency models, *P* values from statistical tests, or summary of inconsistency estimates from different parts of the treatment network.	5–6; Supplementary Appendix 6.2: 28

Risk of bias across studies	22	Present results of any assessment of risk of bias across studies for the evidence base being studied.	4–5; Supplementary Appendix 5.2: 27
Results of additional analyses	23	Give results of additional analyses, if done (e.g., sensitivity or subgroup analyses, meta-regression analyses*, alternative network geometries studied, alternative choice of prior distributions for Bayesian analyses,* and so forth).	7, 10; Supplementary Appendix 6.6–6.7: 35–40
**DISCUSSION**			
Summary of evidence	24	Summarize the main findings, including the strength of evidence for each main outcome; consider their relevance to key groups (e.g., healthcare providers, users, and policy-makers).	10-11
Limitations	25	Discuss limitations at study and outcome level (e.g., risk of bias), and at review level (e.g., incomplete retrieval of identified research, reporting bias). *Comment on the validity of the assumptions, such as transitivity and consistency. Comment on any concerns regarding network geometry (e.g., avoidance of certain comparisons).*	11-12
Conclusions	26	Provide a general interpretation of the results in the context of other evidence, and implications for future research.	12
**FUNDING**			
Funding	27	Describe sources of funding for the systematic review and other support (e.g., supply of data); role of funders for the systematic review. This should also include information regarding whether funding has been received from manufacturers of treatments in the network	13

		and/or whether some of the authors are content experts with professional conflicts of interest that could affect use of treatments in the network.	

2. Search strategy

Cochrane Library

#1 (Diabetes Mellitus, Type 2 OR Diabetes Mellitus, Stable OR Stable Diabetes Mellitus OR Diabetes Mellitus, Noninsulin Dependent OR Diabetes Mellitus, Adult-Onset OR Adult-Onset Diabetes Mellitus OR Diabetes Mellitus, Adult Onset OR Diabetes Mellitus, Ketosis-Resistant OR Diabetes Mellitus, Ketosis Resistant OR Ketosis-Resistant Diabetes Mellitus OR Diabetes Mellitus, Non Insulin Dependent OR Diabetes Mellitus, Non-Insulin-Dependent OR Non-Insulin-Dependent Diabetes Mellitus OR Diabetes Mellitus, Type II OR NIDDM OR Diabetes Mellitus, Maturity-Onset OR Diabetes Mellitus, Maturity Onset OR Maturity-Onset Diabetes Mellitus OR Maturity Onset Diabetes Mellitus OR Type 2 Diabetes Mellitus OR Noninsulin-Dependent Diabetes Mellitus OR Noninsulin Dependent Diabetes Mellitus OR Maturity-Onset Diabetes OR Diabetes, Maturity-Onset OR Maturity Onset Diabetes OR Type 2 Diabetes OR Diabetes, Type 2 OR Diabetes Mellitus, Noninsulin-Dependent):ti,ab,kw

#2 (randomized controlled trial* OR controlled clinical trial OR RCT OR random* OR clinical trial* OR randomly OR trial OR clinical trial OR randomized controlled trial* OR cross-over):ti,ab,kw

#3 (Resistance training OR Training, Resistance OR Strength Training OR Training, Strength OR Weight-Lifting Strengthening Program OR Strengthening Program,Weight-Lifting OR Strengthening Programs,Weight-Lifting OR Weight Lifting Strengthening Program OR Weight Lifting Strengthening Programs OR Weight-Lifting Exercise Program OR Exercise Program,Weight-Lifting OR Exercise Programs, Weight-Lifting OR Weight Lifting Exercise Program OR Weight-Lifting Exercise Programs OR Weight-Bearing Strengthening Program OR Strengthening Program, Weight-Bearing OR Strengthening Programs, Weight-Bearing OR Weight Bearing Strengthening Program OR Weight-Bearing Strengthening Programs OR Weight-Bearing Exercise Program):ti,ab,kw

#4 (mind-body exercises OR yoga OR tai ji OR tai chi OR chi, tai OR tai ji quan OR ji quan, tai OR quan, tai ji OR taiji OR taijiquan OR tai chi chuan OR qigong OR qi gong OR pilates training OR training, pilates OR pilates OR baduanjin OR ba duan jin OR eight section brocades OR eight trigrams boxing OR eight-treasured exercises OR eight pieces of brocade OR eight brocade section OR eight-section brocade OR five-animal exercises OR five animal exercise OR five animal frolics OR movements of five animals OR five mimic-animal boxing OR wuqinxi OR wu qin xi OR liuzijue OR liu zi jue OR six-character formula OR six healing sounds OR yijinjing OR yi jin jing OR muscle-bone strengthening exercise OR the classics of tendon changing OR mind-body therap* OR pilates OR dance OR dancing OR dances OR ballet OR jazz dance OR dance jazz OR tap dance OR dance, tap OR modern dance OR dance, modern OR hip-hop dance OR dance, hip-hop OR hip hop dance OR line dancing OR dancing, line OR salsa dancing OR dancing, salsa OR square

dance OR dance, square OR pilates-based exercises OR exercises, pilates-based OR pilates based exercises OR pilates training):ti,ab,kw
#5 (aerobic exercise OR aerobic training OR multidisciplinary exercise program OR nordic walking OR running OR treadmill training OR walking):ti,ab,kw
#6 (High Intensity Interval Training OR HIIT OR High-Intensity Interval Trainings OR Interval Training,High-Intensity OR Training,High-Intensity Interval OR Trainings, High-Intensity Interval OR High-Intensity Intermittent Exercise OR Exercise, High-Intensity Intermittent OR Exercises, High-Intensity Intermittent OR High-Intensity Intermittent Exercises OR Sprint Interval Training OR Sprint Interval Trainings):ti,ab,kw
#7 (Exercise* OR Physical Activity OR Activities, Physical OR Activity, Physical OR Physical Activities OR Exercise, Physical OR Exercises, Physical OR Physical Exercise OR Physical Exercises OR Exercise, Isometric OR Exercises, Isometric OR Isometric Exercises OR Isometric Exercise OR Exercise Training OR Exercise Trainings OR Training, Exercise OR Trainings, Exercise):ti,ab,kw
#8 #3 OR #4 OR #5 OR #6 OR #7
#9 #1 AND #2 AND #8

Embase
#1 'diabetes mellitus, type 2':ab,ti
#2 ('diabetes mellitus, stable' OR 'stable diabetes mellitus' OR 'diabetes mellitus, noninsulin dependent' OR 'diabetes mellitus, adult-onset' OR 'adult-onset diabetes mellitus' OR 'diabetes mellitus, adult onset' OR 'diabetes mellitus, ketosis-resistant' OR 'diabetes mellitus, ketosis resistant' OR 'ketosis-resistant diabetes mellitus' OR 'diabetes mellitus, non insulin dependent' OR 'diabetes mellitus, non-insulin-dependent' OR 'non-insulin-dependent diabetes mellitus' OR 'diabetes mellitus, type ii' OR 'niddm' OR 'diabetes mellitus, maturity-onset' OR 'diabetes mellitus, maturity onset' OR 'maturity-onset diabetes mellitus' OR 'maturity onset diabetes mellitus' OR 'type 2 diabetes mellitus' OR 'noninsulin-dependent diabetes mellitus' OR 'noninsulin dependent diabetes mellitus' OR 'maturity-onset diabetes' OR 'diabetes, maturity-onset' OR 'maturity onset diabetes' OR 'type 2 diabetes' OR 'diabetes, type 2' OR 'diabetes mellitus, noninsulin-dependent'):ab,ti
#3 ('resistance training' OR 'training, resistance' OR 'strength training' OR 'training, strength' OR 'weight-lifting strengthening program' OR 'strengthening program,weight-lifting' OR 'strengthening programs,weight-lifting' OR 'weight lifting strengthening program' OR 'weight lifting strengthening programs' OR 'weight-lifting exercise program' OR 'exercise program,weight-lifting' OR 'exercise programs, weight-lifting' OR 'weight lifting exercise program' OR 'weight-lifting exercise programs' OR 'weight-bearing strengthening program' OR 'strengthening program, weight-bearing' OR 'strengthening programs, weight-bearing' OR 'weight bearing strengthening program' OR 'weight-bearing strengthening programs' OR 'weight-bearing exercise program'):ab,ti

#4 ('mind-body exercises' OR 'yoga' OR 'tai ji' OR 'tai-ji' OR 'tai chi' OR 'chi, tai' OR 'tai ji quan' OR 'ji quan, tai' OR 'quan, tai ji' OR 'taiji' OR 'taijiquan' OR 'tai chi chuan' OR 'qigong' OR 'qi gong' OR 'pilates training' OR 'training, pilates' OR 'pilates' OR 'baduanjin' OR 'ba duan jin' OR 'eight section brocades' OR 'eight trigrams boxing' OR 'eight-treasured exercises' OR 'eight pieces of brocade' OR 'eight brocade section' OR 'eight-section brocade' OR 'five-animal exercises' OR 'five animal exercise' OR 'five animal frolics' OR 'movements of five animals' OR 'five mimic-animal boxing' OR 'wuqinxi' OR 'wu qin xi' OR 'liuzijue' OR 'liu zi jue' OR 'six-character formula' OR 'six healing sounds' OR yijinjing OR 'yi jin jing' OR 'muscle-bone strengthening exercise' OR 'the classics of tendon changing' OR 'mind-body therap*' OR 'pilates' OR 'dance' OR 'dancing' OR 'dances' OR 'ballet' OR 'jazz dance' OR 'dance, jazz' OR 'tap dance' OR 'dance, tap' OR 'modern dance' OR 'dance, modern' OR 'hip-hop dance' OR 'dance, hip-hop' OR 'hip hop dance' OR 'line dancing' OR 'dancing, line' OR 'salsa dancing' OR 'dancing, salsa' OR 'square dance' OR 'dance, square' OR 'pilates-based exercises' OR 'exercises, pilates-based' OR 'pilates based exercises' OR 'pilates training'):ab,ti
#5 ('aerobic exercise' OR 'aerobic training' OR 'multidisciplinary exercise program' OR 'nordic walking' OR 'running' OR 'treadmill training' OR 'walking'):ab,ti
#6 ('high intensity interval training' OR 'high-intensity interval trainings' OR 'interval training,high-intensity' OR 'training,high-intensity interval' OR 'trainings, high-intensity interval' OR 'hiit' OR 'high-intensity intermittent exercise' OR 'exercise, high-intensity intermittent' OR 'exercises, high-intensity intermittent 'OR 'high-intensity intermittent exercises' OR 'sprint interval training' OR 'sprint interval trainings'):ab,ti
#7 ('exercise*' OR 'physical activity' OR 'activities,physical' OR 'activity, physical' OR 'physical activities' OR 'exercise,physical' OR 'exercises, physical' OR 'physical exercise' OR 'physical exercises' OR 'exercise, isometric' OR 'exercises, isometric' OR 'isometric exercises' OR 'isometric exercise' OR 'exercise training' OR 'exercise trainings' OR 'training, exercise' OR 'trainings, exercise'):ab,ti
#8 ('randomized controlled trial*' OR 'controlled clinical trial' OR 'random*' OR 'clinical trial*'OR 'randomly' OR 'trial' OR 'clinical trial' OR 'randomized controlled trial*' OR "cross-over" OR RCT):ab,ti
#9 #1 OR #2
#10 #3 OR #4 OR #5 OR #6 OR #7
#11 #8 AND #9 AND #10

Web of Science
#1 TS=("Diabetes Mellitus, Type 2")
#2 TS=("Diabetes Mellitus, Stable" OR "Stable Diabetes Mellitus" OR "Diabetes Mellitus, Noninsulin Dependent" OR "Diabetes Mellitus, Adult-Onset" OR "Adult-Onset Diabetes Mellitus" OR "Diabetes Mellitus, Adult Onset" OR "Diabetes Mellitus, Ketosis-Resistant" OR "Diabetes Mellitus, Ketosis Resistant" OR "Ketosis-

Resistant Diabetes Mellitus" OR "Diabetes Mellitus, Non Insulin Dependent" OR "Diabetes Mellitus, Non-Insulin-Dependent" OR "Non-Insulin-Dependent Diabetes Mellitus" OR "Diabetes Mellitus, Type II" OR "NIDDM" OR "Diabetes Mellitus, Maturity-Onset" OR "Diabetes Mellitus, Maturity Onset" OR "Maturity-Onset Diabetes Mellitus" OR "Maturity Onset Diabetes Mellitus" OR "Type 2 Diabetes Mellitus" OR "Noninsulin-Dependent Diabetes Mellitus" OR "Noninsulin Dependent Diabetes Mellitus" OR "Maturity-Onset Diabetes" OR "Diabetes, Maturity-Onset" OR "Maturity Onset Diabetes" OR "Type 2 Diabetes" OR "Diabetes, Type 2" OR "Diabetes Mellitus, Noninsulin-Dependent" OR "T2DM")
#3 TS=("Resistance training" OR "Training, Resistance" OR "Strength Training" OR "Training, Strength" OR "Weight-Lifting Strengthening Program" OR "Strengthening Program,Weight-Lifting" OR "Strengthening Programs,Weight-Lifting" OR "Weight Lifting Strengthening Program" OR "Weight Lifting Strengthening Programs" OR "Weight-Lifting Exercise Program" OR "Exercise Program,Weight-Lifting" OR "Exercise Programs, Weight-Lifting" OR "Weight Lifting Exercise Program" OR "Weight-Lifting Exercise Programs" OR "Weight-Bearing Strengthening Program" OR "Strengthening Program, Weight-Bearing" OR "Strengthening Programs, Weight-Bearing" OR "Weight Bearing Strengthening Program" OR "Weight-Bearing Strengthening Programs" OR "Weight-Bearing Exercise Program")
#4 TS=("mind-body exercises" OR "Yoga" OR "Tai Ji" OR "Taiji" OR "Tai Chi" OR "Chi, Tai" OR "Tai Ji Quan" OR "Ji Quan, Tai" OR "Quan, Tai Ji" OR "Taijiquan" OR "T'ai Chi" OR "Tai Chi Chuan" OR "Qigong" OR "Qi Gong" OR "Ch'i Kung" OR "Pilates-Based Exercises" OR "Exercises, Pilates-Based" OR "Pilates Based Exercises" OR "Pilates Training" OR "Training, Pilates" OR "baduanjin" OR "Pilates" OR "baduanjin" OR "ba duan jin" OR "eight section brocades" OR "eight trigrams boxing" OR "eight-treasured exercises" OR "eight pieces of brocade" OR" eight brocade section" OR "eight-section brocade" OR "Five-animal exercises" OR "Five Animal Exercise" OR "Five animal Frolics" OR "movements of five animals" OR "Five mimic-animal boxing" OR "Wuqinxi" OR "wu qin xi" OR "Liuzijue" OR "liu zi jue" OR "six-character formula" OR "Six Healing Sounds" OR Yijinjing OR "yi jin jing" OR "muscle-bone strengthening exercise" OR "the classics of tendon changing" OR "mind-body therap*" OR "Pilates" OR "dance" OR "dancing" OR "dances" OR "Ballet" OR "Jazz Dance" OR "Dance, Jazz" OR "Tap Dance" OR "Dance, Tap" OR "Modern Dance" OR "Dance, Modern" OR "Hip-Hop Dance" OR "Dance, Hip-Hop" OR "Hip Hop Dance" OR "Line Dancing" OR "Dancing, Line" OR "Salsa Dancing" OR "Dancing, Salsa" OR "Square Dance" OR "Dance, Square" OR "Pilates-Based Exercises" OR "Exercises, Pilates-Based" OR "Pilates Based Exercises" OR "Pilates Training")
#5 TS=("aerobic exercise" OR "aerobic training" OR "multidisciplinary exercise program" OR "Nordic Walking" OR "running" OR "treadmill training" OR "walking")
#6 TS=("High Intensity Interval Training" OR "High-Intensity Interval Trainings" OR "Interval Training,High-Intensity" OR "HIIT" OR "Training,High-Intensity Interval" OR "Trainings, High-Intensity Interval" OR "High-Intensity Intermittent Exercise"

OR "Exercise, High-Intensity Intermittent" OR "Exercises, High-Intensity Intermittent" OR "High-Intensity Intermittent Exercises" OR "Sprint Interval Training" OR "Sprint Interval Trainings")
#7 TS=("Exercise*" OR "Physical Activity" OR "Activities,Physical" OR "Activity, Physical" OR "Physical Activities" OR "Exercise,Physical" OR "Exercises, Physical" OR "Physical Exercise" OR "Physical Exercises" OR "Exercise, Isometric" OR "Exercises, Isometric" OR "Isometric Exercises" OR "Isometric Exercise" OR "Exercise Training" OR "Exercise Trainings" OR "Training, Exercise" OR "Trainings, Exercise")
#8 TS=("randomized controlled trial*" OR "controlled clinical trial" OR "random*" OR "RCT" OR "clinical trial*"OR "randomly" OR "trial" OR "clinical trial" OR "randomized controlled trial*" OR "cross-over")
#9 #1 OR #2
#10 #3 OR #4 OR #5 OR #6 OR #7
#11 #8 AND #9 AND #10

PubMed
#1 "Diabetes Mellitus, Type 2"[MeSH]
#2 "Diabetes Mellitus, Stable"[Title/Abstract] OR "type 2 diabetic"[Title/Abstract] OR "Stable Diabetes Mellitus"[Title/Abstract] OR "Diabetes Mellitus, Noninsulin Dependent"[Title/Abstract] OR "Diabetes Mellitus, Adult-Onset"[Title/Abstract] OR "Adult-Onset Diabetes Mellitus"[Title/Abstract] OR "Diabetes Mellitus, Adult Onset"[Title/Abstract] OR "Diabetes Mellitus, Ketosis-Resistant"[Title/Abstract] OR "Diabetes Mellitus, Ketosis Resistant"[Title/Abstract] OR "Ketosis-Resistant Diabetes Mellitus"[Title/Abstract] OR "Diabetes Mellitus, Non Insulin Dependent"[Title/Abstract] OR "Diabetes Mellitus, Non-Insulin-Dependent"[Title/Abstract] OR "Non-Insulin-Dependent Diabetes Mellitus"[Title/Abstract] OR "Diabetes Mellitus, Type II"[Title/Abstract] OR "NIDDM"[Title/Abstract] OR "Diabetes Mellitus, Maturity-Onset"[Title/Abstract] OR "Diabetes Mellitus, Maturity Onset"[Title/Abstract] OR "Maturity-Onset Diabetes Mellitus"[Title/Abstract] OR "Maturity Onset Diabetes Mellitus"[Title/Abstract] OR "Type 2 Diabetes Mellitus"[Title/Abstract] OR "Noninsulin-Dependent Diabetes Mellitus"[Title/Abstract] OR "Noninsulin Dependent Diabetes Mellitus"[Title/Abstract] OR "Maturity-Onset Diabetes"[Title/Abstract] OR "Diabetes, Maturity-Onset"[Title/Abstract] OR "Maturity Onset Diabetes"[Title/Abstract] OR "Type 2 Diabetes"[Title/Abstract] OR "Diabetes, Type 2"[Title/Abstract] OR "Diabetes Mellitus, Noninsulin-Dependent"[Title/Abstract] OR "T2DM"[Title/Abstract]
#3 "Resistance training"[Title/Abstract] OR "Training, Resistance"[Title/Abstract] OR "Strength Training"[Title/Abstract] OR "Training, Strength"[Title/Abstract] OR "Weight-Lifting Strengthening Program"[Title/Abstract] OR "Strengthening Program,Weight-Lifting"[Title/Abstract] OR "Strengthening Programs,Weight-Lifting"[Title/Abstract] OR "Weight Lifting Strengthening Program"[Title/Abstract] OR "Weight Lifting Strengthening Programs"[Title/Abstract] OR "Weight-Lifting

Exercise Program"[Title/Abstract] OR "Exercise Program,Weight-Lifting"[Title/Abstract] OR "Exercise Programs, Weight-Lifting"[Title/Abstract] OR "Weight Lifting Exercise Program"[Title/Abstract] OR "Weight-Lifting Exercise Programs"[Title/Abstract] OR "Weight-Bearing Strengthening Program"[Title/Abstract] OR "Strengthening Program, Weight-Bearing"[Title/Abstract] OR "Strengthening Programs, Weight-Bearing"[Title/Abstract] OR "Weight Bearing Strengthening Program"[Title/Abstract] OR "Weight-Bearing Strengthening Programs"[Title/Abstract] OR "Weight-Bearing Exercise Program"[Title/Abstract]
#4 "mind-body exercises"[Title/Abstract] OR "Yoga"[Title/Abstract] OR "Tai Ji"[Title/Abstract] OR "Tai-ji"[Title/Abstract] OR "Tai Chi"[Title/Abstract] OR "Chi, Tai"[Title/Abstract] OR "Tai Ji Quan"[Title/Abstract] OR "Ji Quan, Tai"[Title/Abstract] OR "Quan, Tai Ji"[Title/Abstract] OR "Taiji"[Title/Abstract] OR "Taijiquan"[Title/Abstract] OR "T'ai Chi"[Title/Abstract] OR "Tai Chi Chuan"[Title/Abstract] OR "Qigong"[Title/Abstract] OR "Qi Gong"[Title/Abstract] OR "Chi Kung"[Title/Abstract] OR "Pilates-Based Exercises"[Title/Abstract] OR "Exercises, Pilates-Based"[Title/Abstract] OR "Pilates Based Exercises"[Title/Abstract] OR "Pilates Training"[Title/Abstract] OR "Training, Pilates"[Title/Abstract] OR "baduanjin"[Title/Abstract] OR "Pilates"[Title/Abstract] OR "baduanjin"[Title/Abstract] OR "ba duan jin"[Title/Abstract] OR "eight section brocades"[Title/Abstract] OR "eight trigrams boxing"[Title/Abstract] OR "eight-treasured exercises"[Title/Abstract] OR "eight pieces of brocade" OR "eight brocade section"[Title/Abstract] OR "eight-section brocade"[Title/Abstract] OR "Five-animal exercises"[Title/Abstract] OR "Five Animal Exercise"[Title/Abstract] OR "Five animal Frolics"[Title/Abstract] OR "movements of five animals"[Title/Abstract] OR "Five mimic-animal boxing"[Title/Abstract] OR "Wuqinxi"[Title/Abstract] OR "wu qin xi"[Title/Abstract] OR "Liuzijue"[Title/Abstract] OR "liu zi jue"[Title/Abstract] OR "six-character formula"[Title/Abstract] OR "Six Healing Sounds"[Title/Abstract] OR Yijinjing[Title/Abstract] OR "yi jin jing"[Title/Abstract] OR "muscle-bone strengthening exercise"[Title/Abstract] OR "the classics of tendon changing"[Title/Abstract] OR "mind-body therap*"[Title/Abstract] OR "Pilates"[Title/Abstract] OR "dance"[Title/Abstract] OR "dancing"[Title/Abstract] OR "dances"[Title/Abstract] OR "Ballet"[Title/Abstract] OR "Jazz Dance"[Title/Abstract] OR "Dance,Jazz"[Title/Abstract] OR "Tap Dance"[Title/Abstract] OR "Dance, Tap"[Title/Abstract] OR "Modern Dance"[Title/Abstract] OR "Dance, Modern"[Title/Abstract] OR "Hip-Hop Dance"[Title/Abstract] OR "Dance, Hip-Hop"[Title/Abstract] OR "Hip Hop Dance"[Title/Abstract] OR "Line Dancing"[Title/Abstract] OR "Dancing, Line"[Title/Abstract] OR "Salsa Dancing"[Title/Abstract] OR "Dancing, Salsa"[Title/Abstract] OR "Square Dance"[Title/Abstract] OR "Dance, Square"[Title/Abstract] OR "Pilates-Based Exercises"[Title/Abstract] OR "Exercises, Pilates-Based"[Title/Abstract] OR "Pilates Based Exercises"[Title/Abstract] OR "Pilates Training"[Title/Abstract])
#5 "aerobic exercise"[Title/Abstract] OR "aerobic training"[Title/Abstract] OR "multidisciplinary exercise program"[Title/Abstract] OR "Nordic

Walking"[Title/Abstract] OR "running"[Title/Abstract] OR "treadmill training"[Title/Abstract] OR "walking"[Title/Abstract]
#6 ("High Intensity Interval Training"[Title/Abstract] OR "High-Intensity Interval Trainings"[Title/Abstract] OR "Interval Training,High-Intensity"[Title/Abstract] OR HIIT[Title/Abstract]) OR "Training,High-Intensity Interval"[Title/Abstract] OR "Trainings, High-Intensity Interval"[Title/Abstract] OR "High-Intensity Intermittent Exercise"[Title/Abstract] OR "Exercise, High-Intensity Intermittent"[Title/Abstract] OR "Exercises, High-Intensity Intermittent"[Title/Abstract] OR "High-Intensity Intermittent Exercises"[Title/Abstract] OR "Sprint Interval Training"[Title/Abstract] OR "Sprint Interval Trainings"[Title/Abstract]
#7 "Exercise*"[Title/Abstract] OR "Physical Activity"[Title/Abstract] OR "Activities,Physical"[Title/Abstract] OR "Activity, Physical"[Title/Abstract] OR "Physical Activities"[Title/Abstract] OR "Exercise,Physical"[Title/Abstract] OR "Exercises, Physical"[Title/Abstract] OR "Physical Exercise"[Title/Abstract] OR "Physical Exercises"[Title/Abstract] OR "Exercise, Isometric"[Title/Abstract] OR "Exercises, Isometric"[Title/Abstract] OR "Isometric Exercises"[Title/Abstract] OR "Isometric Exercise"[Title/Abstract] OR "Exercise Training"[Title/Abstract] OR "Exercise Trainings"[Title/Abstract] OR "Training, Exercise"[Title/Abstract] OR "Trainings, Exercise"[Title/Abstract]
#8 ("randomized controlled trial*" OR "controlled clinical trial" OR "random*" OR "clinical trial*"OR "randomly" OR "trial" OR "clinical trial" OR "randomized controlled trial*" OR "cross-over" OR "RCT")
#9 #1 OR #2
#10 #3 OR #4 OR #5 OR #6 OR #7
#11 #8 AND #9 AND #10

3. Demographic characteristics of included studies

This supplementary table presents the demographic characteristics of the included studies. The table reports the first author and year of publication. CAE, continuous aerobic exercise. CE, combined aerobic and resistance exercise. RE, resistance exercise. HIIT, high-intensity interval training. TCS, traditional Chinese sports. CG, control group. UK, the United Kingdom. USA, the United States of America. NR, not reported. ITT, intention-to-treat analysis; PP, per-protocol analysis; completer, analysis including only participants who completed the intervention or had available outcome data.

Author/ Year	Country	Sample size	Sex (male/female)	Age (m±sd)	BMI (m±sd)	Duration of diabetes (years)	Intervention	Duration (weeks)	Frequency (sessions/week)	Session duration (min)	Insulin treatment	Medication or diet changes	Outcomes	Analysis population	Adherence reporting
Marcotte-Chénard 2021	Canada	14	0/14	66.90±6.26	32.13±7.41	10.07±8.24	HIIT	12	3	25	Yes	No	FBG, HbA1c	PP	reported: attendance 91.3±11.3% (HIIT)
		15	0/15	67.53±5.15	33.50±10.06	8.50±6.95	CAE	12	3	50				PP	reported: attendance 96.3±4.7% (CAE)
Baasch-Skytte 2020	Denmark	23	23/0	61.0±6.2	30.6±5.4	8.0±5.9	HIIT	10	3	28	Yes	Yes	HbA1c, FBG	completer	reported: compliance 84% (HIIT)
		21	21/0	61.2±7.1	30.7±4.4	7.0±5.7	CAE	10	3	50				completer	reported: compliance 86% (CAE)
Honkola 1997	Finland	18	12/6	62.0±8.49	NR	8.0±8.49	RE	20	2	30	NR	NR	HbA1c	NR	NR
		20	5/15	67.0±8.94	NR	8.0±8.94	CG							NR	not applicable: no exercise intervention
Middlebrooke 2006	UK	22	NR	61.8±7.7	31.8±4.5	3.8±4.2	CAE	24	3	50	NR	NR	HbA1c, FBG	completer	partially reported: attendance threshold ≥80% (62/78 sessions); actual rate not reported
		30	NR	64.6±6.8	29.9±5.4	4.9±4.4	CG							completer	not applicable: no exercise intervention
Kadoglou 2012	Greece	23	7/16	61.5±5.4	32.74±4.05	6±2.8	RE	12	3	50	NR	NR	HbA1c, FBG	completer	reported: exercise-session attendance 91±4%
		24	5/19	64.6±4.3	31.58±5.71	5.6±1.9	CG							completer	reported: control-group target 45.8%

Pandey 2015	Canada	21	16/5	68±9	32.3±2.1	NR	HIIT	12	5	30	No	No	HbA1c	NR	partially reported: logbooks and self-reported exercise minutes; no adherence rate
		19	12/7	65±9	32.4±1.9	NR	CAE	12	5	30				NR	partially reported: logbooks and self-reported exercise minutes; no adherence rate
Mitranun 2014	Thailand	14	5/9	61.7±10.10	29.4±2.62	20.5±1.50	CAE	12	3	30	Yes	NR	HbA1c	completer	partially reported: adherence monitored; no rate reported
		15	5/10	60.9±9.30	29.7±1.55	21.1±2.32	CG							completer	not applicable: no exercise intervention
Sparks 2013	The Netherlands	18	9/9	60.4±7.3	33.9±5.2	9.4±6.8	RE	36	3	45	NR	NR	HbA1c	NR	partially reported: supervised/monitored RE sessions; no attendance rate
		10	2/8	60.8±8.0	34.6±4.0	5.4±3.3	CG							NR	not applicable: no exercise intervention
Maillard 2016	France	8	0/8	69.0±2.83	32.6±4.81	NR	HIIT	16	2	30	Yes	Yes	HbA1c, FBG	completer	partially reported: completion/dropout flow for HIIT; no attendance rate
		8	0/8	69.0±2.83	29.7±3.39	NR	CAE	16	2	50				completer	partially reported: completion/dropout flow for CAE; no attendance rate
Van Ryckeghem 2022	Belgium	10	9/1	61±5	30.1±5.1	7±4	HIIT	24	3	30	NR	NR	HbA1c, FBG	completer	partially reported: supervised HIIT sessions; no attendance rate
		9	8/1	66±11	29.8±7.1	12±9	CAE	24	3	45				completer	partially reported: supervised CAE sessions; no attendance rate

Chien 2022	Taiwan	20	5/15	67.6±7.7	24.3±3.4	17.5±16.3	RE	12	3	30	NR	NR	HbA1c	completer	reported: adherence/compliance rate 82% (exercise group)
		20	2/18	67.3±6.1	25.5±3.7	13.6±7.6	CG							completer	not applicable: no exercise intervention
Byrkjeland 2015	Norway	52	45/7	64.6±7.6	29.1±4.0	10.33±7.62	CE	12	3	60	NR	NR	HbA1c, FBG	PP; ITT also reported	reported: total exercise adherence 67% (55%, 83%); <40% excluded from PP
		62	50/12	63.2±7.2	29.0±5.6	9.00±6.07	CG							PP; ITT also reported	not applicable: no exercise intervention
Tan Sijie 2012	China	15	8/7	65.9±4.2	25.2±2.5	16.7±6.7	CE	24	3	50	NR	NR	FBG, 2hPG, HbA1c	completer	partially reported: completion/dropout reported; no attendance rate
		10	5/5	64.8 ±6.8	25.8±2.5	15.4±7.3	CG							completer	not applicable: no exercise intervention
Gholami 2021	Iran	15	15/0	63±3	NR	NR	RE	12	3	90	NR	NR	HbA1c	completer	reported: exercise-session attendance 91–100%
		14	14/0	64±3	NR	NR	CG							completer	not applicable: no exercise intervention
Cai Hong 2018	China	27	13/14	64.54	24.25±2.52	4.93	TCS	12	3	30	NR	NR	HbA1c, FBG	NR	NR
		28	11/17	64.51	24.81±1.76	5.54	CG							NR	not applicable: no exercise intervention
Meng En 2014	China	100	50/50	68.4±3.2	26.7±1.5	2-23	TCS	12	7	45	NR	NR	FBG, 2hPG, HbA1c	NR	NR
		100	50/50	68.4±3.2	26.7±1.5	2-23	CG							NR	not applicable: no exercise intervention
Zhao Tianqi 2022	China	12	NR	67.43±1.77	25.67±0.97	8.48±2.47	TCS	12	7	60	NR	NR	FBG, HbA1c	NR	NR
		12	NR	67.78±1.93	25.29±1.23	8.22±2.56	CG							NR	not applicable: no exercise intervention
Yang Han 2019	China	56	34/22	65.73±3.25	NR	5.42±1.31	TCS	12	5	40	NR	NR	FBG, 2hPG, HbA1c	NR	NR

		56	35/21	65.64±3.14	NR	5.36±1.23	CG							NR	not applicable: no exercise intervention
Wu Yunchuan 2015	China	20	NR	63.9±7.6	NR	NR	TCS	12	5	60	NR	NR	FBG, HbA1c	NR	partially reported: daily Baduanjin practice recorded/weekly guidance; no rate
		20	NR	64.8±5.8	NR	NR	CAE	12	5	60				NR	partially reported: walking practice recorded/weekly guidance; no rate
		20	NR	65.3±6.0	NR	NR	CG							NR	not applicable: no exercise intervention
Xu Yuxin 2019	China	40	21/19	65.41±5.01	24.86±3.89	10-22	CAE	24	5	30	NR	NR	FBG, 2hPG, HbA1c	NR	NR
		44	23/21	65.46±4.98	25.01±4.02	10-22	CE	24	5	45				NR	NR
		36	18/18	66.32±5.35	25.66±3.35	10-22	CG							NR	not applicable: no exercise intervention
Wang Wenqing 2019	China	29	20/9	68.67±8.46	NR	6.15±2.68	TCS	12	5	35	NR	NR	FBG, 2hPG, HbA1c	completer	partially reported: weekly follow-up/recording for Baduanjin; no adherence rate
		28	18/10	67.43±9.22	NR	5.87±2.44	CG							completer	not applicable: no exercise intervention
Kan Wei 2021	China	200	105/95	72.33±10.24	26.39±2.33	3-15	CAE	24	14	40	NR	NR	FBG, 2hPG, HbA1c	NR	NR
		200	105/95	76.22±12.42	26.49±2.35	3-15	CG							NR	not applicable: no exercise intervention
Han Yanmei 2024	China	41	17/24	65.2±3.52	NR	NR	TCS	24	10	30	NR	NR	FBG, HbA1c	NR	partially reported: follow-up on Baduanjin practice; no adherence rate
		42	18/24	65.05±2.99	NR	NR	CG							NR	not applicable: no exercise intervention
Meng Qing 2018	China	40	21/19	61.4±11.6	25.57±2.59	12.5±5.9	CE	12	3	60	NR	NR	FBG, HbA1c	NR	NR

		40	19/21	61.1±7.6	25.74±2.12	10.9±4.7	CAE	12	3	45				NR	NR
		40	18/22	63.5±9.2	25.67±2.7	9.8±4.6	CG							NR	not applicable: no exercise intervention
Parra-Sánchez 2015	Spain	47	NR	73.2±4.8	31.3±3.9	NR	CAE	12	2	60	NR	NR	HbA1c	ITT	partially reported: completion 47/50; no attendance rate
		41	NR	72.6±5.1	32.7±4.3	NR	CG							ITT	reported: completion 41/50; no exercise adherence
Su Chi 2024	China	77	37/40	61.4±5.2	30.8±4.3	NR	CAE	24	3	30	NR	NR	HbA1c	ITT; PP sensitivity	partially reported: PP defined by satisfactory adherence; no attendance rate
		75	39/36	61.2±6	30.1±4.2	NR	CG							ITT; PP sensitivity	not applicable: control group; no exercise adherence reported
de Oliveira 2013	Portugal	10	4/6	66.7±6.3	29.6±2.9	13.3±11.0	CE	20	3	60	NR	NR	FBG, HbA1c	PP	reported: exercise-group compliance rate >80%; dropouts described
		13	6/7	65.5±5.6	30.8±4.3	11.3±8.5	CG							PP	not applicable: no exercise intervention
Balducci 2010	Italy	20	12/8	64.3±8.1	29.4±1.1	9.4±6.09	CAE	48	2	60	Yes	Yes	HbA1c	ITT	reported: supervised-session attendance 80%
		22	14/8	60.6±9.3	30.5±0.9	8.5±5.78	CE	48	2	60				ITT	reported: supervised-session attendance 81%
		20	11/9	61.1±7.1	30.9±1.1	7.8±5.27	CG							ITT	not applicable: no exercise intervention
Liu Xiaoli 2024	China	16	8/8	60.6±5.7	26.5±3.5	7.5±2.0	TCS	24	6	60	NR	NR	FBG, HbA1c	completer	partially reported: completion/non-completion flow; no attendance rate
		16	8/8	61.3±4.9	26.3±2.2	7.4±2.3	CG							completer	not applicable: no exercise intervention

Wang Yu 2023	China	30	10/20	61.83±6.59	29.5±4.68	NR	TCS	4	5	30	NR	NR	HbA1c, FBG, 2hPG	completer	reported: completed intervention 30/30; no dropouts
		30	12/18	63.33±6.56	29.83±4.35	NR	CAE							completer	reported: completed control intervention 30/30; no dropouts
Su Xiaoyun 2022	China	14	0/14	64.01±1.98	24.9±0.67	8.14±1.84	CE	12	3	60	Yes	NR	FBG, 2hPG	completer	partially reported: final sample after withdrawals; no attendance rate
		13	0/13	63.61±2.56	25.67±0.96	7.89±2.46	CG							completer	not applicable: no exercise intervention
Castaneda 2002	USA	31	10/21	66.0±11.14	30.9±6.12	8.0±5.57	RE	16	3	45	Yes	Yes	HbA1c, FBG	completer	reported: PRT compliance 90±10%
		31	12/19	66.0±5.57	31.2±5.57	11.0±5.57	CG							completer	not applicable: no exercise intervention
Gordon 2008	Jamaica	77	62/15	63.9	NR	NR	CAE	24	2	90	NR	NR	FBG	completer	reported: home exercise compliance 80–85%; class attendance 90–95%
		77	62/15	63.6	NR	NR	CG							completer	not applicable: no exercise intervention
Mahmoud reza 2017	Iran	10	0/10	62.25±3.81	30.9±3.4	NR	CAE	8	3	40	NR	NR	HbA1c, FBG	NR	NR
		10	0/10	62.25±3.81	30.3±3.6	NR	CG							NR	not applicable: no exercise intervention

1. Marcotte-Chénard A, Tremblay D, Mony M-M, Boulay P, Brochu M, Morais JA, Dionne IJ, Langlois M-F, Mampuya WM, Tessier DM. Acute and chronic effects of low-volume high-intensity interval training compared to moderate-intensity continuous training on glycemic control and body composition in older women with type 2 diabetes. *Obesities* 2021, 1(2):72–87. doi: 10.3390/Obesities1020007.

2. Balducci S, Zanuso S, Nicolucci A, Fernando F, Cavallo S, Cardelli P, Fallucca S, Alessi E, Letizia C, Jimenez A *et al*. Anti-inflammatory effect of exercise training in subjects with type 2 diabetes and the metabolic syndrome is dependent on exercise modalities and independent of weight loss. *Nutr Metab Cardiovasc Dis* 2010, 20(8):608–617. doi: 10.1016/j.numecd.2009.04.015.

3. Yunchuan W, Qingbo W. Clinical efficacy of Baduanjin in the adjunctive treatment of type 2 diabetes mellitus. *Chinese Journal of Gerontology* 2015, 35(18):5218–5219.

4. de Oliveira CFC. Combined exercise in individuals with type 2 diabetes: mainly aerobic or resistance training? The DICE intervention. Universidade do Porto (Portugal); 2013.

5. Mitranun W, Deerochanawong C, Tanaka H, Suksom D. Continuous vs interval training on glycemic control and macro- and microvascular reactivity in type 2 diabetic patients. *Scand J Med Sci Sports* 2014, 24(2):e69–76. doi: 10.1111/sms.12112.

6. Su C, Huang L, Tu S, Lu S. Different intensities of aerobic training for patients with type 2 diabetes mellitus and knee osteoarthritis: a randomized controlled trial. *Front Endocrinol (Lausanne)* 2024, 15.1463587. doi: 10.3389/fendo.2024.1463587.

7. Gordon LA, Morrison EY, McGrowder DA, Young R, Fraser YT, Zamora EM, Alexander-Lindo RL, Irving RR. Effect of exercise therapy on lipid profile and oxidative stress indicators in patients with type 2 diabetes. *BMC Complement Altern Med* 2008, 8:21. doi: 10.1186/1472-6882-8-21.

8. En M. Effect of tai chi exercise on lipid composition and insulin resistance in type 2 diabetic patients. *Chinese Journal of Gerontology* 2014, 34(19):5358–5360.

9. Chien YH, Tsai CJ, Wang DC, Chuang PH, Lin HT. Effects of 12-Week Progressive Sandbag Exercise Training on Glycemic Control and Muscle Strength in Patients with Type 2 Diabetes Mellitus Combined with Possible Sarcopenia. *Int J Environ Res Public Health* 2022, 19(22). doi: 10.3390/ijerph192215009.

10. Yu W. Effects of a Wuqinxi Exercise Prescription in Patients with Type 2 Diabetes. *D.* Guangxi University of Chinese Medicine; 2023.

11. Qing M, Wei C, Ming Z, Min G. Effects of aerobic combined with resistance exercise on patients with type 2 diabetes. *Chinese Journal of Rehabilitation Theory and Practice* 2018, 24(12):1465–1470.

12. Su X, He J, Cui J, Li H, Men J. The effects of aerobic exercise combined with resistance training on inflammatory factors and heart rate variability in middle-aged and elderly women with type 2 diabetes mellitus. *Ann Noninvasive Electrocardiol* 2022, 27(6):e12996. doi: 10.1111/anec.12996.

13. Tianqi Z. Effects of Baduanjin Alone and Baduanjin Combined with Resistance Exercise on Hemorheology in Older Adults with Type 2 Diabetes. *D.* Liaoning Normal University; 2022.

14. Wenqing W. Effects of baduanjin on angina symptoms, metabolic equivalents, and quality of life in patients with stable coronary angina and type 2 diabetes. *D.* Anhui University of Chinese Medicine; 2019.

15. Yanmei H, Lanlan D. Effects of Baduanjin on Patients with Type 2 Diabetes and Mild to Moderate Anxiety and Depression. *China's Naturopathy* 2024, 32(11):52–55. doi: 10.19621/j.cnki.11-3555/r.2024.1117.

16. Yuxin X, Qingping J, Cuihong Z. Effects of combined aerobic and resistance exercise on oxidative stress and glucose and lipid metabolism in older adults with type 2 diabetes. *Chinese Journal of Gerontology* 2019, 39(03):591–593.

17. Han Y, Peng L, Zhaohui F, Yunchuan W. Effects of community-managed gongfu badanjin on clinical outcomes, psychological status and glycemic indexes in elderly patients with type 2 diabetes mellitus. *Chinese Journal of Gerontology* 2019, 39(14):3433–3435.

18. Taghizadeh M, Ahmadizad S, Naderi M. Effects of endurance training on hsa-miR-223, P2RY12 receptor expression and platelet function in type 2 diabetic patients. *Clin Hemorheol Microcirc* 2018, 68(4):391–399. doi: 10.3233/ch-170300.

19. Byrkjeland R, Njerve IU, Anderssen S, Arnesen H, Seljeflot I, Solheim S. Effects of exercise training on HbA1c and VO2peak in patients with type 2 diabetes and coronary artery disease: A randomised clinical trial. *Diab Vasc Dis Res* 2015, 12(5):325–333. doi: 10.1177/1479164115590552.

20. Kadoglou NP, Fotiadis G, Athanasiadou Z, Vitta I, Lampropoulos S, Vrabas IS. The effects of resistance training on ApoB/ApoA-I ratio, Lp(a) and inflammatory markers in patients with type 2 diabetes. *Endocrine* 2012, 42(3):561–569. doi: 10.1007/s12020-012-9650-y.

21. Tan S, Li W, Wang J. Effects of six months of combined aerobic and resistance training for elderly patients with a long history of type 2 diabetes. *J Sports Sci Med* 2012, 11(3):495–501.

22. Wei K, Meifang T, Yan Z. Effects of square fitness dance on sustainable health development in middle-aged and older adults with type 2 diabetes. *International Journal of Geriatrics* 2021, 42(02):111–113.

23. Baasch-Skytte T, Lemgart CT, Oehlenschläger MH, Petersen PE, Hostrup M, Bangsbo J, Gunnarsson TP. Efficacy of 10-20-30 training versus moderate-intensity continuous training on HbA1c, body composition and maximum oxygen uptake in male patients with type 2 diabetes: A randomized controlled trial. *Diabetes Obes Metab* 2020, 22(5):767–778. doi: 10.1111/dom.13953.

24. Parra-Sánchez J, Moreno-Jiménez M, Nicola CM, Nocua R, II, Amegló-Parejo MR, Del Carmen-Peña M, Cordero-Prieto C, Gajardo-Barrena MJ. [Evaluation of a supervised physical exercise program in sedentary patients over 65 years with type 2 diabetes mellitus]. *Aten Primaria* 2015, 47(9):555–562. doi: 10.1016/j.aprim.2015.01.006.

25. Maillard F, Rousset S, Pereira B, Traore A, de Pradel Del Amaze P, Boirie Y, Duclos M, Boisseau N. High-intensity interval training reduces abdominal fat mass in postmenopausal women with type 2 diabetes. *Diabetes Metab* 2016, 42(6):433–441. doi: 10.1016/j.diabet.2016.07.031.

26. Van Ryckeghem L, Keytsman C, De Brandt J, Verboven K, Verbaanderd E, Marinus N, Franssen WMA, Frederix I, Bakelants E, Petit T *et al*. Impact of continuous vs. interval training on oxygen extraction and cardiac function during exercise in type 2 diabetes mellitus. *Eur J Appl Physiol* 2022, 122(4):875–887. doi: 10.1007/s00421-022-04884-9.

27. Pandey A, Suskin N, Poirier P. The Impact of Burst Exercise on Cardiometabolic Status of Patients Newly Diagnosed With Type 2 Diabetes. Can J Cardiol. 2017;33(12):1645–51. doi:10.1016/j.cjca.2017.09.019.

28. Sparks LM, Johannsen NM, Church TS, Earnest CP, Moonen-Kornips E, Moro C, Hesselink MK, Smith SR, Schrauwen P. Nine months of combined training improves ex vivo skeletal muscle metabolism in individuals with type 2 diabetes. *J Clin Endocrinol Metab* 2013, 98(4):1694–1702. doi: 10.1210/jc.2012-3874.

29. Castaneda C, Layne JE, Munoz-Orians L, Gordon PL, Walsmith J, Foldvari M, Roubenoff R, Tucker KL, Nelson ME. A randomized controlled trial of resistance exercise training to improve glycemic control in older adults with type 2 diabetes. *Diabetes Care* 2002, 25(12):2335–2341. doi: 10.2337/diacare.25.12.2335.

30. Gholami F, Khaki R, Mirzaei B, Howatson G. Resistance training improves nerve conduction and arterial stiffness in older adults with diabetic distal symmetrical polyneuropathy: A randomized controlled trial. *Exp Gerontol* 2021, 153:111481. doi: 10.1016/j.exger.2021.111481.

31. Honkola A, Forsén T, Eriksson J. Resistance training improves the metabolic profile in individuals with type 2 diabetes. *Acta Diabetol* 1997, 34(4):245–248. doi: 10.1007/s005920050082.

32. Middlebrooke AR, Elston LM, Macleod KM, Mawson DM, Ball CI, Shore AC, Tooke JE. Six months of aerobic exercise does not improve microvascular function in type 2 diabetes mellitus. *Diabetologia* 2006, 49(10):2263–2271. doi: 10.1007/s00125-006-0361-x.

33. Hong C. A Study of the Intervention Effects of Kinect-Based Open-Meridian Tai Chi in Older Adults with Type 2 Diabetes. *D.* Jilin University; 2018.

34. Liu X, Zhu H, Peng Y, Liu Y, Shi X. Twenty-Four week Taichi training improves pulmonary diffusion capacity and glycemic control in patients with Type 2 diabetes mellitus. *PLoS One* 2024, 19(4):e0299495. doi: 10.1371/journal.pone.0299495.

4. Risk of bias

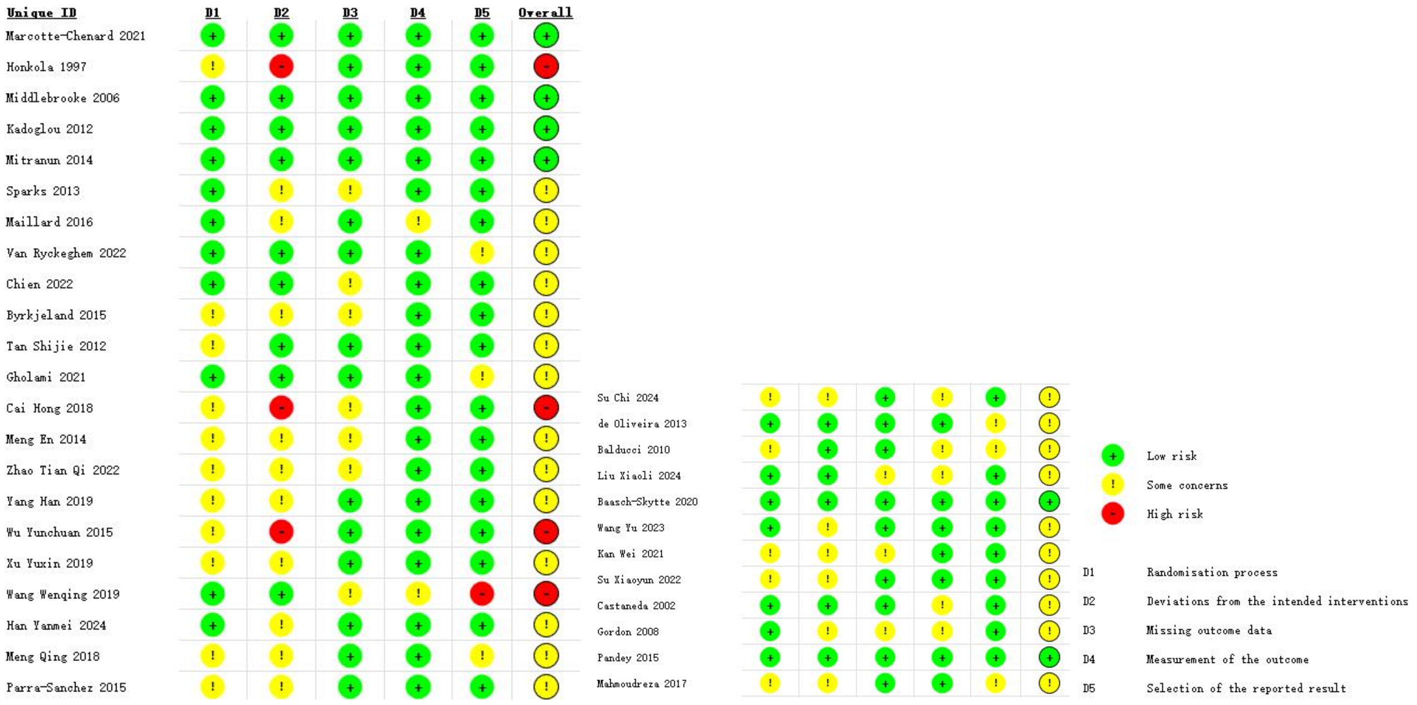

Figure S1. Detailed risk-of-bias assessment

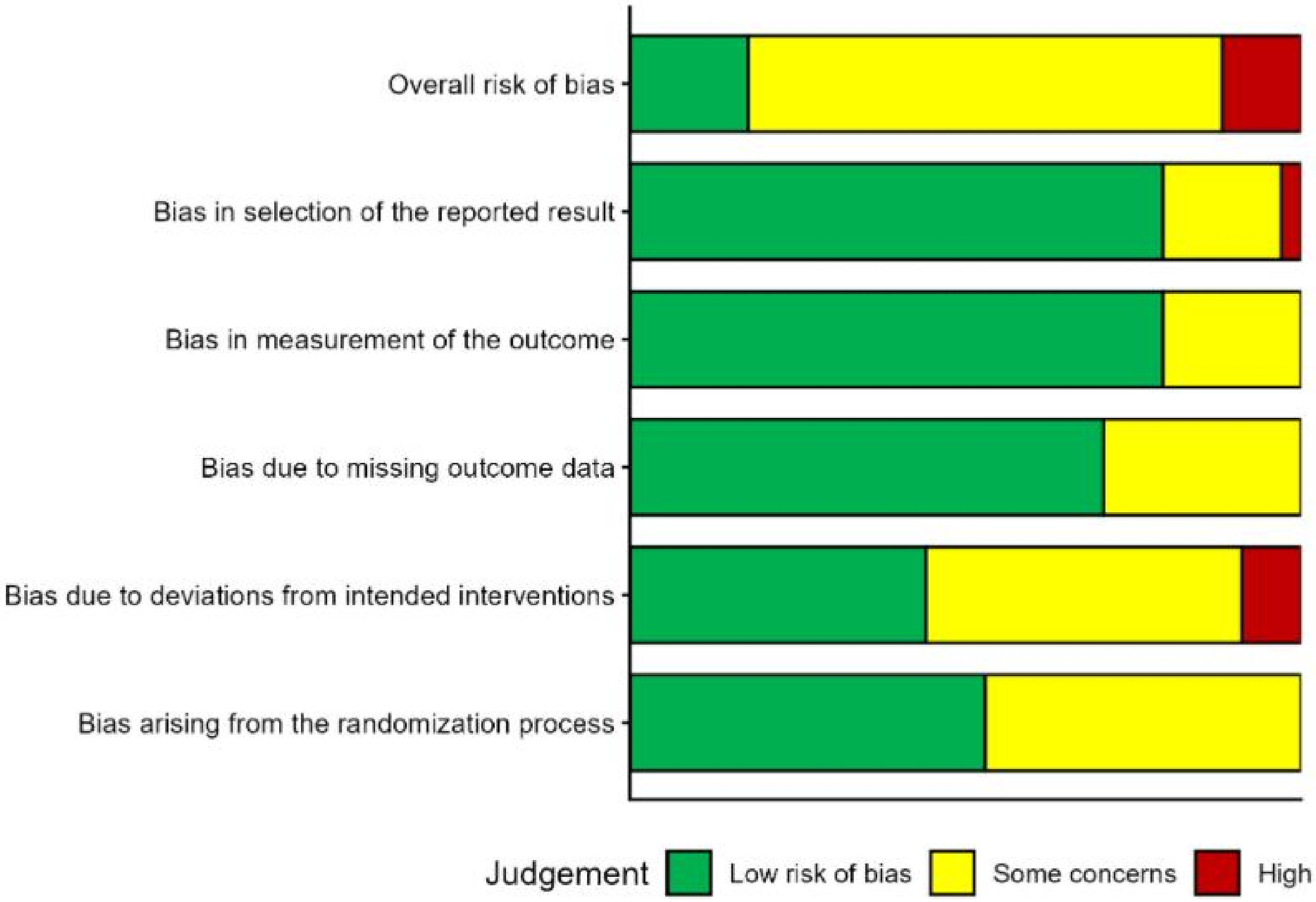

Figure S2. Summary of risk of bias

5. Pairwise meta-analyses and publication bias

5.1 Details of pairwise meta-analyses

Table S1. Details of pairwise meta-analyses

HbA1c

Comparison	Number of studies	MD	95% CrI	I^2
CAE vs CG	10	**-0.49**	**(-0.89, -0.12)**	86.3
CE vs CG	6	**-1.09**	**(-1.64, -0.52)**	49.3
RE vs CG	6	-0.46	(-0.99, 0.06)	89.2
TCS vs CG	8	**-0.71**	**(-0.99, -0.47)**	47.7

FBG

Comparison	Number of studies	MD	95% CrI	I^2
CAE vs CG	7	**-1.28**	**(-1.89, -0.66)**	90.6
CE vs CG	6	**-1.31**	**(-2.05, -0.48)**	49.9
RE vs CG	2	-0.6	(-1.82, 0.38)	73.1
TCS vs CG	8	**-0.55**	**(-0.89, -0.27)**	28.5

2hPG

Comparison	Number of studies	MD	95% CrI	I^2
CAE vs CG	2	**-1.51**	**(-2.71, -0.61)**	60
CE vs CG	3	**-3.31**	**(-4.86, -1.73)**	52.3
TCS vs CG	3	**-1.15**	**(-2.05, -0.29)**	77.7

Note: Bolded numbers indicate the presence of significance. MD: mean difference. CrI: credible interval.

5.2 Publication bias

HbA1c

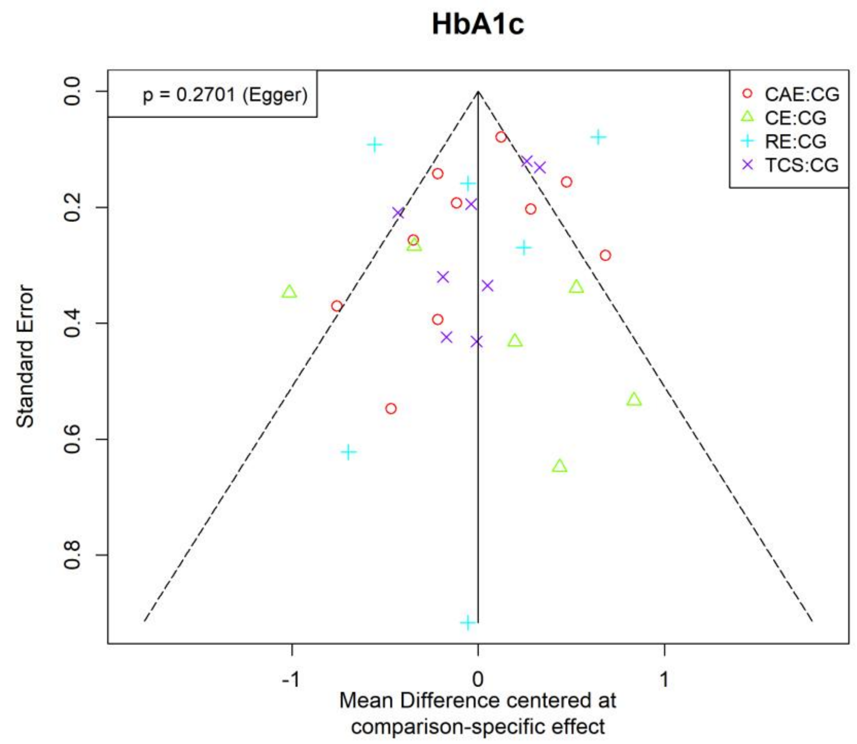

FBG

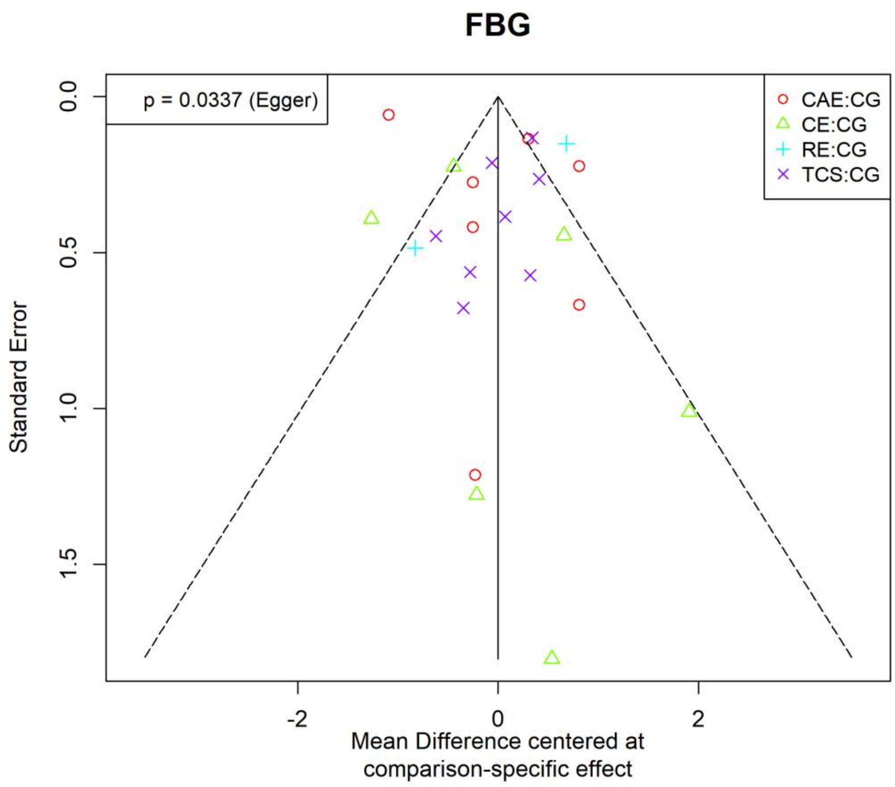

Figure S3. Funnel plot. CAE, continuous aerobic exercise. CE, combined aerobic and resistance exercise. RE, resistance exercise. TCS, traditional Chinese sports

6. Bayesian network meta-analysis

6.1 Model fit summaries for included studies

Table S2. Model fit summaries for included studies.

HbA1c

Model	HbA1c (68 data points)			
	DIC	pD	Residual Deviance	SD (95%CrI)
FE Model	241.7	37.0	204.7	-
RE Model	121.2	55.9	65.3	0.34 (0.24, 0.50)
RE UME	120.0	56.0	64.0	-

FBG

Model	FBG (53 data points)			
	DIC	pD	Residual Deviance	SD (95%CrI)
FE Model	413.5	30	383.5	-
RE Model	96.1	45.1	51	0.67 (0.46, 1.02)
RE UME	93.8	44.1	49.7	-

2hPG

Model	2hPG (17 data points)			
	DIC	pD	Residual Deviance	SD (95%CrI)
FE Model	76	11.1	65	-
RE Model	32.6	16	16.6	1.41 (0.69, 3.37)
RE UME	32.5	16.1	16.5	-

Note: CrI, credible interval. Abbreviations: DIC, deviance information criterion; FE, fixed effects; pD, number of effective parameters; RE, random effects; UME, unrelated mean effects.

6.2 Node-split for all studies

Table S3. Node-split for all studies

HbA1c

Comparison	p.value	95%CrI
d.CAE.CE	0.40575	
-> direct		-0.53 (-1.0, -0.0012)
-> indirect		-0.16 (-0.84, 0.55)
-> network		-0.56 (-0.98, -0.12)
d.CAE.CG	0.296	
-> direct		0.57 (0.31, 0.84)
-> indirect		0.16 (-0.58, 0.94)
-> network		0.48 (0.24, 0.74)
d.CAE.TCS	0.32375	
-> direct		-0.52 (-1.0, 0.040)
-> indirect		-0.18 (-0.61, 0.24)
-> network		-0.32 (-0.65, 0.016)
d.CG.TCS	0.5065	

-> direct		-0.76 (-1.1, -0.46)
-> indirect		-1.0 (-1.9, -0.23)
-> network		-0.80 (-1.1, -0.52)

FBG

Comparison	p.value	95%CrI
d.CAE.CE	0.0335	
-> direct		-1.1 (-2.1, -0.11)
-> indirect		0.74 (-0.55, 2.1)
-> network		-0.46 (-1.3, 0.39)
d.CAE.CG	0.07475	
-> direct		1.3 (0.74, 1.8)
-> indirect		-0.019 (-1.3, 1.3)
-> network		0.97 (0.42, 1.5)
d.CAE.TCS	0.26775	
-> direct		-0.26 (-1.3, 0.83)
-> indirect		0.51 (-0.42, 1.3)
-> network		0.21 (-0.47, 0.90)
d.CG.TCS	0.23425	
-> direct		-0.66 (-1.2, -0.096)
-> indirect		-1.7 (-3.2, -0.058)
-> network		-0.76 (-1.3, -0.24)

2hPG

Comparison	p.value	95%CrI
d.CAE.CE	0.4209	
-> direct		-2.4 (-5.5, 0.59)
-> indirect		-0.95 (-4.9, 2.5)
-> network		-2.1 (-4.8, 0.70)
d.CAE.CG	0.3016	
-> direct		1.8 (-0.58, 4.2)
-> indirect		0.043 (-3.8, 3.9)
-> network		1.1 (-1.0, 3.2)
d.CAE.TCS	0.508	
-> direct		-1.1 (-5.0, 2.8)
-> indirect		0.24 (-3.4, 3.8)
-> network		-0.37 (-2.7, 2.)
d.CG.TCS	0.5018	
-> direct		-1.2 (-3.5, 1.1)
-> indirect		-2.6 (-7.3, 2.1)
-> network		-1.4 (-3.3, 0.38)

Note: CrI, credible interval. CG, control group. CAE, continuous aerobic exercise. CE, combined aerobic and resistance exercise. RE, resistance exercise. HIIT, high-intensity interval training. TCS, traditional Chinese sports.

6.3 Forest plot of all studies

HbA1c

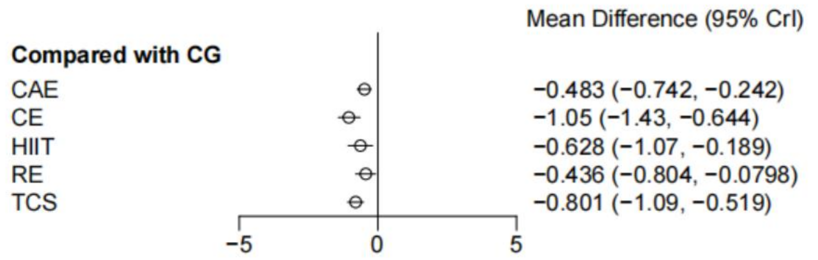

FBG

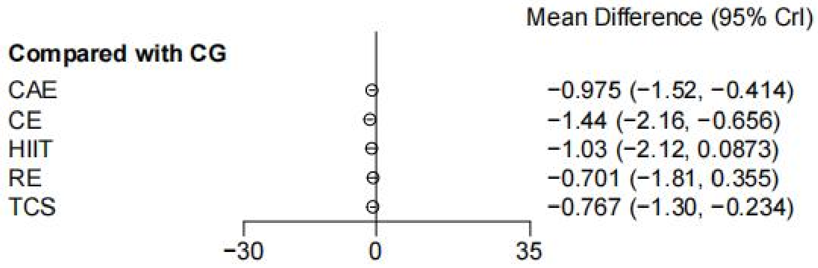

2hPG

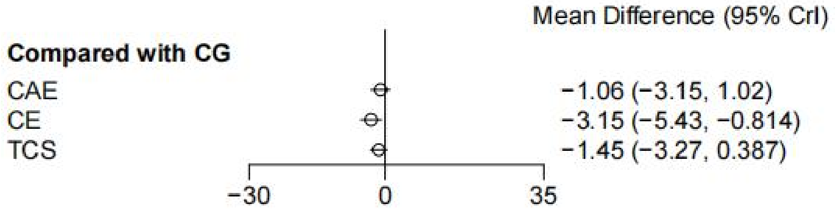

Figure S4. Forest plot for all studies. CrI, credible interval. CG, control group. CAE, continuous aerobic exercise. CE, combined aerobic and resistance exercise. RE, resistance exercise. HIIT, high-intensity interval training. TCS, traditional Chinese sports.

6.4 SUCRA table for all studies

Table S4. SUCRA table for all studies

HbA1c

Treatment	Rank 1	Rank 2	Rank 3	Rank 4	Rank 5	Rank 6	SUCRA
CAE	0	0.008	0.147	0.524	0.322	0	36.82
CE	0.803	0.148	0.04	0.008	0.002	0	94.86
CG	0	0	0	0	0.013	0.987	0.26
HIIT	0.05	0.201	0.421	0.201	0.123	0.004	56.87
RE	0.006	0.042	0.182	0.228	0.533	0.009	34.64
TCS	0.141	0.602	0.211	0.039	0.008	0	76.56

FBG

Treatment	Rank 1	Rank 2	Rank 3	Rank 4	Rank 5	Rank 6	SUCRA
CAE	0.039	0.263	0.409	0.224	0.064	0	59.76
CE	0.625	0.227	0.088	0.043	0.016	0	88.03
CG	0	0	0	0.005	0.123	0.872	2.65
HIIT	0.215	0.256	0.177	0.166	0.154	0.032	62.30
RE	0.096	0.143	0.125	0.185	0.36	0.091	43.12
TCS	0.024	0.112	0.2	0.378	0.283	0.003	44.14

2hPG

Treatment	Rank 1	Rank 2	Rank 3	Rank 4	SUCRA
CAE	0.036	0.318	0.537	0.109	42.59
CE	0.882	0.085	0.028	0.005	94.71
CG	0.001	0.019	0.132	0.848	5.81
TCS	0.081	0.578	0.303	0.038	56.88

Note: CG, control group. CAE, continuous aerobic exercise. CE, combined aerobic and resistance exercise. RE, resistance exercise. HIIT, high-intensity interval training. TCS, traditional Chinese sports.

6.5 Assessment of transitivity assumption

Effect modifier	HIIT	CAE	CE	RE	TCS	CG	*p* value
Number of arms, n	5	16	6	6	9	26	—
Sample size	15.20 ± 6.61, n=5	37 ± 47.0, n=16	30.70 ± 17.40, n=6	20.80 ± 5.64, n=6	36.80 ± 27.20, n=9	37.40 ± 39.70, n=26	0.399
Baseline HbA1c, %	7.23±0.68, n=5	7.62±0.72, n=16	7.64±0.73, n=6	8.00±0.80, n=6	7.48±0.70, n=9	7.60±0.91, n=26	0.730
Diabetes duration, years, mean ± SD	8.36±1.57, n=3	10.3±5.24, n=7	12.3±3.11, n=5	9.78±4.48, n=5	6.50±1.47, n=5	9.13±4.33, n=16	0.142
Age, years, mean ± SD	65.2±3.91, n=5	65.1±3.85, n=16	64.1±2.52, n=6	63.4±2.80, n=6	65.1±2.79, n=9	65.3±3.53, n=26	0.907
Publication date, years, mean ± SD	2019±3.11, n=5	2017±4.81, n=16	2014±3.51, n=6	2011±10.0, n=6	2020±3.73, n=9	2016±6.63, n=26	0.130
**Effect modifier**	**HIIT**	**CAE**	**CE**	**RE**	**TCS**	**CG**	***p* value**
Number of arms, n	4	12	6	2	9	20	
Sample size	13.8± 6.65, n=4	41± 53.6, n=12	29.3± 18.5, n=6	27± 5.66, n=2	36.8± 27.2, n=9	42.4± 44.1, n=20	0.377
Baseline FBG, mmol/L	8.60±1.25, n=4	8.68±1.39, n=12	8.75±1.33, n=6	9.10±0.42, n=2	8.04±1.01, n=9	8.51±1.43, n=20	0.780
Diabetes duration, years, mean ± SD	8.36±1.57, n=3	8.44±3.25, n=5	12.2±3.22, n=5	7±1.41, n=2	6.50±1.47, n=5	8.25±3.05, n=13	0.063
Age, years, mean ± SD	64.5± 4.14, n=4	64.9± 3.46, n=12	64.7± 1.86, n=6	63.8± 3.18, n=2	65.1± 2.79, n=9	65.5± 3.09, n=20	0.989
Publication date, years, mean ± SD	2020± 2.63, n=4	2017± 5.34, n=12	2016± 3.83, n=6	2007± 7.07, n=2	2020± 3.73, n=9	2016± 5.93, n=20	0.120
**Effect modifier**	**CAE**	**CE**	**TCS**	**CG**			***p* value**
Number of arms, n	3	3	4	7			
Sample size	90±95.4, n=3	24.3±17.0, n=3	53.8±33.3, n=4	63.3±67.6, n=7			0.584
Baseline 2hPG, mmol/L	11.7±3.6, n=3	14.5±0.57, n=3	10.1±2.01, n=4	12.1±1.93, n=7			0.134
Diabetes duration, years, mean ± SD		12.4± 6.05, n=2	5.78± 0.52, n=2	8.63±4.64, n=4			0.223
Age, years, mean ± SD	64.2±1.06, n=3	67.9±3.85, n=3	66.2±3.18, n=4	67.5±4.17, n=7			0.294
Publication date, years, mean ± SD	2021±2, n=3	2018±5.13, n=3	2019±3.69, n=4	2018±3.65, n=7			0.607

Table S5. Distribution of potential effect modifiers across intervention groups for HbA1c, FBG, and 2hPG networks

HbA1c

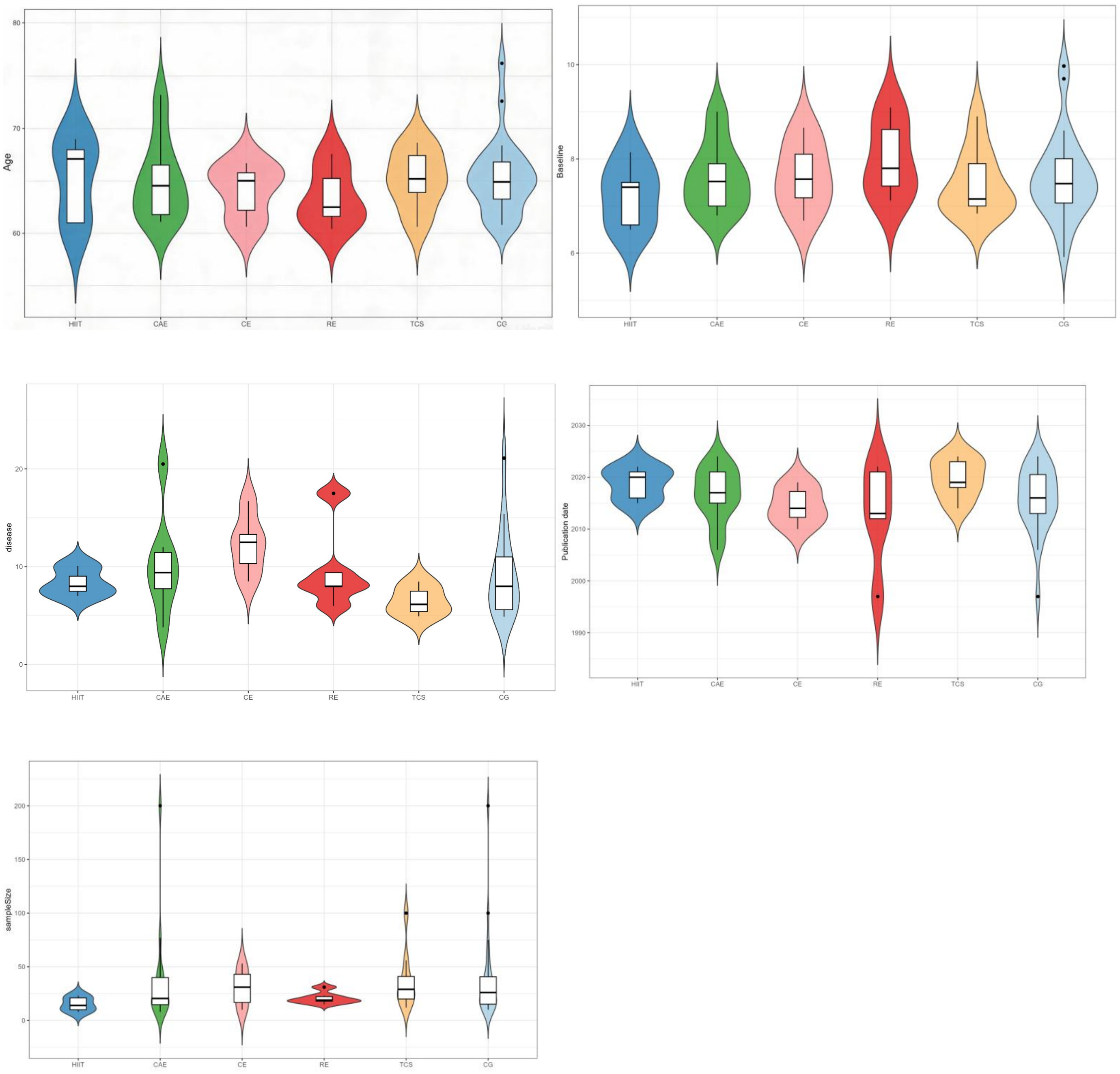

FBG

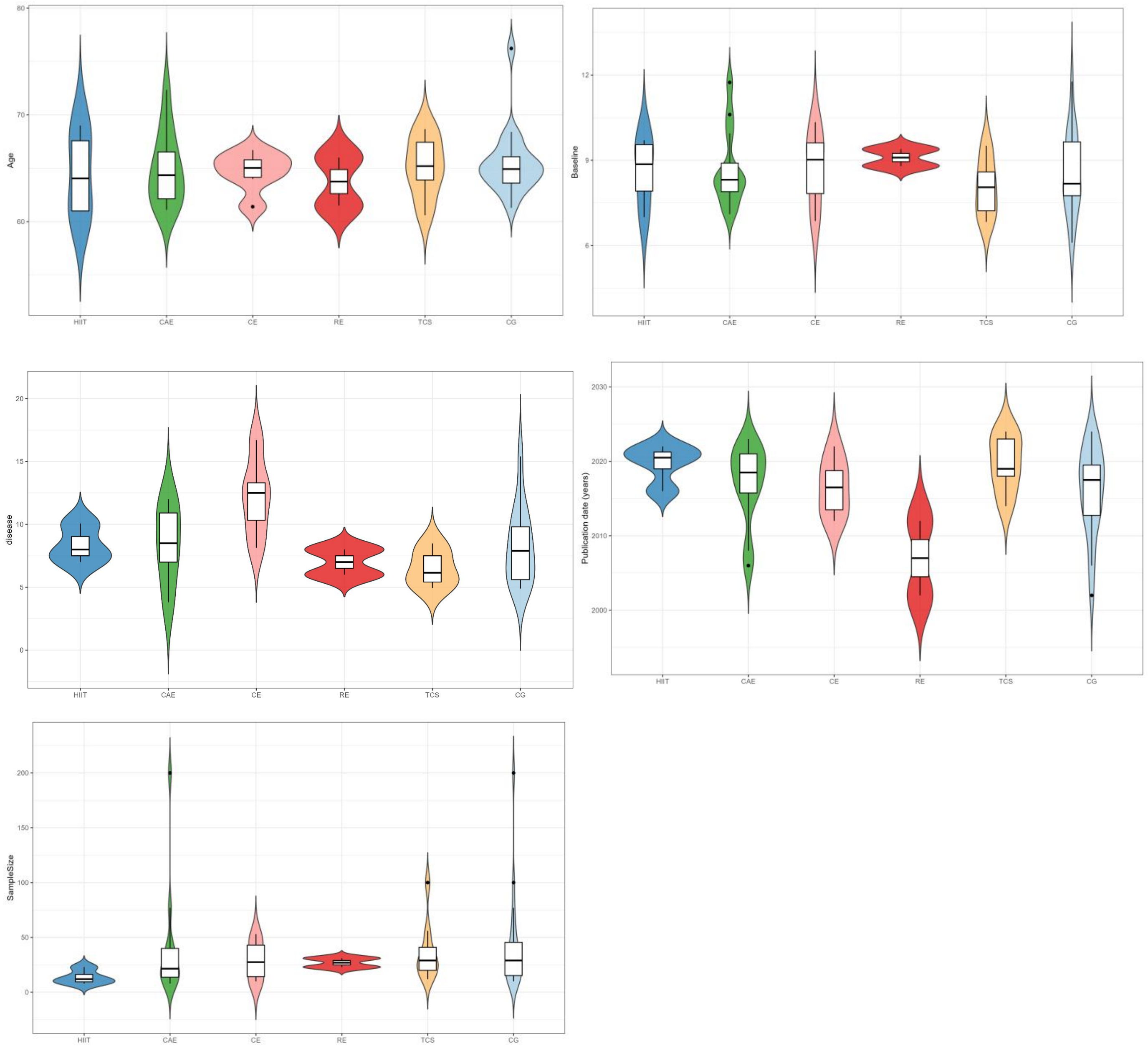
Age
Baseline
disease
Publication date (years)
SampleSize
HIIT
CAE
CE
RE
TCS
CG

2hPG

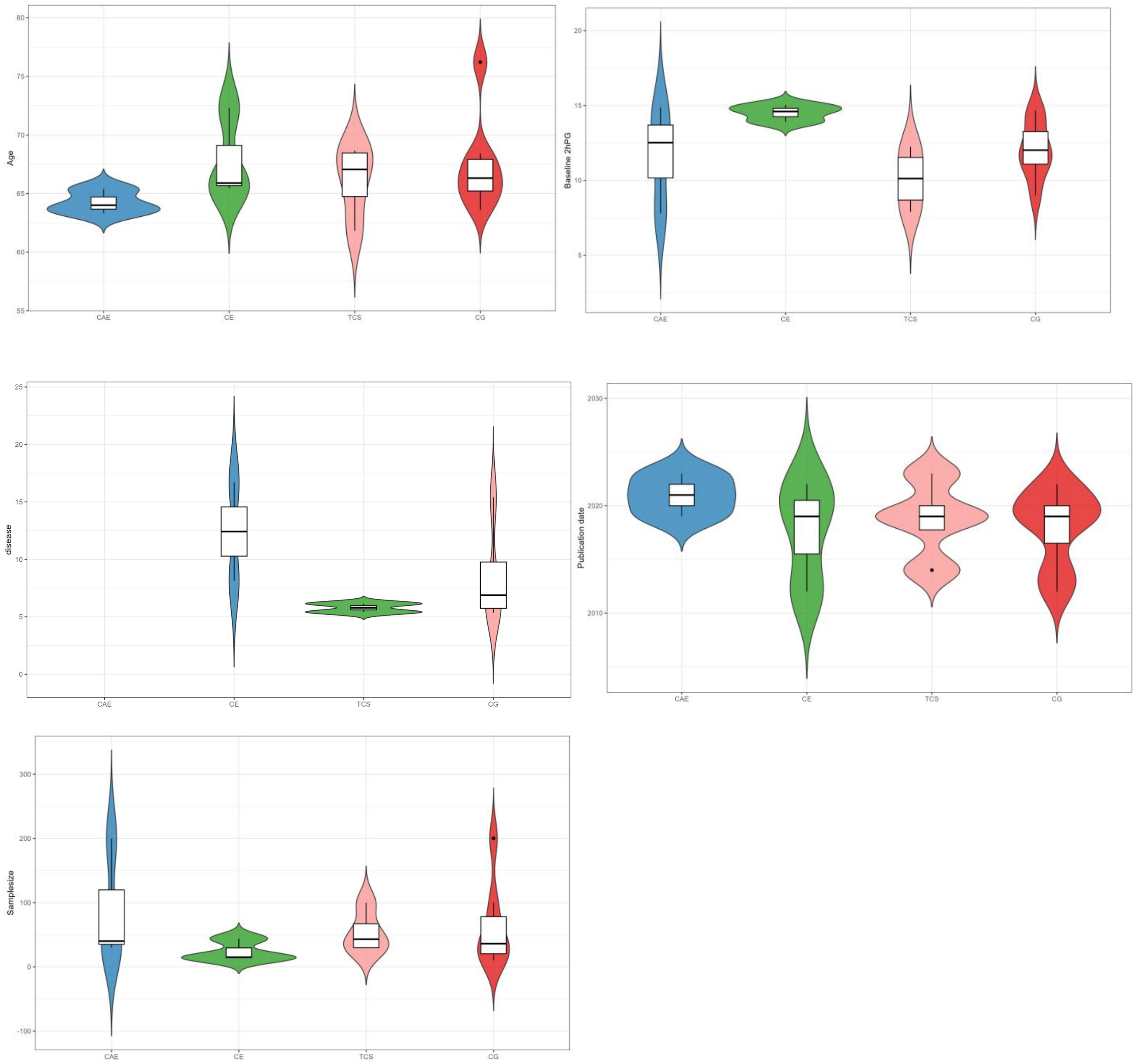

Figure S5. Distribution of potential effect modifiers across intervention nodes. The five panels show the distributions of age, baseline level, diabetes duration (years), publication year, and sample size across the intervention nodes.

6.6 Sensitivity analysis

6.6.1 Model fit summaries for all studies and sensitivity analyses

Table S6. Model fit summaries for all studies and sensitivity analyses
HbA1c

	HbA1c			
	DIC	pD	Residual Deviance	SD(95%CrI)
Model	All studies (68 data points)			
FE Model	241.7	37.0	204.7	-
RE Model	121.2	55.9	65.3	0.34 (0.24, 0.50)
RE UME	120.0	56.0	64.0	-
	Exclude high risk of bias (59 data points)			

FE Model	222.4	33	189.3	-
RE Model	107.3	49.6	57.6	0.36 (0.24, 0.54)
RE UME	106.4	50	56.4	-
	Exclude interventions less than 12 weeks (62 data points)			
FE Model	224.3	34	190.3	-
RE Model	110.8	51.3	59.5	0.35 (0.24, 0.52)
RE UME	110	51.7	58.3	-

FBG

Model	FBG			
	DIC	pD	Residual Deviance	SD(95%CrI)
	All studies (53 data points)			
FE Model	413.5	30	383.5	-
RE Model	96.1	45.1	51	0.67 (0.46, 1.02)
RE UME	93.8	44.1	49.7	-
	Exclude high risk of bias (45 data points)			
FE Model	343.4	27	316.4	-
RE Model	82.7	39.2	43.5	0.77 (0.50, 1.26)
RE UME	82.8	39.4	43.4	-
	Exclude interventions less than 12 weeks (47 data points)			
FE Model	255	27	228	-
RE Model	86.6	40.3	46.3	0.66 (0.43, 1.08)
RE UME	85	40.1	44.9	-

2hPG

Model	2hPG			
	DIC	pD	Residual Deviance	SD(95%CrI)
	All studies (17 data points)			
FE Model	76	11.1	65	-
RE Model	32.6	16	16.6	1.41 (0.69, 3.37)
RE UME	32.5	16.1	16.5	-
	Exclude high risk of bias (15data points)			
FE Model	47.4	10	37.4	-
RE Model	28.9	14.2	14.7	1.46 (0.61, 3.84)
RE UME	28.9	14.2	14.7	-
	Exclude interventions less than 12 weeks (15 data points)			
FE Model	58.6	10.1	48.5	-

RE Model	28.8	14.2	14.6	1.51 (0.68, 3.83)
RE UME	28.5	14	14.5	-

Note: CrI, credible interval. DIC, deviance information criterion. FE, fixed effects. pD, number of effective parameters. SD, standard deviation. RE, random effects. UME, unrelated mean effects.

6.6.2 Node-split for sensitivity analyses

Table S7. Node-split for sensitivity analyses

HbA1c

a.Exclude high risk of bias

Comparison	p.value	95%CrI
d.CAE.CE	0.42575	
-> direct		-0.53 (-1.1, 0.0028)
-> indirect		-0.18 (-0.91, 0.55)
-> network		-0.55 (-0.99, -0.095)
d.CAE.CG	0.41475	
-> direct		0.56 (0.27, 0.86)
-> indirect		0.20 (-0.66, 1.1)
-> network		0.48 (0.21, 0.77)
d.CAE.TCS	0.48525	
-> direct		-0.53 (-1.3, 0.25)
-> indirect		-0.22 (-0.74, 0.31)
-> network		-0.32 (-0.72, 0.11)
d.CG.TCS	0.484	
-> direct		-0.73 (-1.1, -0.33)
-> indirect		-1.0 (-1.9, -0.18)
-> network		-0.80 (-1.2, -0.43)

b.Exclude interventions less than 12 weeks

Comparison	p.value	95%CrI
d.CAE.CE	0.41825	
-> direct		-0.53 (-1.1, -0.0024)
-> indirect		-0.17 (-0.90, 0.57)
-> network		-0.55 (-0.98, -0.11)
d.CAE.TCS	0.53775	
-> direct		-0.50 (-1.3, 0.34)
-> indirect		-0.21 (-0.66, 0.24)
-> network		-0.27 (-0.68, 0.12)

FBG

a.Exclude high risk of bias

Comparison	p.value	95%CrI
d.CAE.CE	0.2758	
-> direct		-1.2 (-3., 0.58)
-> indirect		-0.024 (-1.4, 1.4)
-> network		-0.49 (-1.5, 0.59)
d.CAE.CG	0.2392	
-> direct		1.2 (0.31, 2.)
-> indirect		0.0020 (-1.8, 1.8)
-> network		0.90 (0.13, 1.6)
d.CAE.TCS	0.2669	
-> direct		-0.56 (-2.2, 1.1)
-> indirect		0.51 (-0.65, 1.6)
-> network		0.14 (-0.84, 1.1)
d.CG.TCS	0.262	
-> direct		-0.58 (-1.4, 0.21)
-> indirect		-1.7 (-3.5, 0.18)

-> network		-0.76 (-1.5, -0.00067)

b.Exclude interventions less than 12 weeks

Comparison	p.value	95%CrI
d.CAE.CE	0.0123	
-> direct		-1.1 (-2.0, -0.18)
-> indirect		0.98 (-0.30, 2.2)
-> network		-0.39 (-1.2, 0.48)
d.CAE.TCS	0.7836	
-> direct		0.20 (-1.6, 1.9)
-> indirect		0.47 (-0.54, 1.4)
-> network		0.44 (-0.38, 1.2)

2hPG

a.Exclude high risk of bias

Comparison	p.value	95%CrI
d.CAE.CE	0.2614	
-> direct		-2.4 (-5.1, 0.36)
-> indirect		-0.64 (-4.5, 2.2)
-> network		-2. (-5., 0.94)
d.CAE.CG	0.419	
-> direct		1.7 (-0.78, 4.5)
-> indirect		0.47 (-4.2, 5.1)
-> network		1.2 (-1.1, 3.5)
d.CAE.TCS	0.6619	
-> direct		-1.1 (-5.7, 3.3)
-> indirect		-0.15 (-4.6, 4.4)
-> network		-0.66 (-3.3, 2.0)
d.CG.TCS	0.6808	
-> direct		-1.6 (-4.8, 1.7)
-> indirect		-2.5 (-7.9, 2.9)
-> network		-1.8 (-4.2, 0.51)

b.Exclude interventions less than 12 weeks

Comparison	p.value	95%CrI
d.CAE.CE	0.3944	
-> direct		-2.4 (-6.3, 1.4)
-> indirect		-0.52 (-5.9, 4.1)
-> network		-1.8 (-5., 1.4)

6.6.3 SUCRA of sensitivity analyses

HbA1c

a.Exclude high risk of bias

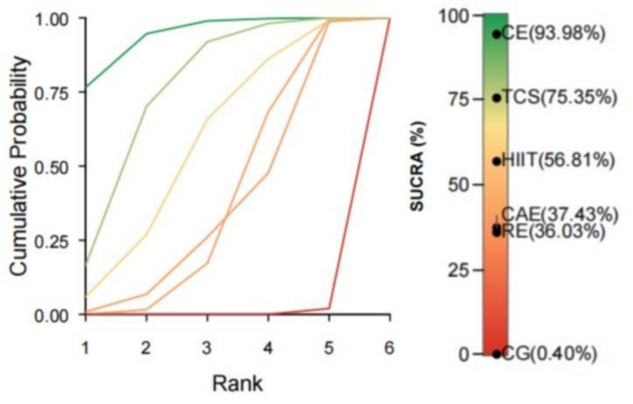

b. Exclude interventions less than 12 weeks

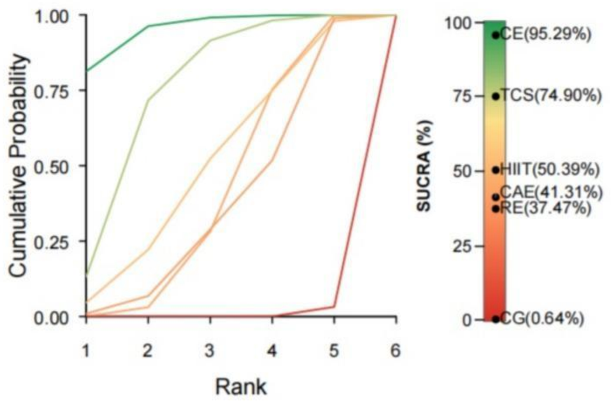

FBG

a.Exclude high risk of bias

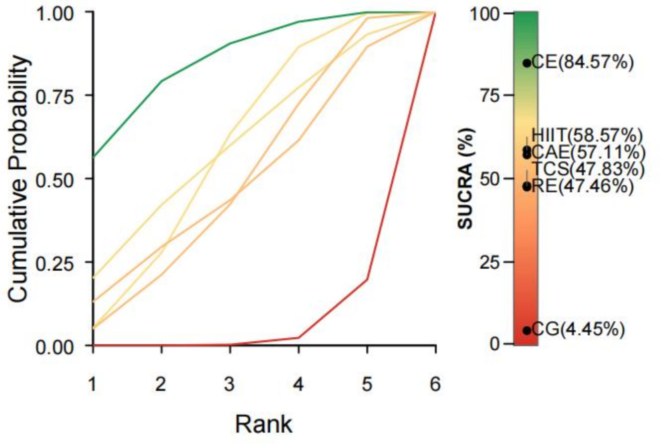

b. Exclude interventions less than 12 weeks

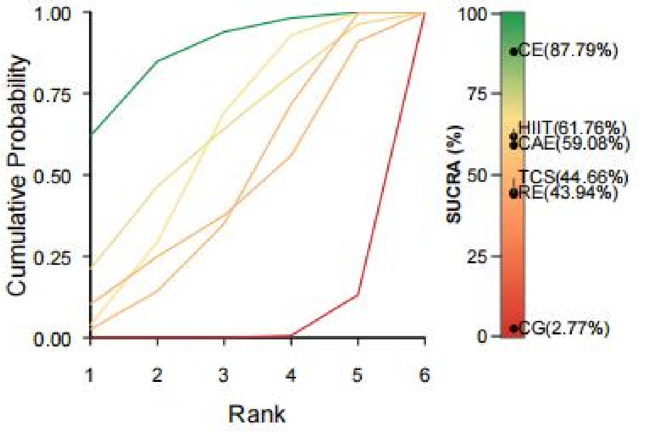

2hPG

a.Exclude high risk of bias

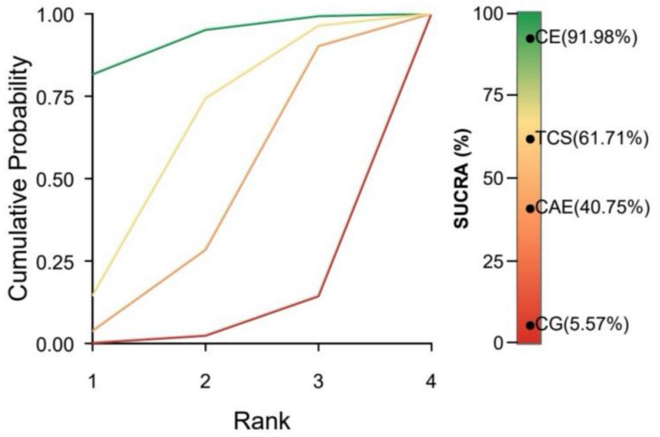

b. Exclude interventions less than 12 weeks

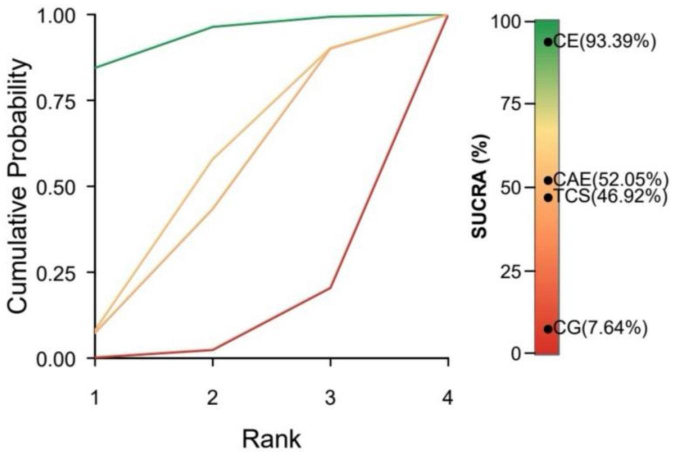

Figure S6. SUCRA of sensitivity analyses. CG, control group. CAE, continuous aerobic exercise. CE, combined aerobic and resistance exercise. RE, resistance exercise. HIIT, high-intensity interval training. TCS, traditional Chinese sports.

6.6.4 Forest plot and league table for sensitivity analyses

HbA1c

a. Exclude high risk of bias

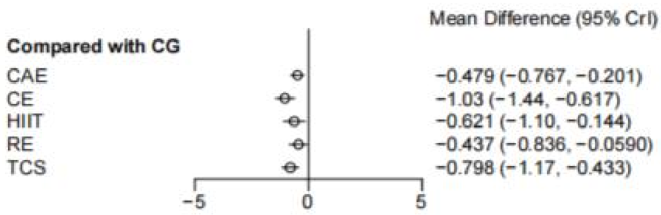

b. Exclude interventions less than 12 weeks

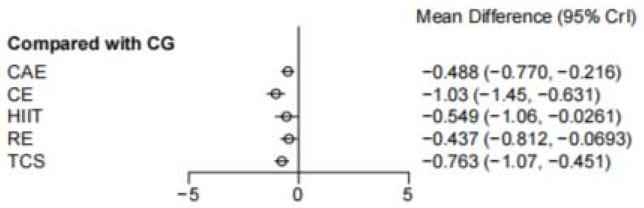

FBG

a. Exclude high risk of bias

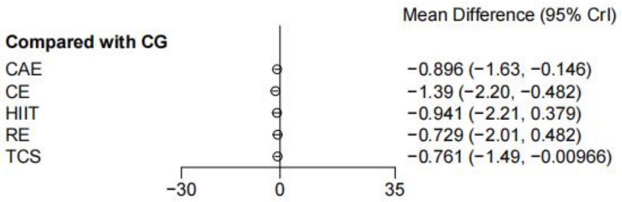

b. Exclude interventions less than 12 weeks

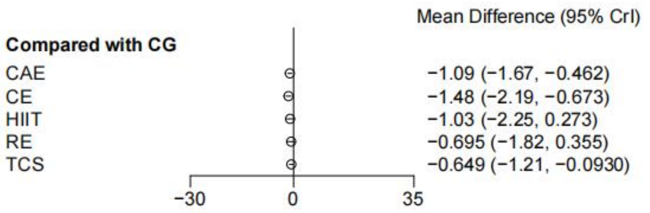

2hPG

a. Exclude high risk of bias

b. Exclude interventions less than 12 weeks

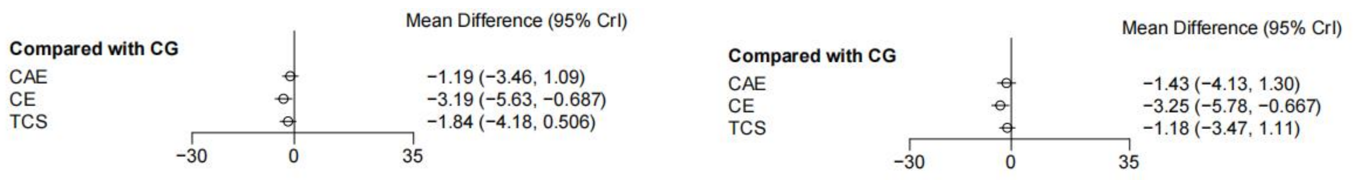

Figure S7. Forest plot of sensitivity analyses. CG, control group. CAE, continuous aerobic exercise. CE, combined aerobic and resistance exercise. RE, resistance exercise. HIIT, high-intensity interval training. TCS, traditional Chinese sports.

Table S8. League table for sensitivity analyses

HbA1c

a.Exclude high risk of bias					
CAE					
0.55(0.10, 0.99)	CE				
0.14(-0.26, 0.53)	-0.41(-1.00, 0.18)	HIIT			
-0.04(-0.52, 0.44)	-0.59(-1.16, -0.03)	-0.18(-0.81, 0.43)	RE		
0.32(-0.10, 0.73)	-0.24(-0.77, 0.31)	0.17(-0.37, 0.75)	0.36(-0.17, 0.90)	TCS	
-0.48(-0.77, -0.20)	-1.03(-1.44, -0.62)	-0.62(-1.10, -0.14)	-0.44(-0.84, -0.06)	-0.80(-1.17, -0.43)	CG
b. Exclude interventions less than 12 weeks					
CAE					
0.55(0.11, 0.98)	CE				
0.06(-0.39, 0.50)	-0.49(-1.11, 0.13)	HIIT			
-0.05(-0.52, 0.41)	-0.60(-1.14, -0.05)	-0.11(-0.74, 0.53)	RE		
0.28(-0.12, 0.67)	-0.27(-0.78, 0.24)	0.21(-0.38, 0.82)	0.33(-0.15, 0.81)	TCS	
-0.49(-0.77, -0.22)	-1.03(-1.45, -0.63)	-0.55(-1.06, -0.03)	-0.44(-0.81, -0.07)	-0.76(-1.07, -0.45)	CG

FBG

a.Exclude high risk of bias					
CAE					
0.49(-0.60, 1.51)	CE				
0.04(-1.03, 1.08)	-0.44(-1.92, 1.06)	HIIT			
-0.17(-1.58, 1.32)	-0.65(-2.10, 0.94)	-0.21(-1.95, 1.63)	RE		
-0.14(-1.08, 0.81)	-0.63(-1.71, 0.52)	-0.19(-1.58, 1.25)	0.02(-1.47, 1.45)	TCS	
-0.90(-1.64, -0.14)	-1.39(-2.21, -0.48)	-0.94(-2.21, 0.38)	-0.73(-2.01, 0.49)	-0.76(-1.49, -0.01)	CG
b. Exclude interventions less than 12 weeks					
CAE					
0.39(-0.48, 1.20)	CE				
-0.06(-1.19, 1.04)	-0.44(-1.84, 0.94)	HIIT			
-0.39(-1.59, 0.92)	-0.78(-2.04, 0.63)	-0.33(-1.94, 1.43)	RE		
-0.44(-1.21, 0.38)	-0.83(-1.72, 0.17)	-0.38(-1.71, 1.02)	-0.05(-1.32, 1.15)	TCS	
-1.09(-1.67, -0.46)	-1.48(-2.19, -0.67)	-1.03(-2.25, 0.27)	-0.70(-1.82, 0.35)	-0.65(-1.21, -0.09)	CG

2hPG

a.Exclude high risk of bias				
CAE				
2.00(-1.00, 4.93)	CE			
0.65(-2.03, 3.32)	-1.34(-4.65, 1.96)	TCS		
-1.19(-3.46, 1.09)	-3.19(-5.63, -0.69)	-1.84(-4.18, 0.51)	CG	
b. Exclude interventions less than 12 weeks				
CAE				
1.82(-1.38, 4.99)	CE			
-0.25(-3.80, 3.29)	-2.06(-5.47, 1.38)	HIIT		
-1.43(-4.13, 1.30)	-3.25(-5.78, -0.67)	-1.18(-3.47, 1.11)	TCS	

Note: CG, control group. CAE, continuous aerobic exercise. CE, combined aerobic and resistance exercise. RE, resistance exercise. HIIT, high-intensity interval training. TCS, traditional Chinese sports.

6.7 Network meta-regression

Network meta-regression analyses of glycated hemoglobin outcome were performed to investigate potential moderating variables and the degree of model fit. The network meta-regression model was fitted in a Bayesian framework using the Markov chain Monte Carlo (MCMC) method in the R statistical package "Gemtc". Four chains were run using a non-informative prior. The number of iterations for each chain was 25,000, with the first 5,000 iterations discarded. Convergence was assessed by visual inspection of trajectory plots and potential scaling factors.

Table S9. Model fit summaries for univariate network meta-regression.

HbA1c

Covariate	HbA1c				
	DIC	pD	Residual Deviance	Shared beta (Median and 95% CrI)	SD
Unadjusted	121.2	55.9	65.3	-	0.34 (0.24, 0.50)
Age	121.9	56.4	65.5	-0.04 (-0.36, 0.31)	0.35 (0.24, 0.51)
Intervention duration (weeks)	122.3	56.2	66.1	0.15 (-0.21, 0.49)	0.33 (0.23, 0.50)
Frequency	121.6	56.1	65.4	-0.15 (-0.48, 0.18)	0.34 (0.23, 0.50)
Publication date	122.2	56.6	65.6	0.01 (-0.41, 0.41)	0.35 (0.24, 0.51)
BMI	99.8	46.4	53.4	0.40 (-0.04, 0.85)	0.34 (0.23, 0.53)
Proportion of man	106.9	49.7	57.2	0.22 (-0.45, 0.87)	0.36 (0.24, 0.54)
Sample Size	121.8	56.4	65.4	-0.05 (-0.36, 0.25)	0.35 (0.24, 0.51)

FBG

Covariate	FBG				
	DIC	pD	Residual Deviance	Shared beta (Median and 95% CrI)	SD
Unadjusted	96.1	45.1	51	-	0.67 (0.46, 1.02)
Age	96.3	45.3	51	0.25 (-0.49, 0.98)	0.68 (0.46, 1.05)
Intervention duration (weeks)	96.6	45.1	51.6	-0.44 (-1.24, 0.32)	0.65 (0.43, 1.01)
Frequency	96.7	45.6	51.1	-0.05 (-0.99, 0.87)	0.69 (0.47, 1.07)
Publication date	97.1	45.5	51.6	0.23 (-0.71, 1.02)	0.67 (0.45, 1.05)
BMI	77.7	35.2	42.5	0.18 (-1.08, 1.45)	0.56 (0.27, 1.07)
Proportion of man	85.9	40.1	45.7	-0.47 (-1.65, 0.78)	0.71 (0.44, 1.17)
Sample Size	96.4	45.5	50.9	-0.05 (-0.67, 0.60)	0.70 (0.47, 1.08)

2hPG

Covariate	2hPG				
	DIC	pD	Residual Deviance	Shared beta (Median and 95% CrI)	SD
Unadjusted	32.6	16	16.6	-	1.41 (0.69, 3.37)
Age	32.4	16	16.4	1.95 (-1.32, 4.79)	1.12 (0.49, 3.08)
Intervention duration (weeks)	32.8	16.2	16.7	-1.08 (-5.57, 3.18)	1.48 (0.68, 3.67)
Frequency	32.7	16.1	16.5	-0.53 (-3.56, 2.60)	1.57 (0.74, 3.81)
Publication date	33	16.2	16.7	1.25 (-2.71, 4.92)	1.42 (0.67, 3.56)
BMI	24.8	12.3	12.5	3.09 (-2.82, 11.57)	1.45 (0.47, 4.24)
Proportion of man	32.4	16.1	16.4	1.77 (-1.23, 4.48)	1.20 (0.56, 3.25)
Sample Size	32.7	16.1	16.6	-0.54 (-4.17, 2.56)	1.57 (0.74, 3.84)

Note: CrI, credible interval. DIC, deviance information criterion. SD, standard deviation.

6.8 Summary grading of evidence

Table S10. Evidence assessment for all studies
HbA1c

Comparison	Number of studies	Within-study bias	Reporting bias	Indirectness	Imprecision	Heterogeneity	Incoherence	Confidence rating
CAE:CE	3	Some concerns	Some concerns	No concerns	Some concerns	Some concerns	No concerns	Low
CAE:CG	10	Some concerns	Some concerns	No concerns	No concerns	Major concerns	No concerns	Very low
CAE:HIIT	5	No concerns	Some concerns	Some concerns	Some concerns	Some concerns	No concerns	Low
CAE:TCS	2	Some concerns	Some concerns	No concerns	Some concerns	Some concerns	No concerns	Low
CE:CG	6	Some concerns	Some concerns	No concerns	No concerns	Major concerns	No concerns	Very low
CG:RE	6	Some concerns	Some concerns	No concerns	Some concerns	Some concerns	No concerns	Low
CG:TCS	8	Some concerns	Some concerns	No concerns	No concerns	No concerns	No concerns	Moderate
CAE:RE	0	Some concerns	Some concerns	No concerns	Some concerns	Some concerns	No concerns	Low
CE:HIIT	0	Some concerns	Some concerns	No concerns	Major concerns	No concerns	No concerns	Very low
CE:RE	0	Some concerns	Some concerns	No concerns	Some concerns	Some concerns	No concerns	Low
CE:TCS	0	Some concerns	Some concerns	No concerns	Some concerns	Some concerns	No concerns	Low
CG:HIIT	0	Some concerns	Some concerns	No concerns	Some concerns	Some concerns	No concerns	Low

HIIT:RE	0	Some concerns	Some concerns	No concerns	Some concerns	Some concerns	No concerns	Low
HIIT:TCS	0	Some concerns	Some concerns	No concerns	Some concerns	No concerns	No concerns	Low
RE:TCS	0	Some concerns	Some concerns	No concerns	Some concerns	Some concerns	No concerns	Low

FBG

Comparison	Number of studies	Within-study bias	Reporting bias	Indirectness	Imprecision	Heterogeneity	Incoherence	Confidence rating
CAE:CE	2	Some concerns	Some concerns	No concerns	Some concerns	Some concerns	No concerns	Low
CAE:CG	7	Some concerns	Some concerns	No concerns	No concerns	Major concerns	Some concerns	Very low
CAE:HIIT	4	Some concerns	Some concerns	Some concerns	Some concerns	No concerns	No concerns	Low
CAE:TCS	2	Some concerns	Some concerns	No concerns	Some concerns	Some concerns	No concerns	Low
CE:CG	6	Some concerns	Some concerns	No concerns	No concerns	Some concerns	No concerns	Low
CG:RE	2	Some concerns	Some concerns	No concerns	Some concerns	Some concerns	No concerns	Low
CG:TCS	8	Some concerns	Some concerns	No concerns	No concerns	Some concerns	No concerns	Low
CAE:RE	0	Some concerns	Some concerns	No concerns	Major concerns	No concerns	No concerns	Very low
CE:HIIT	0	Some concerns	Some concerns	No concerns	Some concerns	No concerns	No concerns	Low
CE:RE	0	Some concerns	Some concerns	No concerns	Some concerns	No concerns	No concerns	Low
CE:TCS	0	Some concerns	Some concerns	No concerns	Some concerns	Some concerns	No concerns	Low

CG:HIIT	0	Some concerns	Some concerns	No concerns	Some concerns	Some concerns	No concerns	Low
HIIT:RE	0	Some concerns	Some concerns	No concerns	Major concerns	No concerns	No concerns	Very low
HIIT:TCS	0	Some concerns	Some concerns	No concerns	Some concerns	No concerns	No concerns	Low
RE:TCS	0	Some concerns	Some concerns	No concerns	Major concerns	No concerns	No concerns	Very low

2hPG

Comparison	Number of studies	Within-study bias	Reporting bias	Indirectness	Imprecision	Heterogeneity	Incoherence	Confidence rating
CAE:CE	1	Some concerns	Some concerns	No concerns	No concerns	Major concerns	No concerns	Very low
CAE:CG	2	Some concerns	Some concerns	No concerns	Some concerns	Some concerns	Some concerns	Low
CAE:TCS	1	Some concerns	Some concerns	No concerns	Some concerns	Some concerns	No concerns	Low
CE:CG	3	Some concerns	Some concerns	No concerns	No concerns	Some concerns	No concerns	Low
CG:TCS	3	Some concerns	Some concerns	No concerns	No concerns	Major concerns	No concerns	Very low
CE:TCS	0	Some concerns	Some concerns	No concerns	Some concerns	Some concerns	Major concerns	Very low

7. Dose-response network meta-analyses

7.1 Key assumptions for network meta-analysis

7.1.1 Network connectivity

Network connectivity is a key assumption in NMA; insufficient connectivity may reduce statistical power and lead to misleading estimates (i.e., due to lack of direct comparisons), may lead to low statistical power and misleading results. Our study assessed network connectivity at the treatment-modality and dose levels and found no evidence of disconnected networks (Figure S8 and Figure S9)

HbA1c

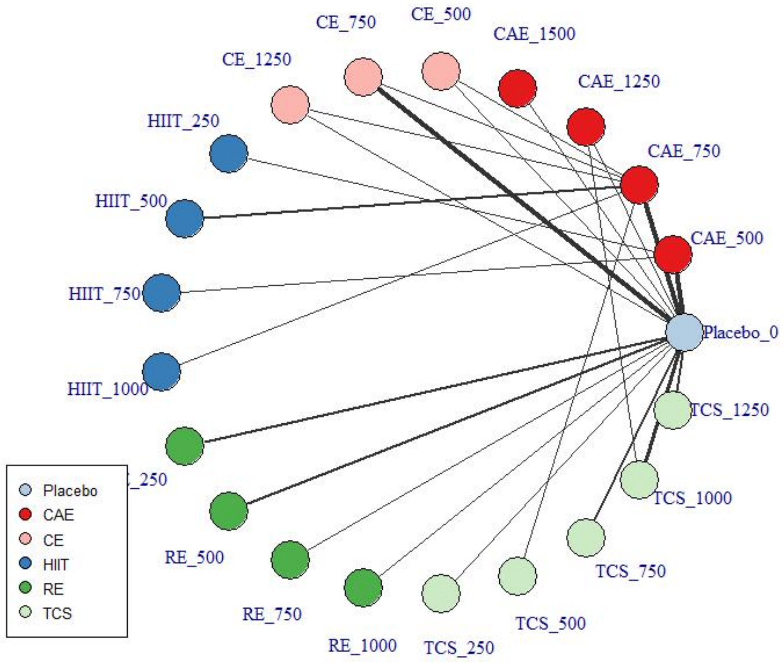

FBG

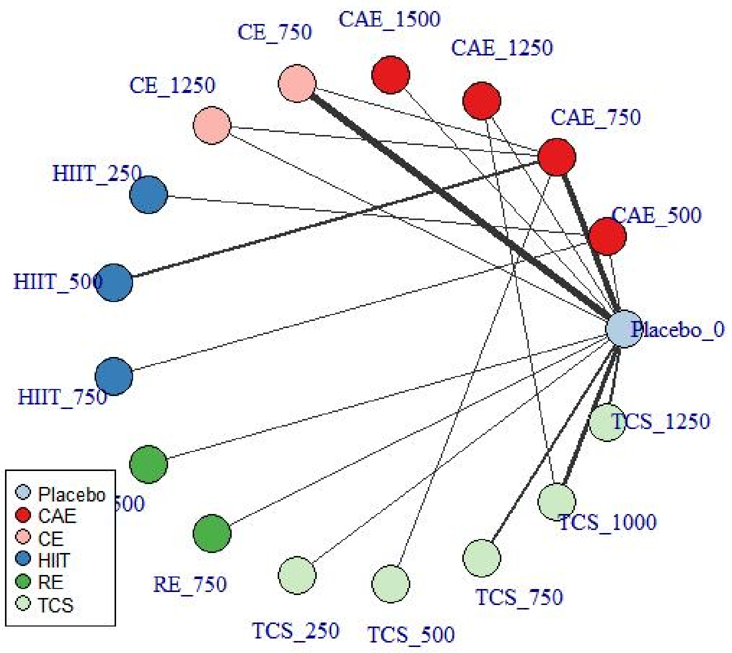

Figure S8. Treatment-level network. The first label indicates the specific intervention and the second one is the corresponding dose of that intervention. Placebo, control group. CAE, continuous aerobic exercise. CE, combined aerobic and resistance exercise. RE, resistance exercise. HIIT, high-intensity interval training. TCS, traditional Chinese sports.

HbA1c

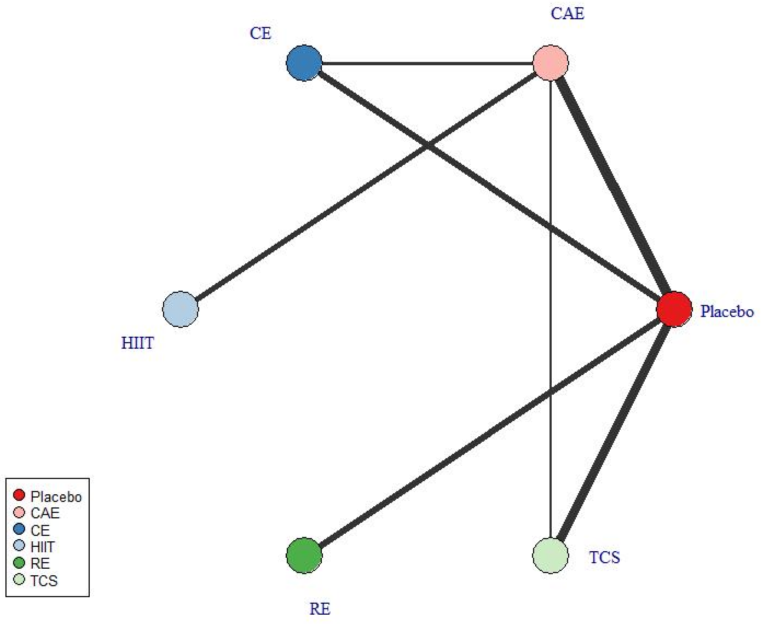

FBG

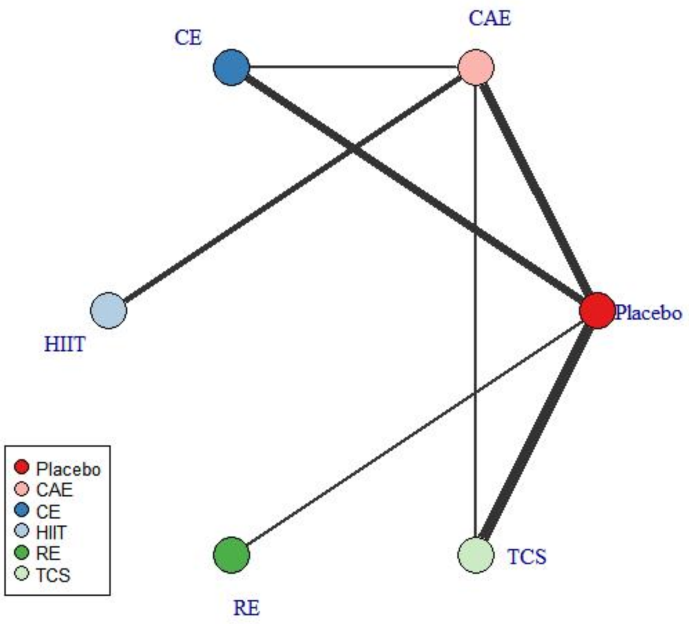

Figure S9. Agent-level network. The first value indicates the specific intervention and the second one is the corresponding dose of that intervention. Placebo, control group. CAE, continuous aerobic exercise. CE, combined aerobic and resistance exercise. RE, resistance exercise. HIIT, high-intensity interval training. TCS, traditional Chinese sports.

7.1.2 Data consistency

We assessed consistency by comparing the consistency model with the UME model. In practice, we checked whether the bias, the number of estimated parameters in the network, and the Deviance Information Criterion (DIC) metric were similar for both models, indicating a good fit. The comparison of these parameters showed a good agreement between the different models (Table S11).

Table S11. Consistent and UME models fit comparison.
HbA1c

Model	pD	Deviance	Residual deviance	DIC	SD
Consistent	56.1	-48.961	65.037	6.8	0.394
UME	56.1	-49.000	64.998	7.2	0.375

FBG

Model	pD	Deviance	Residual deviance	DIC	SD
Consistent	44.0	9.237	49.183	52.9	0.704
UME	44.2	9.261	49.207	53.6	0.687

7.1.3 Network transitivity

Table S12. Node-splitting analysis of consistency
HbA1c

Comparison	p-value	Median	2.50%	97.50%
TCS_500 vs CAE_750	0.196			
-> direct		-0.529	-1.25	0.157
-> indirect		0.021	-0.219	0.259
-> MBNMA		-0.042	-0.273	0.205
HIIT_1000 vs CAE_750	0.889			
-> direct		-0.371	-1.116	0.369
-> indirect		-0.292	-1.161	0.56
-> MBNMA		-0.357	-0.909	0.224
HIIT_500 vs CAE_750	0.441			
-> direct		-0.269	-0.926	0.39
-> indirect		0.072	-0.262	0.398
-> MBNMA		0.014	-0.275	0.317
HIIT_750 vs CAE_500	0.437			
-> direct		0.096	-0.774	0.967
-> indirect		-0.408	-0.881	0.053
-> MBNMA		-0.3	-0.725	0.135
HIIT_250 vs CAE_500	0.381			
-> direct		0.105	-0.684	0.873
-> indirect		0.068	-0.094	0.24
-> MBNMA		0.071	-0.082	0.236
TCS_1250 vs Placebo_0	0.623			
-> direct		-0.879	-1.582	-0.152
-> indirect		-1.143	-1.619	-0.694
-> MBNMA		-1.059	-1.435	-0.677
TCS_1000 vs Placebo_0	0.889			
-> direct		-0.82	-1.285	-0.375
-> indirect		-0.887	-1.295	-0.442
-> MBNMA		-0.847	-1.148	-0.542
TCS_750 vs Placebo_0	0.663			

-> direct		-0.607	-1.159	-0.069
-> indirect		-0.646	-0.903	-0.373
-> MBNMA		-0.635	-0.861	-0.406
TCS_250 vs Placebo_0	0.079			
-> direct		-0.949	-2.02	0.108
-> indirect		-0.208	-0.285	-0.13
-> MBNMA		-0.212	-0.287	-0.135
RE_1000 vs Placebo_0	0.59			
-> direct		-1.148	-2.578	0.248
-> indirect		-0.743	-1.492	-0.043
-> MBNMA		-0.821	-1.466	-0.198
RE_750 vs Placebo_0	0.787			
-> direct		-0.511	-1.286	0.24
-> indirect		-0.679	-1.29	-0.084
-> MBNMA		-0.616	-1.1	-0.149
RE_500 vs Placebo_0	0.874			
-> direct		-0.393	-0.889	0.151
-> indirect		-0.419	-0.827	-0.033
-> MBNMA		-0.411	-0.733	-0.099
RE_250 vs Placebo_0	0.358			
-> direct		-0.254	-1.047	0.528
-> indirect		-0.202	-0.361	-0.051
-> MBNMA		-0.205	-0.367	-0.05
CE_1250 vs Placebo_0	0.606			
-> direct		-1.886	-2.76	-0.996
-> indirect		-1.446	-2.224	-0.674
-> MBNMA		-1.554	-2.154	-0.987
CE_750 vs Placebo_0	0.813			
-> direct		-0.896	-1.407	-0.408
-> indirect		-1.012	-1.504	-0.535
-> MBNMA		-0.932	-1.292	-0.592
CE_500 vs Placebo_0	0.397			
-> direct		-0.809	-1.773	0.204
-> indirect		-0.614	-0.861	-0.365
-> MBNMA		-0.622	-0.862	-0.395
CAE_1500 vs Placebo_0	0.829			
-> direct		-0.795	-1.555	-0.059
-> indirect		-0.751	-1.287	-0.246
-> MBNMA		-0.768	-1.186	-0.363
CAE_1250 vs Placebo_0	0.667			
-> direct		-0.698	-1.526	0.066
-> indirect		-0.612	-0.999	-0.224
-> MBNMA		-0.64	-0.988	-0.302
CAE_750 vs Placebo_0	0.245			

-> direct		-0.704	-1.169	-0.239
-> indirect		-0.299	-0.536	-0.069
-> MBNMA		-0.384	-0.593	-0.181
CAE_500 vs Placebo_0	0.474			
-> direct		-0.367	-0.783	0.038
-> indirect		-0.237	-0.385	-0.088
-> MBNMA		-0.256	-0.395	-0.121

FBG

Comparison	p-value	Median	2.50%	97.50%
TCS_500 vs CAE_750	0.232			
-> direct		-0.544	-1.929	0.806
-> indirect		0.458	-0.054	0.936
-> MBNMA		0.338	-0.134	0.787
HIIT_500 vs CAE_750	0.764			
-> direct		0.333	-1.16	1.865
-> indirect		-0.067	-1.315	1.166
-> MBNMA		0.087	-0.868	1.054
HIIT_750 vs CAE_500	0.887			
-> direct		-0.603	-2.622	1.33
-> indirect		-0.306	-2.499	1.784
-> MBNMA		-0.462	-1.913	0.974
HIIT_250 vs CAE_500	0.47			
-> direct		-0.1	-1.843	1.714
-> indirect		0.181	-0.329	0.684
-> MBNMA		0.162	-0.332	0.655
TCS_1250 vs Placebo_0	0.881			
-> direct		-0.839	-2.007	0.282
-> indirect		-0.972	-1.908	-0.017
-> MBNMA		-0.92	-1.65	-0.202
TCS_1000 vs Placebo_0	0.837			
-> direct		-0.672	-1.604	0.207
-> indirect		-0.811	-1.6	-0.026
-> MBNMA		-0.736	-1.32	-0.161
TCS_750 vs Placebo_0	0.618			
-> direct		-0.454	-1.558	0.622
-> indirect		-0.577	-1.063	-0.109
-> MBNMA		-0.552	-0.99	-0.121
TCS_250 vs Placebo_0	0.116			
-> direct		-1.038	-2.894	0.946
-> indirect		-0.182	-0.327	-0.034
-> MBNMA		-0.184	-0.33	-0.04
RE_750 vs Placebo_0	0.456			
-> direct		-1.594	-3.384	0.117
-> indirect		-0.191	-2.319	2.006

-> MBNMA		-1.022	-2.422	0.285
RE_500 vs Placebo_0	0.438			
-> direct		-0.078	-1.535	1.326
-> indirect		-1.072	-2.199	0.007
-> MBNMA		-0.681	-1.614	0.19
CE_1250 vs Placebo_0	0.684			
-> direct		-2.36	-3.88	-0.911
-> indirect		-1.762	-3.167	-0.213
-> MBNMA		-1.946	-2.887	-0.887
CE_750 vs Placebo_0	0.758			
-> direct		-0.984	-1.893	-0.092
-> indirect		-1.254	-2.107	-0.389
-> MBNMA		-1.167	-1.732	-0.532
CAE_1500 vs Placebo_0	0.565			
-> direct		-0.96	-2.399	0.522
-> indirect		-1.658	-2.688	-0.557
-> MBNMA		-1.415	-2.281	-0.564
CAE_1250 vs Placebo_0	0.599			
-> direct		-1.507	-3.1	0.056
-> indirect		-1.085	-1.871	-0.257
-> MBNMA		-1.179	-1.901	-0.47
CAE_750 vs Placebo_0	0.146			
-> direct		-1.317	-2.003	-0.594
-> indirect		-0.463	-0.912	-0.021
-> MBNMA		-0.708	-1.14	-0.282
CAE_500 vs Placebo_0	0.163			
-> direct		-1.508	-4.254	1.225
-> indirect		-0.456	-0.726	-0.17
-> MBNMA		-0.472	-0.76	-0.188

Note: CrI, credible interval. CG, control group. CAE, continuous aerobic exercise. CE, combined aerobic and resistance exercise. RE, resistance exercise. HIIT, high-intensity interval training. TCS, traditional Chinese sports.

Figure S10. Node-splitting analysis (density plot). The value of title is the corresponding dose of that agent. CrI, credible interval. CG, control group. CAE, continuous aerobic exercise. CE, combined aerobic and resistance exercise. RE, resistance exercise. HIIT, high-intensity interval training. TCS, traditional Chinese sports.

HbA1c

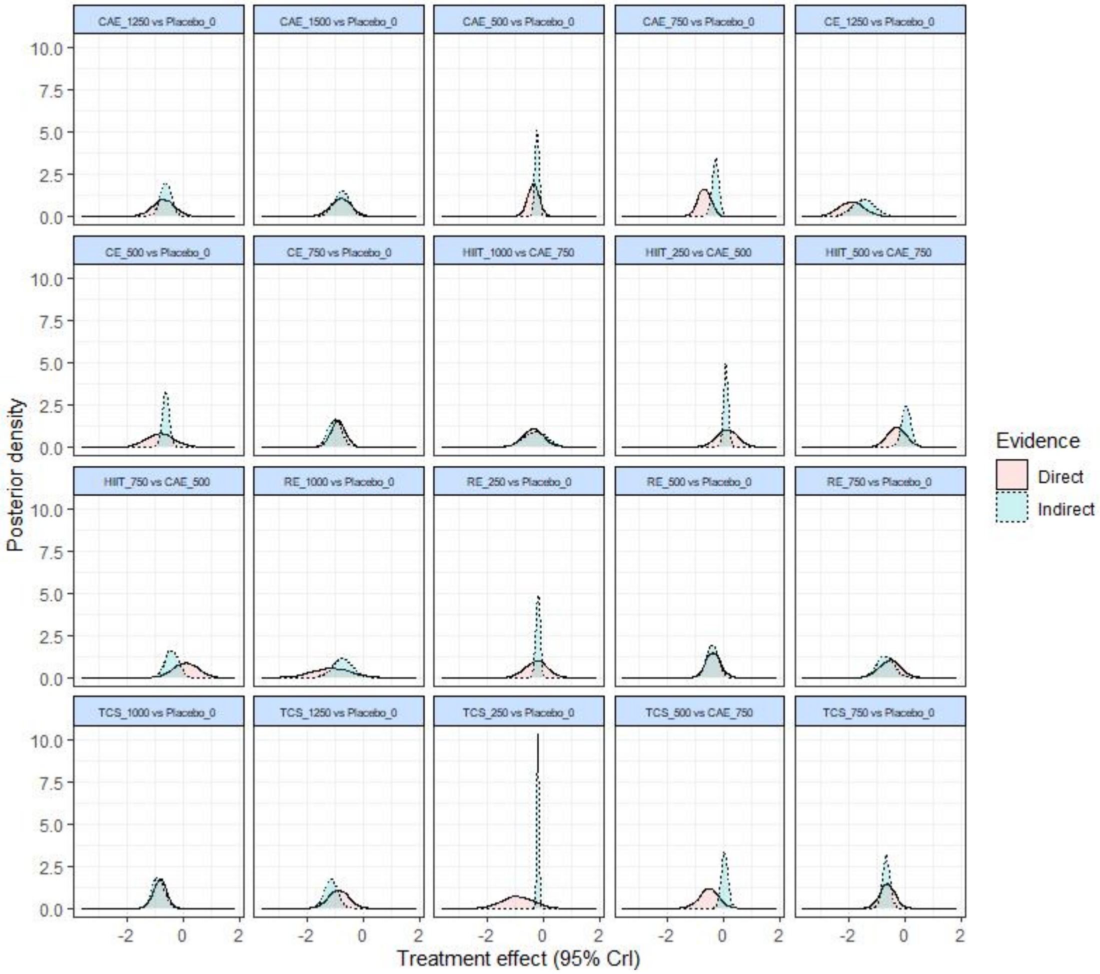

FBG

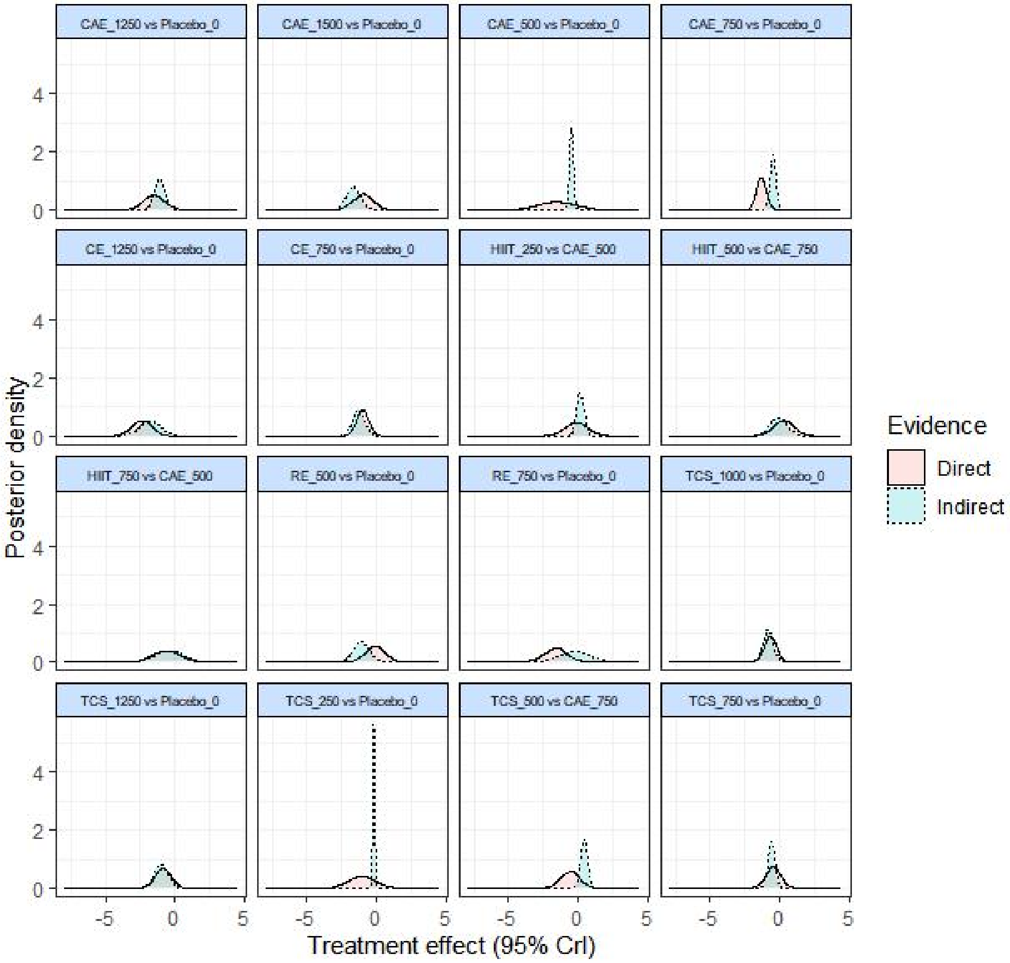

7.2 Models selection

7.2.1 Nonlinear functions and model-fit comparison

A meta-analysis (i.e., a "split" NMA) of the different doses of physical activity as separate and unrelated treatments was performed. This step helps determine which function is more appropriate for the data and should be used in a model-based network meta-analysis (MBNMA). Figure S11 and Figure S12 show the different responses of each dose to overall and different exercise modalities, respectively (MD).

HbA1c

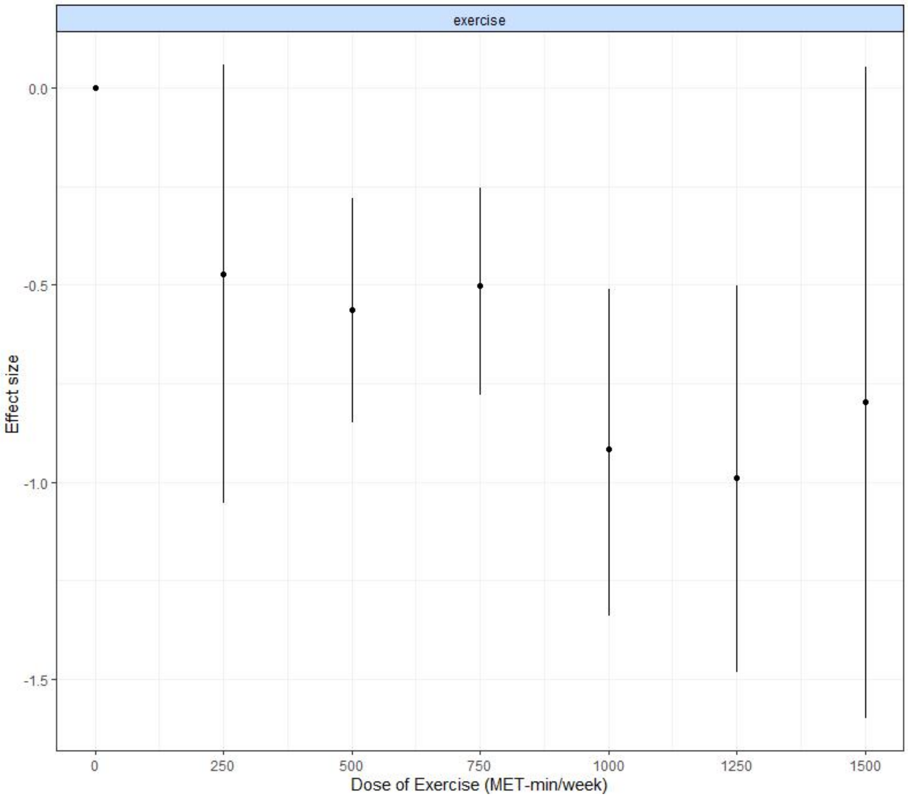

FBG

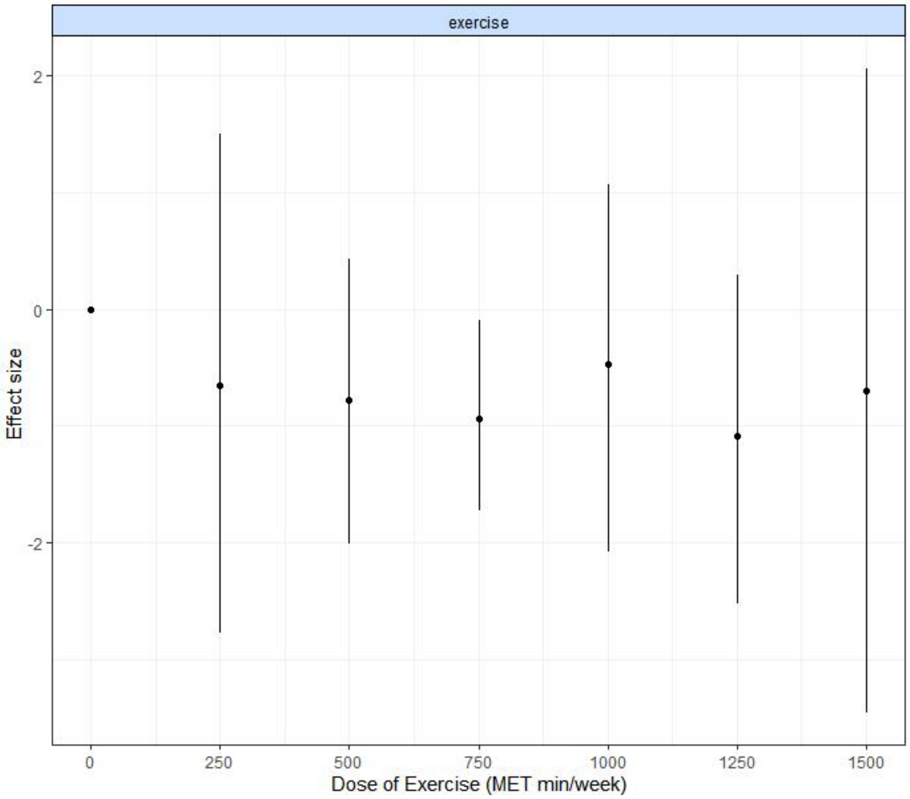

Figure S11. "Split" NMA of overall exercise.

HbA1c

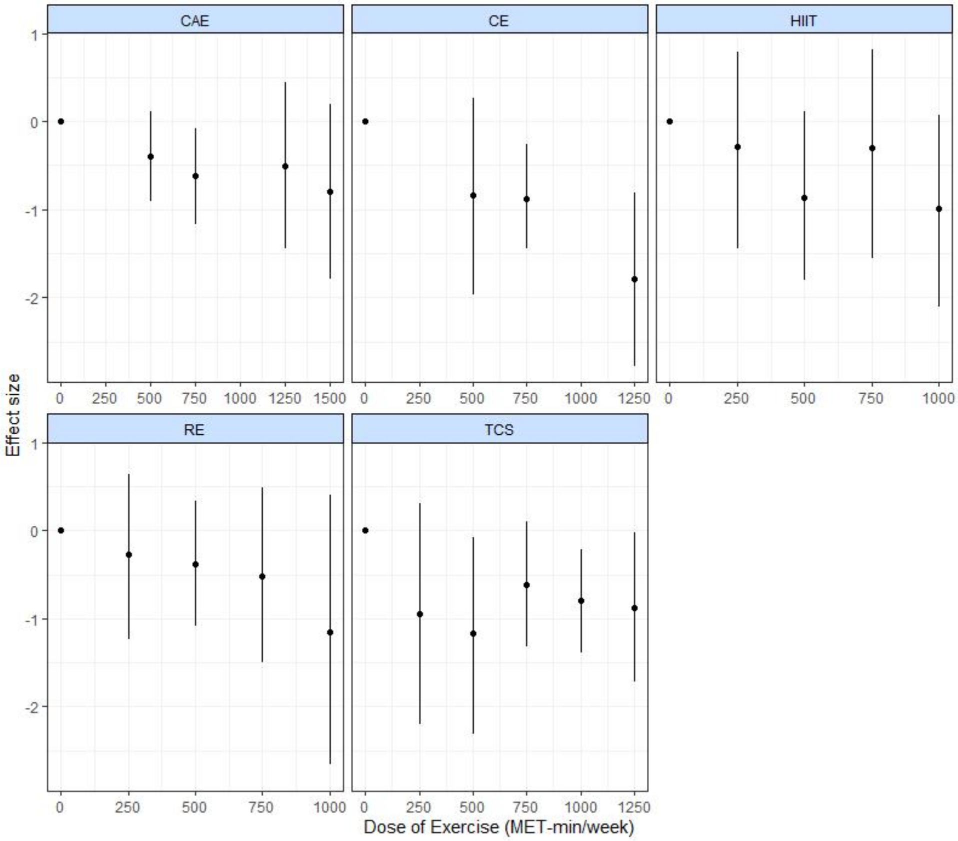

CAE
CE
HIIT
RE
TCS
Effect size
Dose of Exercise (MET-min/week)

FBG

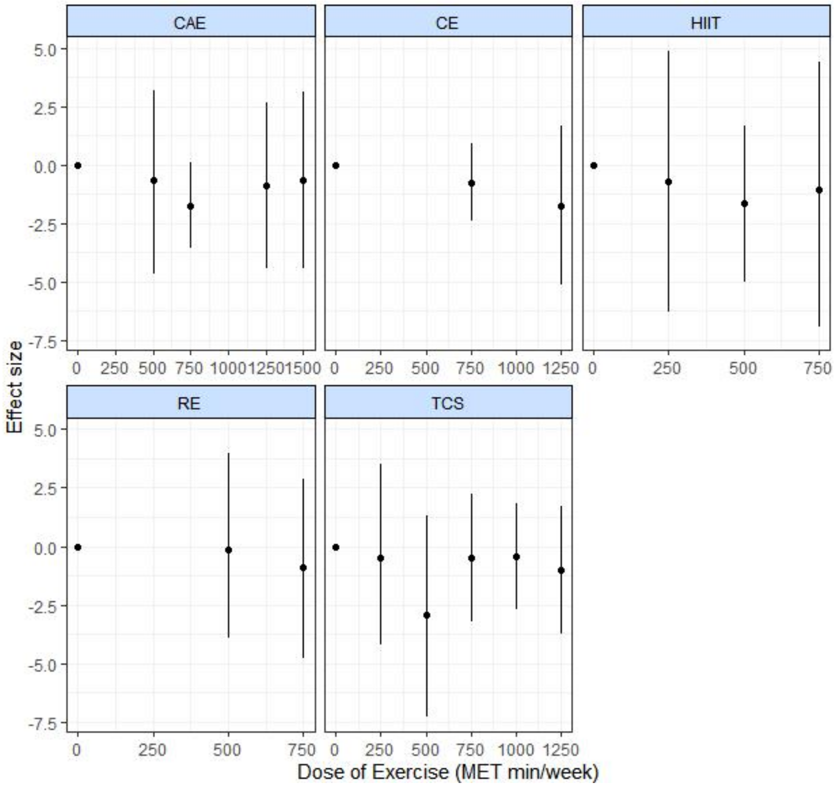

CAE
CE
HIIT
RE
TCS
Effect size
Dose of Exercise (MET min/week)

Figure S12. "Split" NMA of different exercise agents. CG, control group. CAE, continuous aerobic exercise. CE, combined aerobic and resistance exercise. RE, resistance exercise. HIIT, high-intensity interval training. TCS, traditional Chinese sports.

7.2.2 Models selection

In these data, the quadratic model showed the best fit and was therefore used in subsequent analyses.
Table S13. Model-fit comparison

HbA1c

Model	DIC	SD	Deviance	Residual deviance	pD
Emax (common treatment effects)	122.1	NA	85.397	199.395	37.1
Restricted cubic spline (common treatment effects; 3 knots)	119.8	NA	68.241	182.239	51.3
Restricted cubic spline (random treatment effects; 3 knots)	14.4	0.443 (0.27,0.72)	-47.667	66.331	62.4
Non-parametric monotonically up (common treatment effects)	420.6	NA	386.813	500.811	33.7
Quadratic (2nd degree polynomial, common treatment effects)	126.6	NA	84.800	198.798	42.0
Quadratic (2nd degree polynomial, random treatment effects)	9.0	0.368 (0.25,0.54)	-48.685	65.314	58.0

FBG

Model	DIC	SD	Deviance	Residual deviance	pD
Emax (common treatment effects)	384.9	NA	354.940	394.886	29.9

Restricted cubic spline (common treatment effects; 3 knots)	173.7	NA	133.808	173.754	40.0
Restricted cubic spline (random treatment effects; 3 knots)	60.6	0.861 (0.49,1.49)	12.005	51.951	48.9
Non-parametric monotonically up (common treatment effects)	1901.1	NA	1874.404	1914.350	26.2
Quadratic (2nd degree polynomial, common treatment effects)	328.4	NA	293.123	333.069	35.1
Quadratic (2nd degree polynomial, random treatment effects)	57.5	0.764 (0.49,1.17)	10.821	50.767	46.8

In addition to the model fit index, a deviation plot showing the contribution of each data point to the residuals can also help to confirm the robustness of the model selection. The contribution of each data point to the posterior mean bias should be around 1, which indicates a good model fit. Deviation plots for overall (Figure S13) treatment effects (Figure S14) confirm the robustness of our model selection.

HbA1c

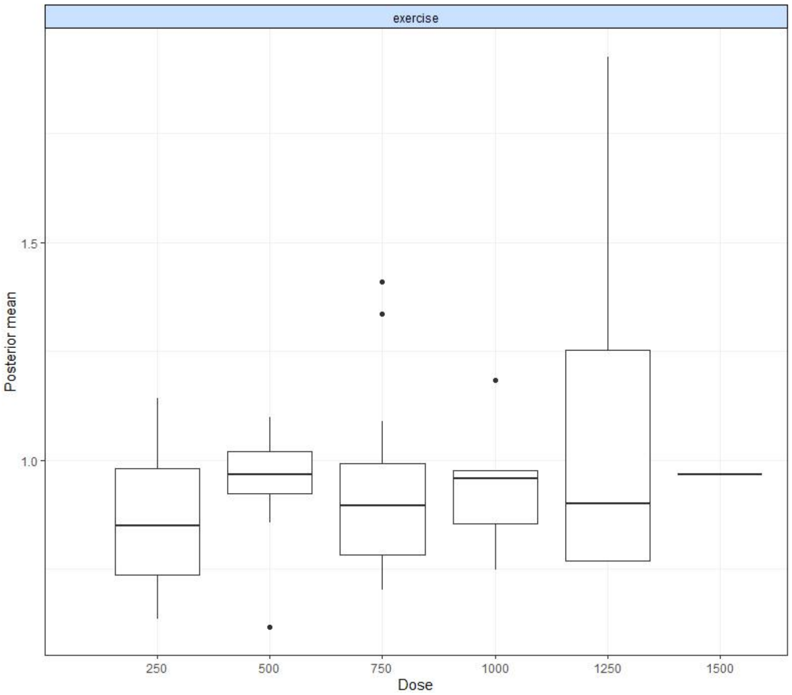

FBG

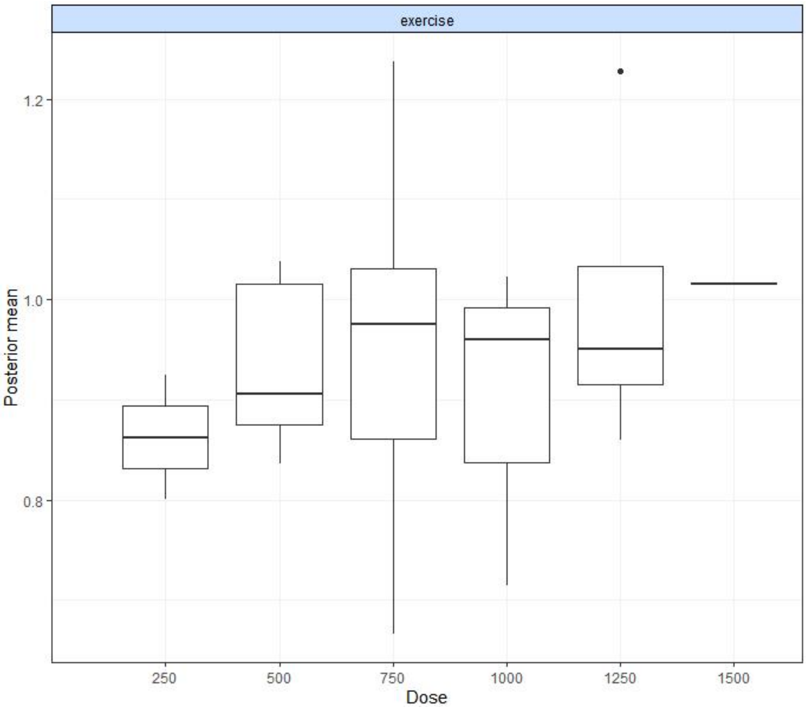

Figure S13. Deviance plot at overall exercise

HbA1c

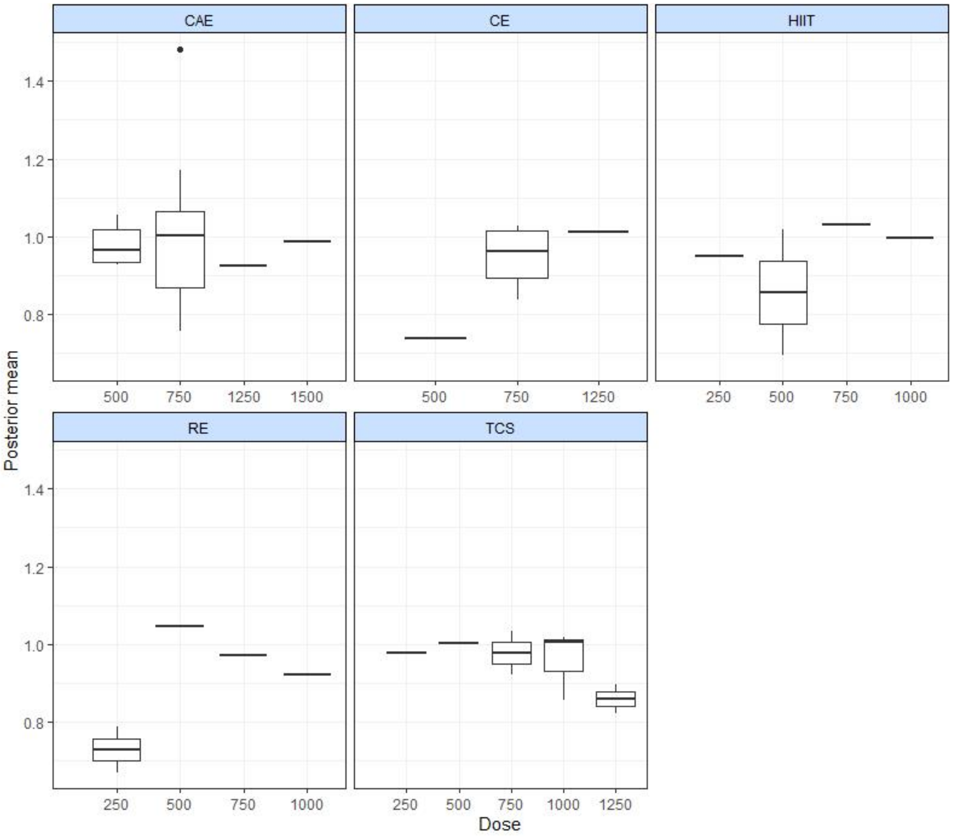
CAE
CE
HIIT
RE
TCS
Posterior mean
Dose
1.4
1.2
1.0
0.8
500
750
1250
1500
250
1000

FBG

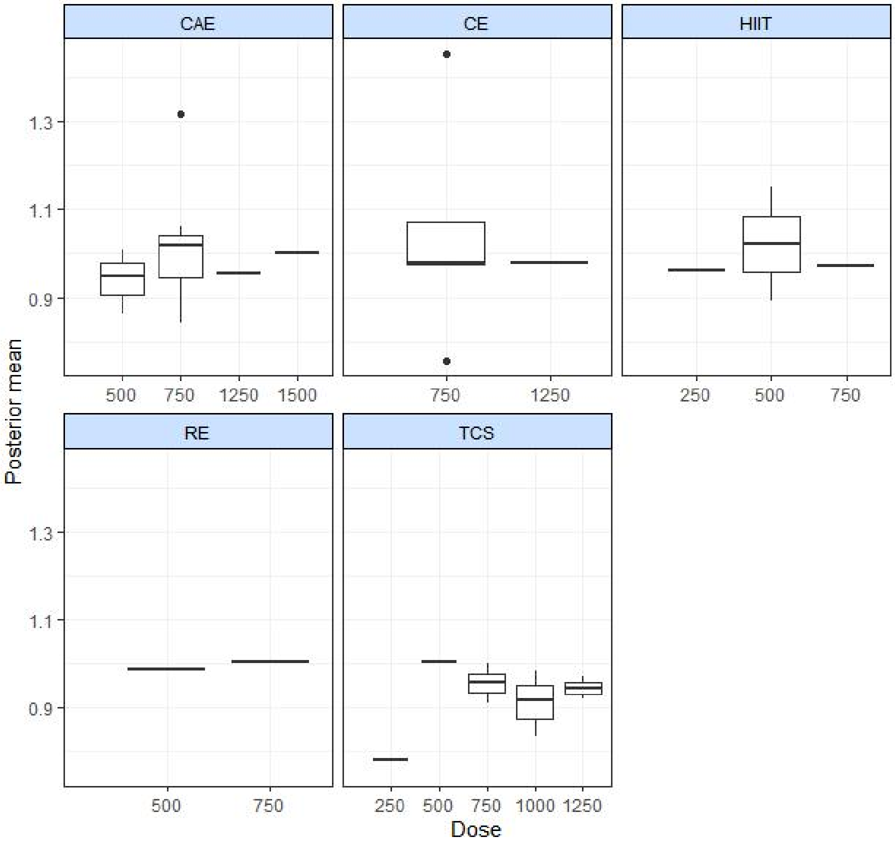
CAE
CE
HIIT
RE
TCS
Posterior mean
Dose
1.3
1.1
0.9
500
750
1250
1500
250
1000

Figure S14. Deviance plots at treatment-level. CG, control group. CAE, continuous aerobic exercise. CE, combined aerobic and resistance exercise. RE, resistance exercise. HIIT, high-intensity interval training. TCS, traditional Chinese sports.

We further generated fit plots to assess model fit. The fit values are plotted as connecting lines, and the observations in the original dataset are plotted as points. These plots were used to assess whether the model fitted the data adequately.

HbA1c

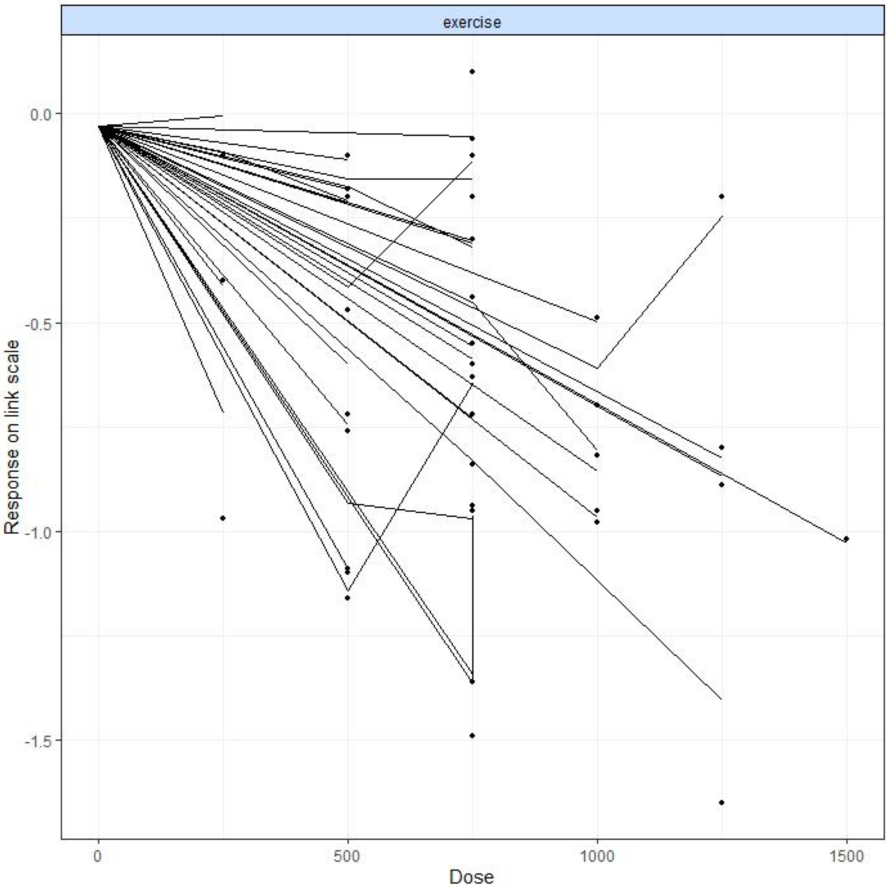

FBG

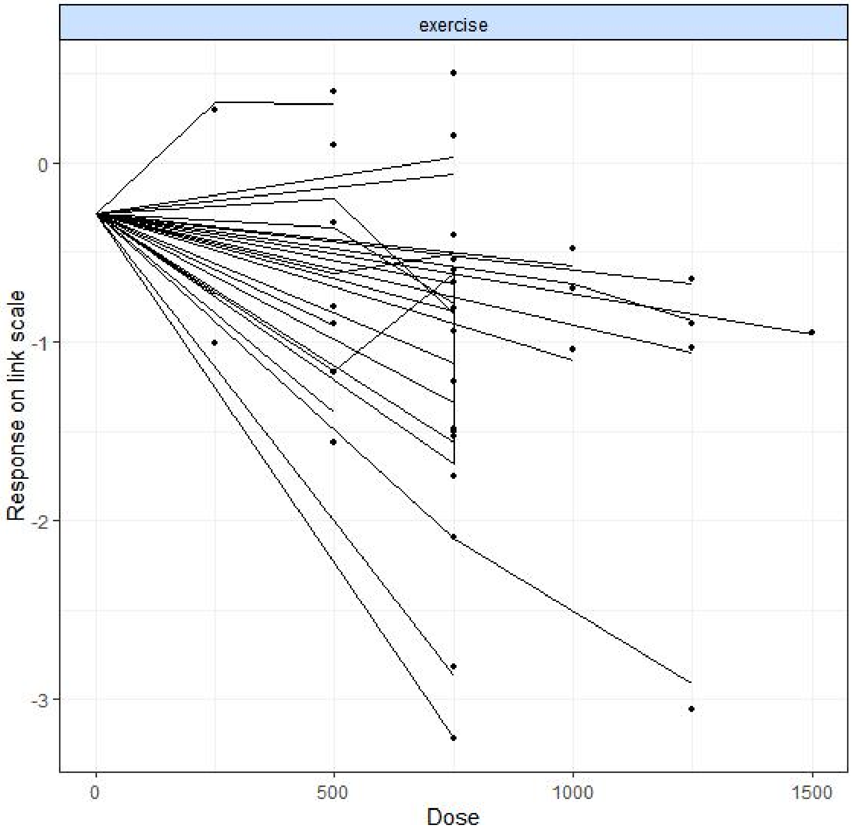

Figure S15. Fit plots at the overall exercise level.

HbA1c

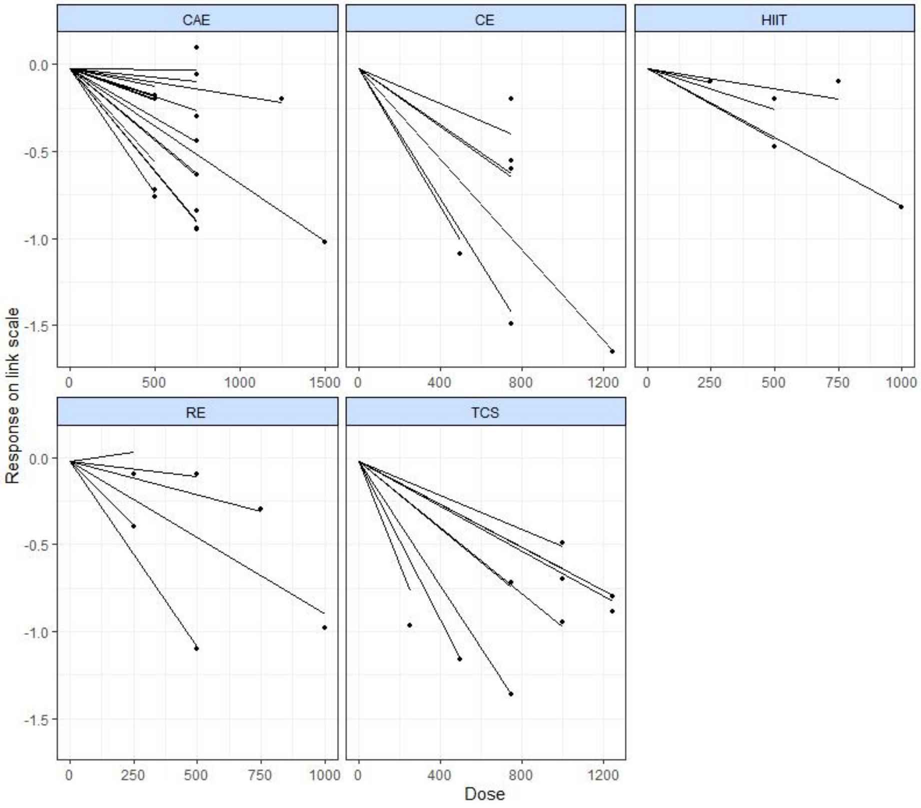

FBG

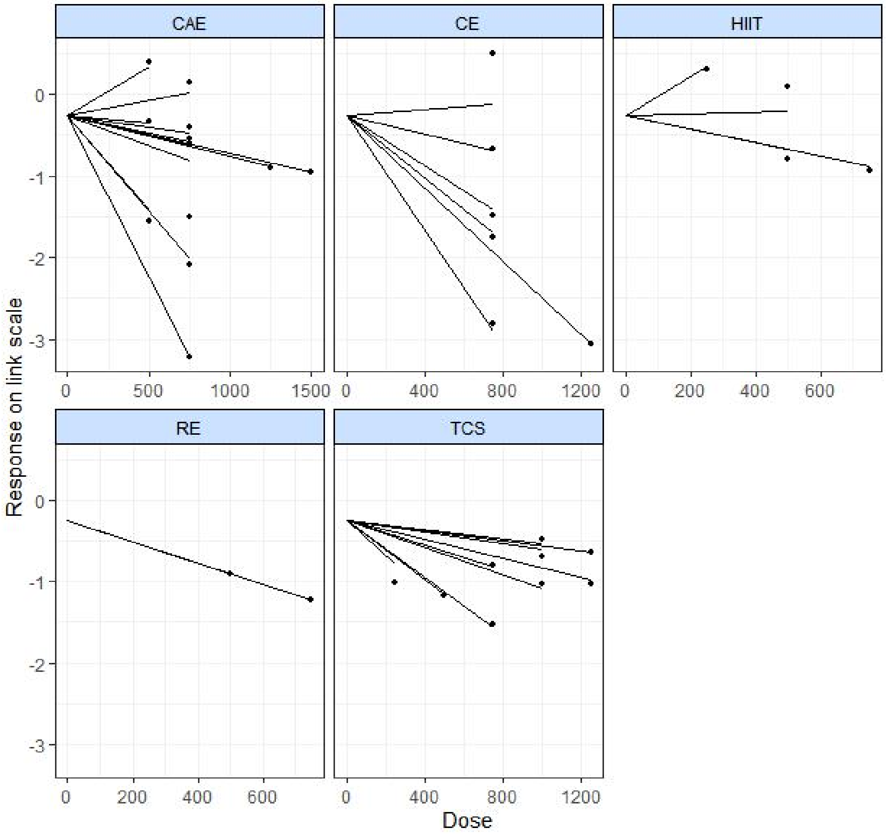

Figure S16. Fit plots at agent-level. CG, control group. CAE, continuous aerobic exercise. CE, combined aerobic and resistance exercise. RE, resistance exercise. HIIT, high-intensity interval training. TCS, traditional Chinese sports.

7.3 Dose-response relationships

7.3.1 Dose-response relationship between exercise dose and glycemic control

HbA1c

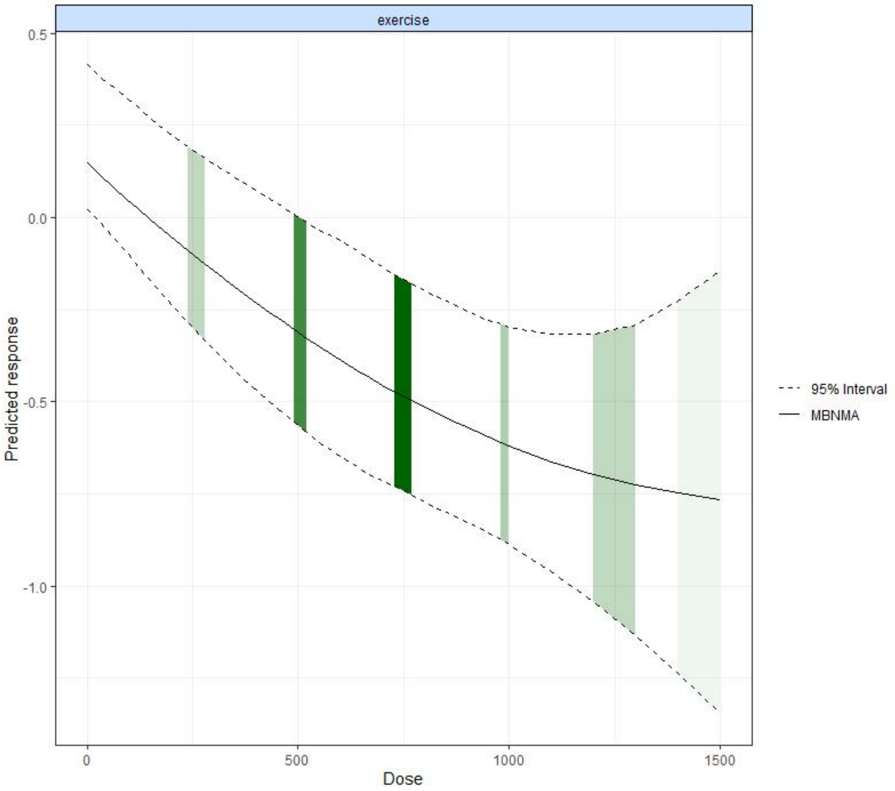
exercise
0.5
0.0
-0.5
-1.0
Predicted response
0
500
1000
1500
Dose
95% Interval
MBNMA

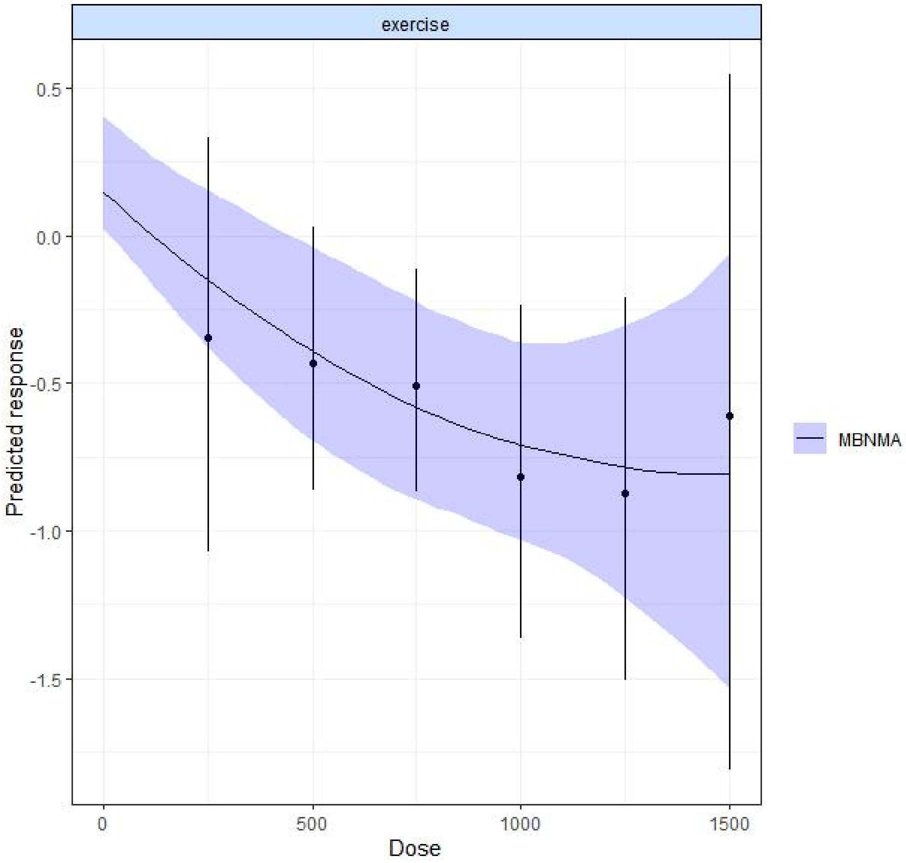
exercise
0.5
0.0
-0.5
-1.0
-1.5
Predicted response
0
500
1000
1500
Dose
MBNMA

FBG

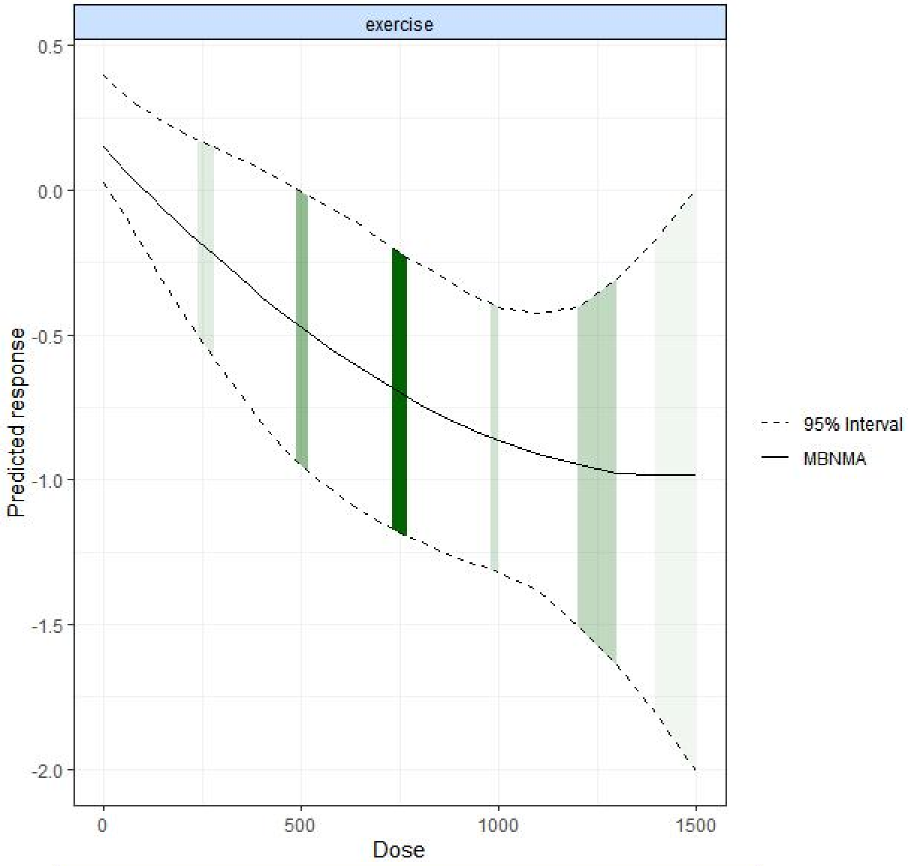
exercise
0.5
0.0
-0.5
-1.0
-1.5
-2.0
Predicted response
0
500
1000
1500
Dose
95% Interval
MBNMA

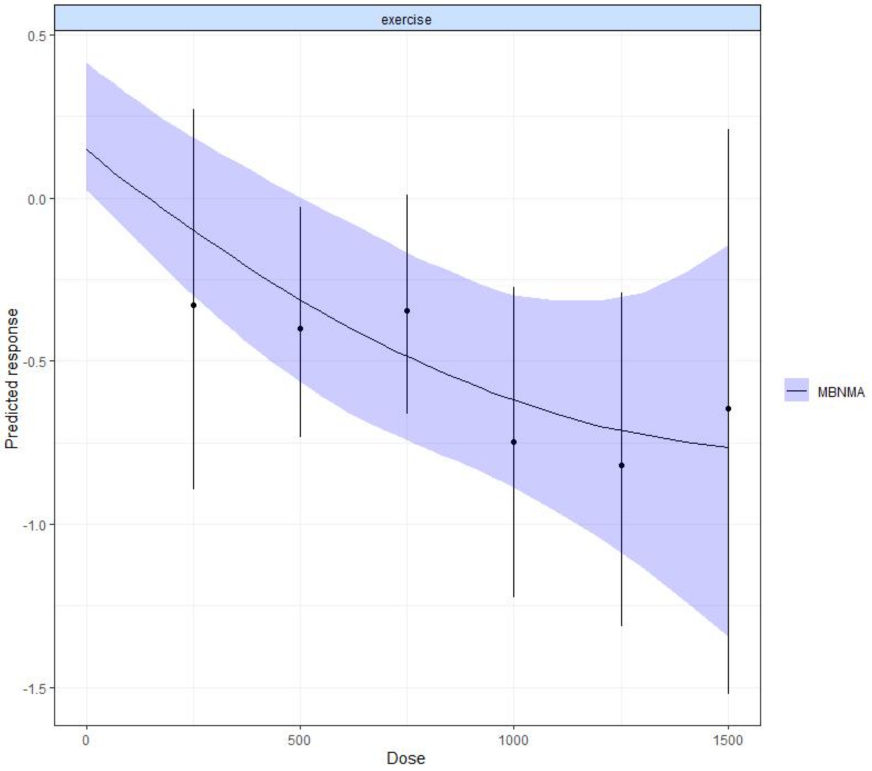
exercise
0.5
0.0
-0.5
-1.0
-1.5
Predicted response
0
500
1000
1500
Dose
MBNMA

Figure S17. Prediction dose-response relationship at agent-level. PI, prediction interval. CG, control group. CAE, continuous aerobic exercise. CE, combined aerobic and resistance exercise. RE, resistance exercise. HIIT, high-intensity interval training. TCS, traditional Chinese sports.

HbA1c

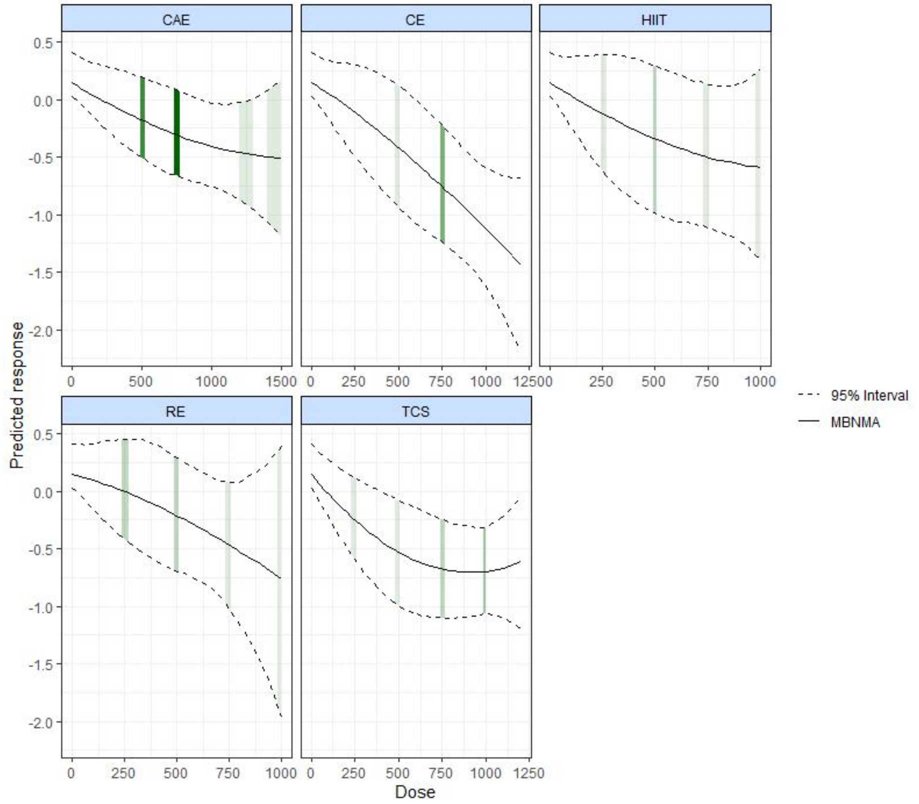

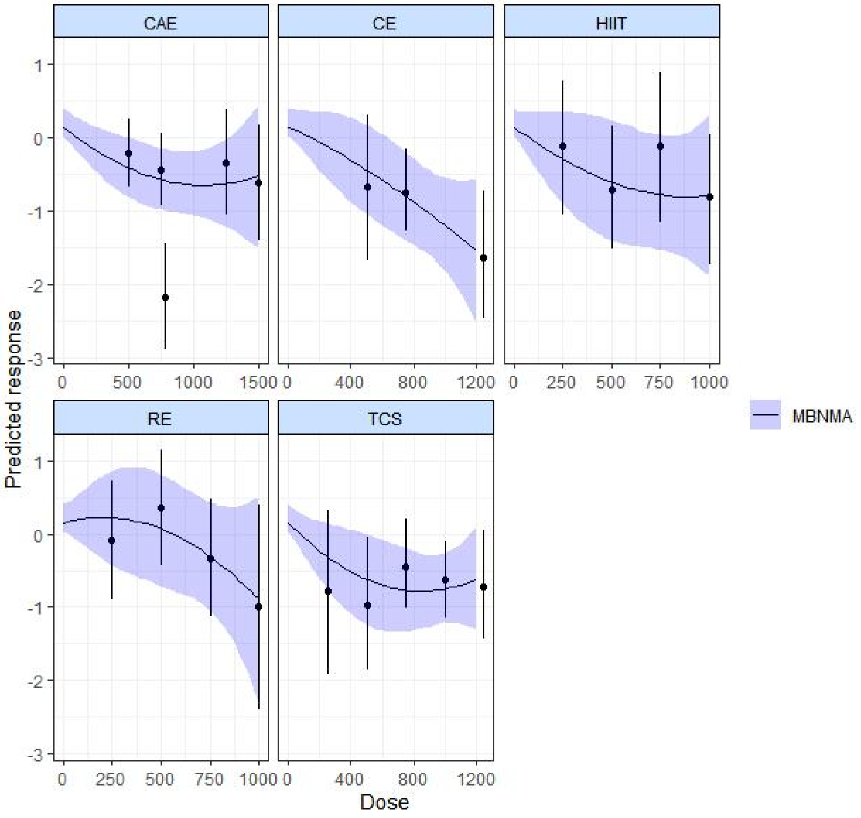

FBG

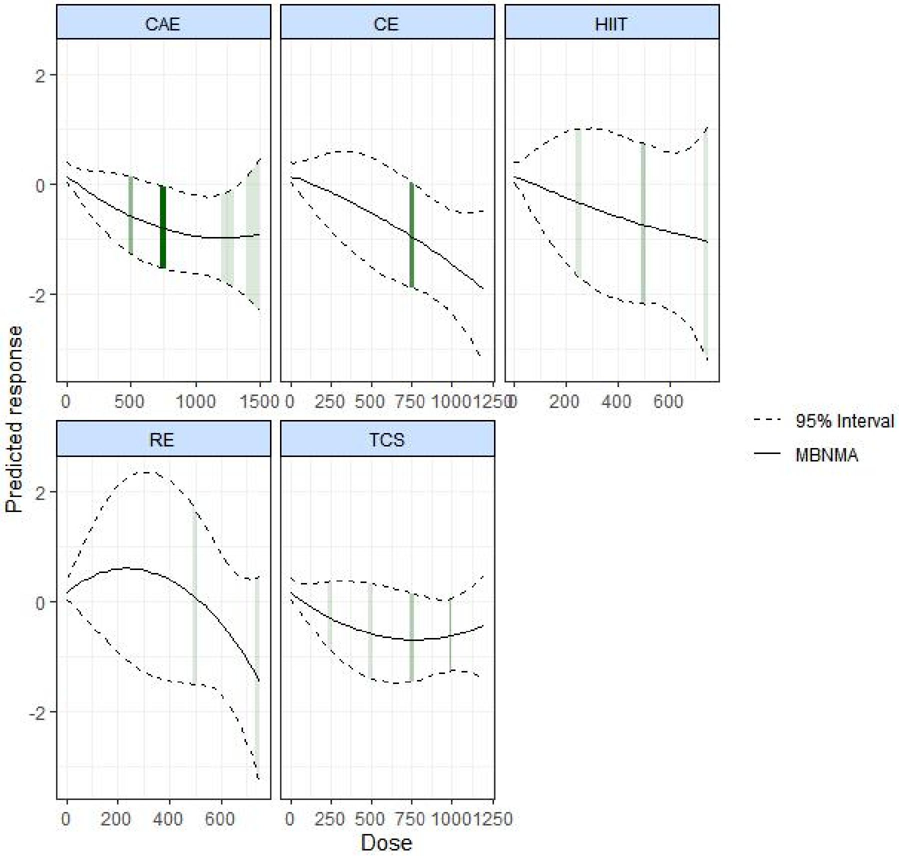

CAE
CE
HIIT
RE
TCS
Predicted response
Dose
95% Interval
MBNMA

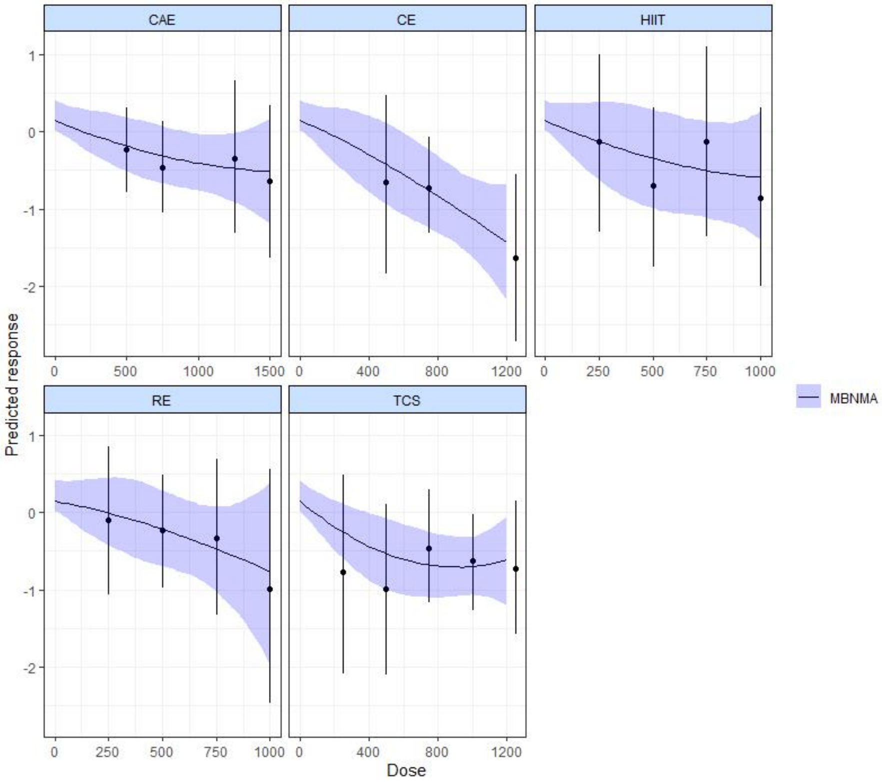

CAE
CE
HIIT
RE
TCS
Predicted response
Dose
MBNMA

Figure S18. Dose-response relationship at agent level. Figure a represents the dose-response + node-split, and the shaded area in Figure b represents the original study dataset; the darker the color, the larger the amount of data. CG, control group. CAE, continuous aerobic exercise. CE, combined aerobic and resistance exercise. RE, resistance exercise. HIIT, high-intensity interval training. TCS, traditional Chinese sports.

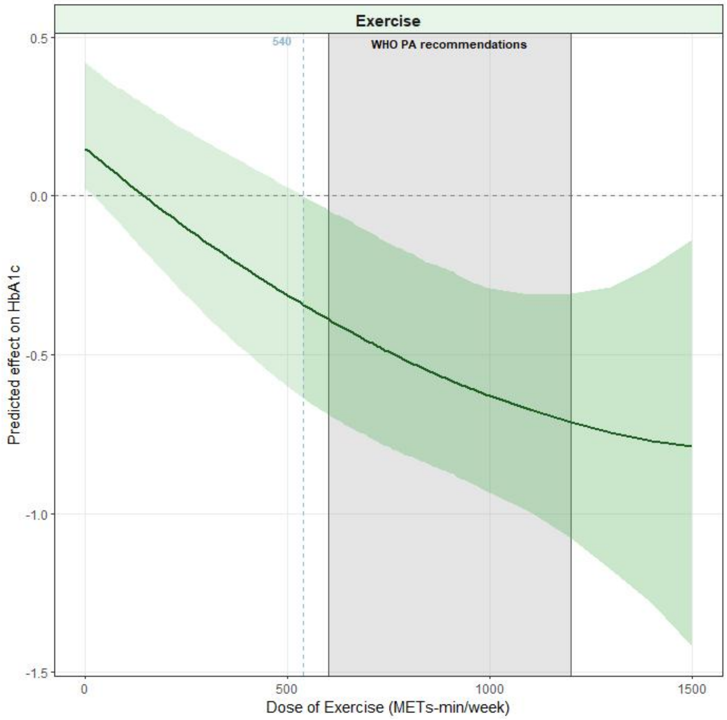

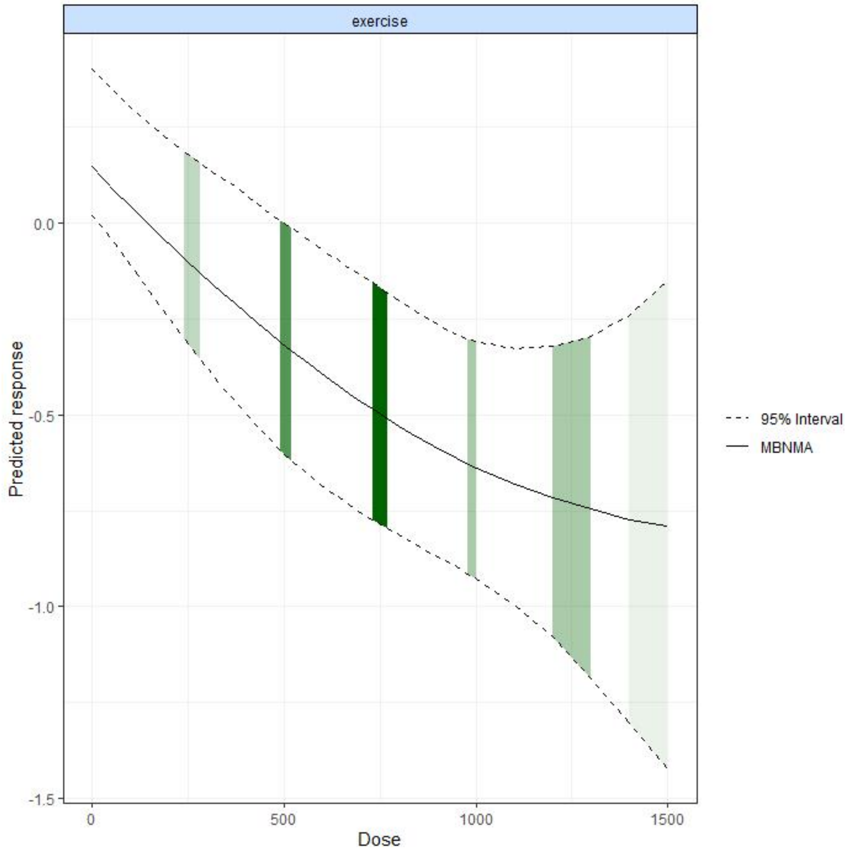

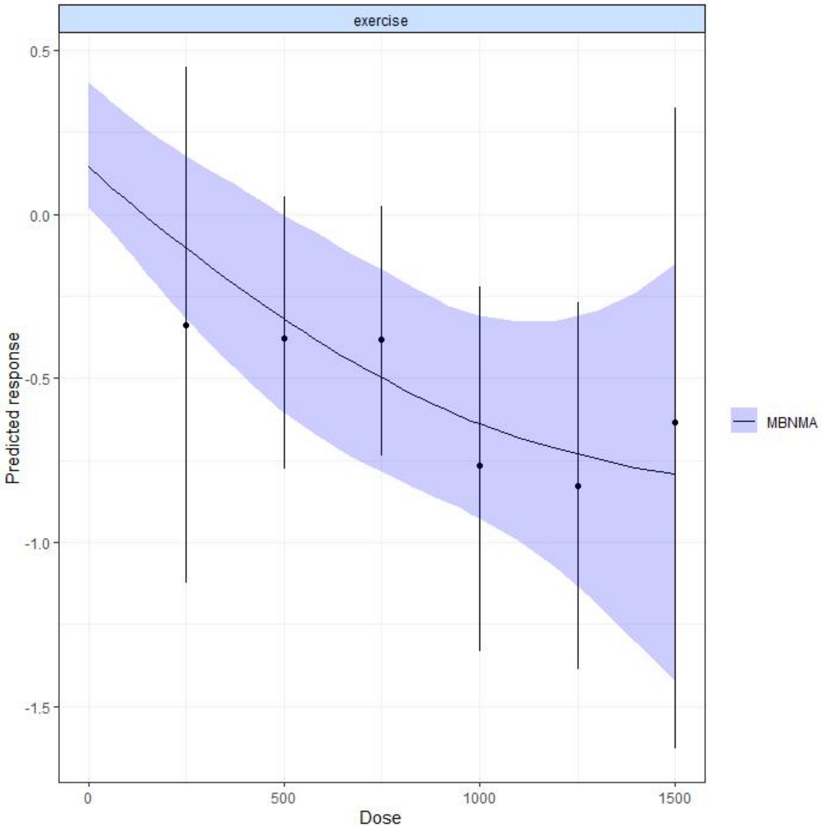

Figure S19. Post hoc sensitivity analysis of the overall dose-response relationship between exercise dose and HbA1c after excluding HIIT arms. The curve shows the predicted effect of weekly exercise dose on HbA1c after removal of HIIT intervention arms. The shaded area represents the 95% uncertainty interval.

7.3.2 Effectiveness ranking of different exercise modalities

Figure S20 shows the probability of each intervention to be ranked from best to worst (estimated after up to 4000 iterations).
HbA1c

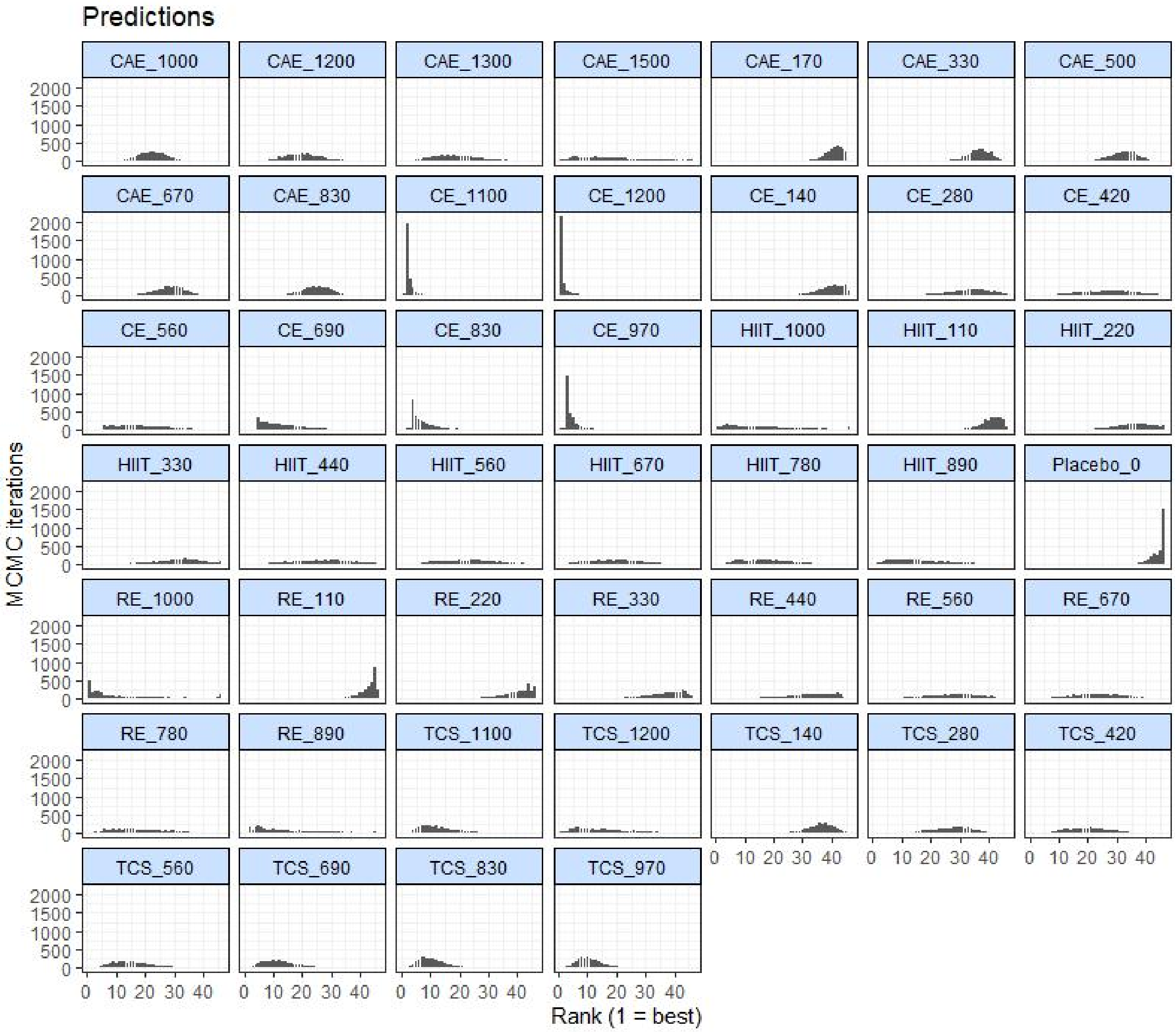

FBG

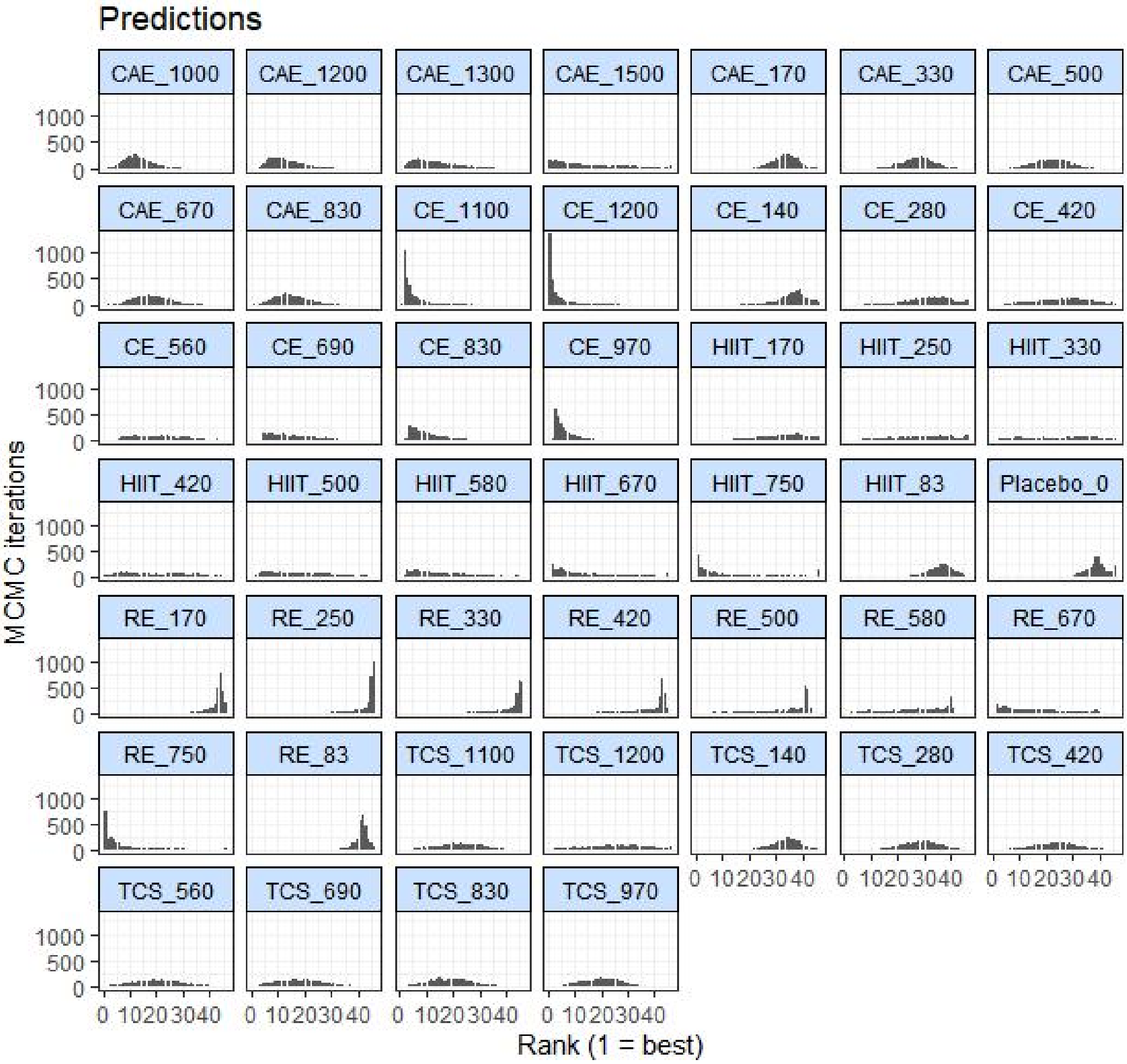

Figure S20. Effectiveness rankings by exercise modality. The number that follows the exercise intervention indicates the dose of exercise (MET-min/week). CG, control group. CAE, continuous aerobic exercise. CE, combined aerobic and resistance exercise. RE, resistance exercise. HIIT, high-intensity interval training. TCS, traditional Chinese sports.

Table S14. Predicted rankings (from best to worst)
HbA1c

Treatment	Mean	Median	2.50%	97.50%
CE_1200	2.09	1	1	11
CE_1100	3.04	2	2	11
CE_970	4.68	3	2	14
CE_830	7.99	6	3	21
TCS_830	10.99	10	3	24
TCS_970	11.11	10	4	22
TCS_690	12.82	12	3	28
TCS_1100	12.88	12	4	29
CE_690	13.37	12	5	31
RE_1000	14.19	8	1	46
TCS_1200	15.53	14	3	37
RE_890	16.06	13	2	44
TCS_560	16.18	15	5	32
HIIT_890	17.13	16	4	39
HIIT_1000	17.52	15	1	46
HIIT_780	17.79	17	4	37
CAE_1500	19.11	17	2	45
RE_780	19.21	18	4	39
CAE_1300	19.37	19	6	37
HIIT_670	19.53	19	5	39
CE_560	19.6	19	6	38
CAE_1200	20.08	20	8	34
TCS_420	21	20	9	36.03
CAE_1000	22.19	22	12	32
HIIT_560	22.23	22	5	42
RE_670	23.33	23	8	39
CAE_830	25.04	25	15	34
HIIT_440	26.12	26	7	44
CE_420	26.65	27	10	43
TCS_280	27.61	28	13	41
RE_560	27.65	29	10	41
CAE_670	28.49	29	18	38
HIIT_330	30.45	31	12	45
RE_440	32.08	33	13	44
CAE_500	32.46	33	22	41
CE_280	33.63	34	17.98	45
HIIT_220	35.31	36	20	46
RE_330	35.96	37	18.98	46
TCS_140	36.08	37	25	44
CAE_330	36.67	37	28	44
RE_220	39.54	41	24	46
CE_140	39.96	41	29	46

HIIT_110	40.38	41	32	45.03
CAE_170	40.85	41	34	45
RE_110	42.61	44	33	46
Placebo_0	44.44	46	38	46

FBG

Treatment	Mean	Median	2.50%	97.50%
CE_1200	4.2	2	1	23
CE_1100	5.21	3	2	20
CE_970	7.44	6	2.98	22
RE_750	10.42	5	1	45
CE_830	11.49	9	2	30
CAE_1200	13.24	12	4	28
CAE_1000	13.36	13	5	25.03
CAE_1300	13.96	12	2.98	34
CAE_830	15.18	15	5	28
HIIT_670	16.18	13	2	44
HIIT_750	16.41	11	1	46
CAE_1500	16.51	14	1	44
CE_690	16.66	15	4	37
HIIT_580	17.16	15	3	41
RE_670	17.23	15	2	41
CAE_670	18.32	18	7	32
TCS_830	18.73	19	6	34
TCS_690	18.8	18	4	36.03
HIIT_500	18.96	17	2	42
TCS_970	20.21	20	7	34
TCS_560	20.78	21	5	39
HIIT_420	21.19	21	2	44
CE_560	21.69	22	6	42
CAE_500	22.6	23	10	36
TCS_1100	22.8	23	7	40
TCS_420	24.25	24	8	41
HIIT_330	24.28	25	3	45
TCS_1200	25.36	26	5	46
RE_580	26.84	29	5	41
CE_420	26.84	27	8	45
HIIT_250	27.62	29	6	46
CAE_330	27.84	28	16	39
TCS_280	28.64	29	14	43
HIIT_170	31.33	33	10	45
CE_280	31.76	33	14	46
RE_500	32.4	37	5	43
CAE_170	33.47	34	24	42
TCS_140	33.8	34	22	44

HIIT_83	35.67	36	21.98	45
RE_420	35.89	42	6	45
CE_140	36.13	37	23	45
RE_330	38.86	44	8	46
Placebo_0	39.09	39	29.98	46
RE_250	40.57	45	12	46
RE_83	40.7	42	29	45
RE_170	40.93	43	19	46

Note: Lower rank values indicate a higher probability of achieving greater glycemic reduction. Values summarize the posterior ranking distribution for each exercise modality and dose level. CAE, continuous aerobic exercise. CE, combined aerobic and resistance exercise. RE, resistance exercise. HIIT, high-intensity interval training. TCS, traditional Chinese sports.
